# The female syndecan-4^−/−^ heart has smaller cardiomyocytes, augmented insulin/pSer473-Akt/pSer9-GSK-3β signaling, and lowered SCOP, pThr308-Akt/Akt and GLUT4 levels

**DOI:** 10.3389/fcell.2022.908126

**Published:** 2022-08-25

**Authors:** Thea Parsberg Støle, Marianne Lunde, Xin Shen, Marita Martinsen, Per Kristian Lunde, Jia Li, Francesca Lockwood, Ivar Sjaastad, William Edward Louch, Jan Magnus Aronsen, Geir Christensen, Cathrine Rein Carlson

**Affiliations:** ^1^ Institute for Experimental Medical Research, Oslo University Hospital and University of Oslo, Oslo, Norway; ^2^ K. G. Jebsen Center for Cardiac Research, University of Oslo, Oslo, Norway; ^3^ Department of Molecular Medicine, Institute of Basic Medical Sciences, University of Oslo, Oslo, Norway

**Keywords:** Syndecan-4, Akt, SCOP/PHLPP1, GLUT4, heart, insulin, sex differences

## Abstract

**Background:** In cardiac muscle, the ubiquitously expressed proteoglycan syndecan-4 is involved in the hypertrophic response to pressure overload. Protein kinase Akt signaling, which is known to regulate hypertrophy, has been found to be reduced in the cardiac muscle of exercised male syndecan-4^−/−^ mice. In contrast, we have recently found that pSer473-Akt signaling is elevated in the skeletal muscle (*tibialis anterior,* TA) of female syndecan-4^−/−^ mice. To determine if the differences seen in Akt signaling are sex specific, we have presently investigated Akt signaling in the cardiac muscle of sedentary and exercised female syndecan-4^−/−^ mice. To get deeper insight into the female syndecan-4^−/−^ heart, alterations in cardiomyocyte size, a wide variety of different extracellular matrix components, well-known syndecan-4 binding partners and associated signaling pathways have also been investigated.

**Methods:** Left ventricles (LVs) from sedentary and exercise trained female syndecan-4^−/−^ and WT mice were analyzed by immunoblotting and real-time PCR. Cardiomyocyte size and phosphorylated Ser473-Akt were analyzed in isolated adult cardiomyocytes from female syndecan-4^−/−^ and WT mice by confocal imaging. LV and skeletal muscle (TA) from sedentary male syndecan-4^−/−^ and WT mice were immunoblotted with Akt antibodies for comparison. Glucose levels were measured by a glucometer, and fasting blood serum insulin and C-peptide levels were measured by ELISA.

**Results:** Compared to female WT hearts, sedentary female syndecan-4^−/−^ LV cardiomyocytes were smaller and hearts had higher levels of pSer473-Akt and its downstream target pSer9-GSK-3β. The pSer473-Akt inhibitory phosphatase PHLPP1/SCOP was lowered, which may be in response to the elevated serum insulin levels found in the female syndecan-4^−/−^ mice. We also observed lowered levels of pThr308-Akt/Akt and GLUT4 in the female syndecan-4^−/−^ heart and an increased LRP6 level after exercise. Otherwise, few alterations were found. The pThr308-Akt and pSer473-Akt levels were unaltered in the cardiac and skeletal muscles of sedentary male syndecan-4^−/−^ mice.

**Conclusion:** Our data indicate smaller cardiomyocytes, an elevated insulin/pSer473-Akt/pSer9-GSK-3β signaling pathway, and lowered SCOP, pThr308-Akt/Akt and GLUT4 levels in the female syndecan-4^−/−^ heart. In contrast, cardiomyocyte size, and Akt signaling were unaltered in both cardiac and skeletal muscles from male syndecan-4^−/−^ mice, suggesting important sex differences.

## Introduction

In response to increased biomechanical stress, the heart is able to physiologically adapt and remodel in an attempt to withstand and compensate for overload (Cooper IV, 1987; Selvetella et al., 2004). Molecular mechanisms driving this adaptive remodeling most likely involve transmembrane proteins that are able to sense extracellular stress and transmit molecular signals intracellularly. One such transmembrane protein is the ubiquitously expressed proteoglycan syndecan-4, which has been found to be involved in the development of concentric hypertrophy in the pressure-overloaded heart (Finsen et al., 2011) and in myofibroblast differentiation and extracellular matrix (ECM) production (Herum et al., 2013). Additionally, shedded syndecan-4 has been found to associate with myocardial infarction (MI) incidence in women (Solbu et al., 2018). Consistent with being a component in the stress-sensing apparatus, syndecan-4 localizes in the costameres and Z-discs (VanWinkle et al., 2002), which are believed to be important structures for sensing stress in the heart. Syndecan-4 may also act as a receptor for growth factors or as a co-receptor with for example integrins, underlying its involvement in a multitude of signaling pathways (Oh et al., 1997; Saoncella et al., 1999; Jang et al., 2012; Elfenbein and Simons, 2013).

The stress-sensing role of syndecan-4 is critically linked to its structure. Like other members of the syndecan family, syndecan-4 is composed of an extracellular domain containing various glycosaminoglycan (GAG) chains, a transmembrane domain, and a short cytoplasmic tail (Couchman et al., 2015). The extracellularly attached heparan sulfate GAG chains can bind to different ligands, including growth factors and components of the ECM (Elfenbein and Simons, 2013). The cytoplasmic tail contains two conserved domains and a variable domain specific to each syndecan. The cytoplasmic tail of the syndecans holds no intrinsic enzymatic activity and is therefore heavily reliant on intracellular binding partners to exert its effects (Elfenbein and Simons, 2013). Several novel intracellular binding partners of syndecan-4 have recently been identified in the heart (Mathiesen et al., 2019).

Supporting a central role of syndecan-4 in stress sensing *via* connections in the ECM, we recently observed that syndecan-4 ablation in skeletal muscle resulted in smaller muscle fibers, alterations in ECM components and an elevated Ser473-Akt phosphorylation (pSer473-Akt) (Rønning et al., 2020). The upregulation of pSer473-Akt was accompanied by an increase in phosphorylation of downstream components of Akt signaling such as mTOR and RPS6 (Rønning et al., 2020). Akt signaling has, since its characterisation, been found to promote growth, metabolism, cell migration, angiogenesis, cell size regulation, cell division and cell cycle progression, largely associated with cancer development and progression (Staal et al., 1977; Altomare and Testa, 2005). In skeletal muscle, Akt activation is linked to prompt muscle mass growth through hypertrophy (Lai et al., 2004). Similarily, in heart muscle, Akt-signaling promotes physiological hypertrophy (Walsh, 2006), and may aid in the initial compensatory responses of the heart during pressure overload (Shiojima et al., 2012).

Akt transduces receptor tyrosine kinase (RTK) signaling, through e.g. the insulin receptor (INSR) and insulin-like growth factor-1 receptor (IGF-1R), or G-protein coupled receptor signaling (Brahmkhatri et al., 2015; Motallebnezhad et al., 2016; Manning and Toker, 2017) and regulates growth through the Akt/mTOR/S6K1 pathway (DeBosch et al., 2006). Interestingly, syndecan-4 has been found to play a central role in this RTK-mediated Akt signaling (Partovian et al., 2008; Ju and Simons, 2013). More specifically, the variable domain of the cytoplasmic tail of syndecan-4 has been found to bind to phosphatidylinositol 4,5-bisphosphate (PIP2), leading to the subsequent recruitment and activation of PKCα, which is involved in Akt signaling (Lee et al., 1998; Horowitz et al., 1999; Lim et al., 2003; Partovian et al., 2008). The role of syndecan-4 in Akt signaling has been further elucidated in studies concluding that it is required for the recruitment of PDK1 and mTOR complex 2 (mTORc2) to lipid rafts in the plasma membrane through PKCα, consequently phosphorylating Thr308-Akt and Ser473-Akt, respectively, fully activating Akt (Partovian et al., 2008; Ju and Simons, 2013). Syndecan-4 ablation reduced both Thr308-Akt and Ser473-Akt phosphorylation, and thus Akt activation, in fibroblast growth factor (FGF) stimulated lung tissue and endothelial cells isolated from lung and heart (Partovian et al., 2008; Ju and Simons, 2013). Reduced pThr308-Akt and pSer473-Akt levels have also been reported in male syndecan-4^−/−^ hearts after 4 weeks of exercise (Xie et al., 2016). In contrast, an elevated pSer473-Akt signaling has been found in the skeletal muscle of female syndecan-4^−/−^ compared to female WT mice (Rønning et al., 2020). These findings raise the possibility that the effects of syndecan-4 ablation on Akt activation are sex dependent.

To determine if the differences seen in Akt activation in syndecan-4^−/−^ mice are sex dependent, we presently investigated Akt signaling in the hearts of female syndecan-4^−/−^ mice. To gain deeper insight into the female syndecan-4^−/−^ heart, cardiomyocyte size, alterations in a wide variety of different ECM components, well-known syndecan-4 binding partners and associated signaling pathways were also investigated. Our data show that the female syndecan-4^−/−^ heart exhibited smaller cardiomyocytes, an elevation in the insulin/pSer473-Akt/pSer9-GSK-3β signaling pathway, lowered levels of SCOP, pThr308-Akt/Akt and GLUT4, and an increased LRP6 level after exercise. Otherwise, few alterations were found. In contrast, cardiomyocyte size, and Akt signaling were unaltered in cardiac and skeletal muscle from male syndecan-4^−/−^ mice, indicating important sex specific differences.

## Methods

### Animal experiments

All animal work was performed in accordance with the approval of the National Regulation on the use of animal research, and the Norwegian Animal Welfare Act (FOTS ID 7695, 15445 and 29268). For the exercise study, we used female syndecan-4^−/−^ and syndecan-4^+/+^ (WT) control mice on a C57BL/6J background (Jackson Laboratory, Bar Harbor, Maine, United States), which were 12 weeks old at harvest. For analyses of glucose, insulin, C-peptide levels, cell size of both sexes and immunoblot analyses of male mice, we used syndecan-4^−/−^ and WT control mice at 13–15 weeks of age (one male syndecan-4^−/−^ mouse used for cell size quantification was 71 weeks old, however cell size was not different from the 13–15 week old mice in the same group) on a C57BL/6J background (Janvier Labs, Le Genest-Saint-Isle, France). Animals were housed in temperature-regulated rooms with 12:12 h light-dark cycles and had access to food and water *ad libitum* (exception during fasting as described below). All animals were sacrificed by cardiac excision during deep surgical anesthesia.

### Exercise training

Female syndecan-4^−/−^ mice and WT controls were exercise trained on a treadmill for rodents (Columbus Instruments, OH, United States) as previously described (Rønning et al., 2020). After 2 days of adaptation to the treadmill, exercise training was carried out with a protocol of 60 min per day for 14 days (including 1 day of rest without training). During each session, all mice performed 3 min of warm-up before six bouts with 8 min of running followed by 2 min of rest. The running speed was set to 14–18 m per minute. Mice unable to complete the protocol were excluded from the study.

### Fasting glucose, insulin and C-peptide measurements

Fasting blood glucose levels were measured by a glucometer (FreeStyle Freedom Lite, Abbott, Oslo, Norway) from the ventral tail artery from female mice after 3 h of fasting. For insulin and C-peptide measurements of female mice, the total blood volume was collected from the chest cavity immediately after cardiac excision. The blood was left at room temperature for 15–20 min to coagulate, before centrifugation at 1500 rcf at 4°C for 10 min. The blood serum was collected and immediately stored at −20°C. Insulin and C-peptide serum concentrations were determined by a mouse insulin ELISA (80-INSMS-E01, Alpco, NH, United States) and mouse C-peptide ELISA (80-CPTMS-E01, Alpco).

### Real-time PCR

LVs were collected from female syndecan-4^−/−^ and WT mice and snap frozen in liquid nitrogen. Tissue (20 mg) was homogenized in RLT lysis buffer (#74106, Qiagen Nordic, Oslo, Norway) containing 1% β-mercaptoethanol (#M3148, Sigma Merck, Darmstadt, Germany) using TissueLyser (2 × 2 min at 25 Hz, #85300, Qiagen Nordic). RNA was isolated using Qiacube with an RNeasy fibrous tissue mini kit (#74106, Qiagen), including DNase (#79254, Qiagen Nordic) and proteinase K (#19131, Qiagen Nordic) treatment according to the manufacturer’s protocol. RNA quality was confirmed using Multiscansky, before cDNA was generated using an iScript cDNA synthesis kit (#1708890, Bio-Rad Laboratories, CA, United States) according to the manufacturer’s protocol. Transcript levels were determined by real-time PCR using TaqMan assays (Table 1) on a QuantStudio 3 detection system (Applied Biosystems by Thermo Fisher Scientific) with 40 two-step cycles (15 s at 95°C, 1 min at 60°C). The relative gene expression (fold change) was calculated from a standard curve and normalized to the average of glyceraldehyde 3-phosphate dehydrogenase (GAPDH) and 60S ribosomal protein L32 (RPL32).

**TABLE 1:** Gene target and TaqMan primer/probe assays.

Gene target	TaqMan primer/probe assays
Decorin	Mm00514535_m1
Fibromodulin	Mm00491215_m1
Biglycan	Mm00455918_m1
Collagen 1a2	Mm00483888_m1
Collagen 3a1	Mm00802331_m1
Syndecan-1	Mm00448918_m1
Syndecan-2	Mm04207492_m1
Syndecan-3	Mm01179833_m1
Syndecan-4	Mm00488527_m1
GAPDH	Mm99999915_g1
Ribosomal protein gene RPL32	Mm02528467_g1

### Immunoblotting

Tissue from the LV or skeletal muscle were homogenized with TissueLyser (#85300, Qiagen Nordic) using ice-cold lysis buffer (20 mM Hepes (pH7.5), 150 mM NaCl, 1 mM EDTA and 0.5% Triton-X100) with added Complete EDTA-free protease inhibitor cocktail (#05056489001, Roche Applied Science, Merck, Darmstadt, Germany) and PhosSTOP (#04906837001, Roche Applies Science). The homogenate was centrifuged at 14,000 rcf for 10 min at 4°C and supernatants were stored at −80°C. Protein concentrations were determined using Micro BCA protein assay kit (#23235, Thermo Fisher Scientific, MA, United States). An equal protein concentration (40 μg, 80 µg or 10 µg) was loaded per lane on 4–15% Criterion TGX precast gels (#5671084, Bio-Rad) before transferring onto PVDF membranes (#1704157, Bio-Rad) using the Trans-Blot Turbo system (Bio-Rad). Membranes were subsequently blocked in 5% non-fat milk, 1 × casein or 5% BSA for 1 h at room temperature. Primary antibodies were then added overnight at 4°C. Following incubation, membranes were washed with TBS-T (Tris-buffered saline with 1% Tween-20 (#1610781, Bio-Rad)) for 20 min followed by two 10-min washes. Secondary HRP-conjugated antibodies were added for 1 h at room temperature before another 20-min wash, followed by four 5-min washes. Blots were developed with ECL prime (#RPN2236, GE Healthcare, IL, United States) and detected by LAS-4000 (Fujifilm, Tokyo, Japan). Membranes were stripped for 5–10 min (#21603, Thermo Scientific, CA, United States) before reprobing.

Antibodies and blocking conditions were as follows:

Anti-LOX (1:500, 1 × casein, sc-32409, Santa Cruz, TX, United States), anti-decorin (1:500, 1 × casein, AF1060, R&D Systems, MI, United States), anti-Wnt4 (1:1000, 1 × casein, ab91226, Abcam, Cambridge, United Kingdom), anti-ZO-1 (1:750, 1 × casein, sc-33725, Santa Cruz), anti-LRP6 (1:1000, 1 × casein, #2560, Cell Signaling, MA, United States), anti-fibromodulin (1:500, 1 × casein, sc-33772, Santa Cruz), anti-biglycan (1:500, 1 × casein, sc-27936, Santa Cruz), anti-PKCα (1:250, 1 × casein, sc-208, Santa Cruz), anti-PTRF/cavin-1 (1:1000, 1 × casein, ab48824, Abcam), anti-TRIM72 (1:750, 1 × casein, sc-514706, Santa Cruz), anti-TPRC7 (1:500, SAB5200051, 1 × casein, Sigma Merck, Darmstadt, Germany), anti-β-catenin (1:5000, 1 × casein, ab32572, Abcam), anti-Frizzled-7 (1:1000, 1 × casein, ab64636, Abcam), anti-pSer2448-mTOR (1:1000, 5% BSA, #2971, Cell Signaling), anti-mTOR (1:1000, 5% BSA, #2983, Cell Signaling), anti-pSer235/236-RPS6 (1:2000, 5% BSA, #4858, Cell Signaling), anti-RPS6 (1:1000, 5% BSA, #2217, Cell Signaling), anti-pSer473-Akt (1:500, 5% BSA, #9271, Cell Signaling), anti-pThr308-Akt (1:500, 5% BSA, #5106, Cell Signaling), anti-Akt (1:500, 5% BSA, #9272, Cell Signaling), anti-CaMKIIδ (1:1000, 1 × casein, GenScript, NJ, United States), anti-SCOP (1:500, 1 × casein, sc-390129, Santa Cruz), anti-pSer9-GSK-3β (1:1000, 5% BSA, #9336, Cell Signaling), anti-GSK-3β (1:1000, 5% BSA, #9315, Cell Signaling), anti-RhoA (1:750, 1 × casein, sc-418, Santa Cruz), anti-pThr202/Tyr204-Erk1/2 (1:1000, 5% BSA, #9101, Cell Signaling), anti-Erk1/2 (1:1000, 5% BSA, #9102, Cell Signaling), anti-syntenin-1 (1:750, 1 × casein, sc-515538, Santa Cruz), anti-PDK1 (1:1000, 1 × casein, ab31406, Abcam), anti-Cleaved Notch1 (1:1000, 5% BSA, #4147, Cell Signaling), anti-MMP2 (1:1000, 1 × casein, RB-1537, Thermo Scientific, CA, United States) anti-pThr70-4E-BP1 (1:1000, 5% BSA, #9455, Cell Signaling), anti-4E-BP1 (1:500, 5% BSA, sc-9977, Santa Cruz), anti-c-SRC (1:250, 1 × casein, sc-8056, Santa Cruz), anti-integrin-β1 (1:1000, 5% milk, MAB 1997, Merck Millipore, Darmstadt, Germany), anti-osteopontin (1:1000, 1 × casein, ab181440, Abcam), anti-RCAN1.4 (1:500, 5% milk, D6694, Sigma Merck), anti-calcineurin (1:200, 1 × casein, sc-9070, Santa Cruz), anti-dynamin-2 (1:1000, 1 × casein, ab3457, Abcam), anti-HES-1 (1:1000, 1 × casein, ab5702, Abcam), anti-CSRP3/MLP (1:1000, 1 × casein, sc-166930, Santa Cruz), anti-GLUT4 (1:1000, 5% milk, #2213, Cell Signaling), anti-GLUT1 (1:1000, 5% BSA, #12939, Cell Signaling) anti-pSer1177-eNOS (1:1000, 5% BSA, #9570, Cell Signaling), anti-eNOS (1:1000, 5% BSA, #32027, Cell Signaling), anti-pSer338-c-Raf (1:1000, 5% BSA, #9427), anti-c-Raf (1:1000, 5% BSA, #9422), anti-GAPDH (1:500, 1 × casein, sc-20357, Santa Cruz), anti-vinculin (1:960,000, 5% milk, V9131, Sigma Merck) and anti-NCX1 (1:1000, 5% milk, Genscript). Secondary antibodies used were anti-rabbit IgG HRP (N934V, Cytiva, MA, United States), anti-mouse IgG HRP (NA931V, Cytiva), and anti-goat IgG HRP (HAF109, R&D Systems). ProBlue SafeStain (Coomassie) (#G00PB001, Giotto Biotech, FI, Italy) verified equal protein loading.

### Fractionation

Snap frozen LVs from female syndecan-4^−/−^ and WT mice were fractioned into cytoplasmic and membrane cellular compartments according to the manufacturer’s protocol (#2145; Merck Millipore, Darmstadt, Germany).

### Cardiomyocyte isolation

Female and male syndecan-4^−/−^ and WT mice were deeply anesthetized by isoflurane inhalation and euthanized by cardiac excision. The aorta was cannulated on a constant flow Langendorff perfusion system. The coronary arteries were flushed with 5–10 ml of isolation buffer (130 mM NaCl, 5.4 mM KCl, 0.5 mM MgCl_2_, 0.4 mM NaH_2_PO_4_, 25 mM HEPES and 5.5 mM D-glucose, pH7.4) at a rate of 2 ml/min to clear the blood. Isolation buffer containing 2 mg/ml collagenase type II was then perfused through the heart at 2 ml/min for 7 min. Once digested, the LV was isolated and minced into chunks in isolation buffer containing 1 mg/ml BSA. The solution was thereafter filtered through a 200 µm filter and cardiomyocytes left to sediment. The Ca^2+^ concentration was gradually increased to 0.2 mM.

Cardiomyocytes were subsequently processed for immunofluorescence as described below, or stimulated with 10 nM insulin for 30 min at room temperature before protein was extracted using ice cold lysis buffer (20 mM Hepes (pH7.5), 150 mM NaCl, 1 mM EDTA and 0.5% Triton-X100). Samples were centrifuged at 14,000 rcf for 5 min at 4°C before the supernatant was collected and stored at −20°C. Samples were then processed for immunoblotting as described, loading 40 µg per lane.

### Immunofluorescence, imaging and processing

Female and male syndecan-4^−/−^ and WT isolated LV cardiomyocytes were fixed in 4% paraformaldehyde for 10 min, quenched in 150 mM glycine for 10 min and permeabilized with 0.5% Triton X-100 for 10 min, at room temperature. Cells were then plated on laminin coated (#L2020, mouse, Sigma Merck) glass bottom dishes (No 1.5, Ø 14 mm, γ-irradiated, Martek Corporation, United States). For visualization, cells were incubated with anti-pSer473-Akt (1:50, #9271, Cell signaling) overnight at 4°C, followed by secondary antibody labeling using goat anti-rabbit Alexa Fluor 647 plus (1:200, #32733, Thermo Fisher Scientific) for 1 h at room temperature. Wheat germ agglutinin (WGA) 488 (1:400, #W11261, Invitrogen) and DAPI 405 (0.1 mg/ml, #MBD0015, Sigma Aldrich) was included in the secondary step to visualize the sarcolemma and nuclei, respectively. Both primary and secondary antibodies were diluted in a blocking buffer consisting of 2% goat serum, 0.1% Triton X-100 and 0.02% NaN_3_ in PBS. Imaging was performed on a ZEISS LSM 800 with Airyscan using the Airyscan super-resolution mode, using a plan-apo 63X 1.4 NA oil objective.

### Statistics

All data are shown as mean ± SEM. Groups (KO-SED versus WT-SED, WT-ET versus WT-SED, KO-ET versus KO-SED and KO-ET versus WT-ET) were compared using the two-tailed Mann-Whitney U tests (GraphPad Prism 8.3.0, La Jolla, CA, United States). Immunoblot quantification was performed in FIJI 1.52p (NIH). A *p*-value of <0.05 was considered statistically significant. Microscopy images comparing mean fluorescence intensity and cardiomyocyte size were processed with built-in software (Zeiss, Jena GmbH, Germany) using Airyscan processing and deconvolution algorithms. Image analysis was subsequently carried out in FIJI, using custom-written macros. Cell fluorescence intensity was quantified as F/F_0_, whereby F, the mean fluorescence intensity of the cell was normalized to the mean background (non-cardiomyocyte area) intensity F_0_. Cardiomyocyte size was defined as the total longitudinal area outlined by WGA staining at the axial midpoint of the cell.

## Results

### Female syndecan-4^−/−^ hearts exhibit altered pSer473-Akt and pThr308-Akt signaling

To investigate the role of syndecan-4 in the female heart, syndecan-4^−/−^ and age-matched female WT mice were subjected to a 2-week long treadmill exercise protocol (ET mice, 12 weeks at harvest) (Rønning et al., 2020). Sedentary female syndecan-4^−/−^ and WT mice of the same age were used as controls (SED mice).

**FIGURE 1:**
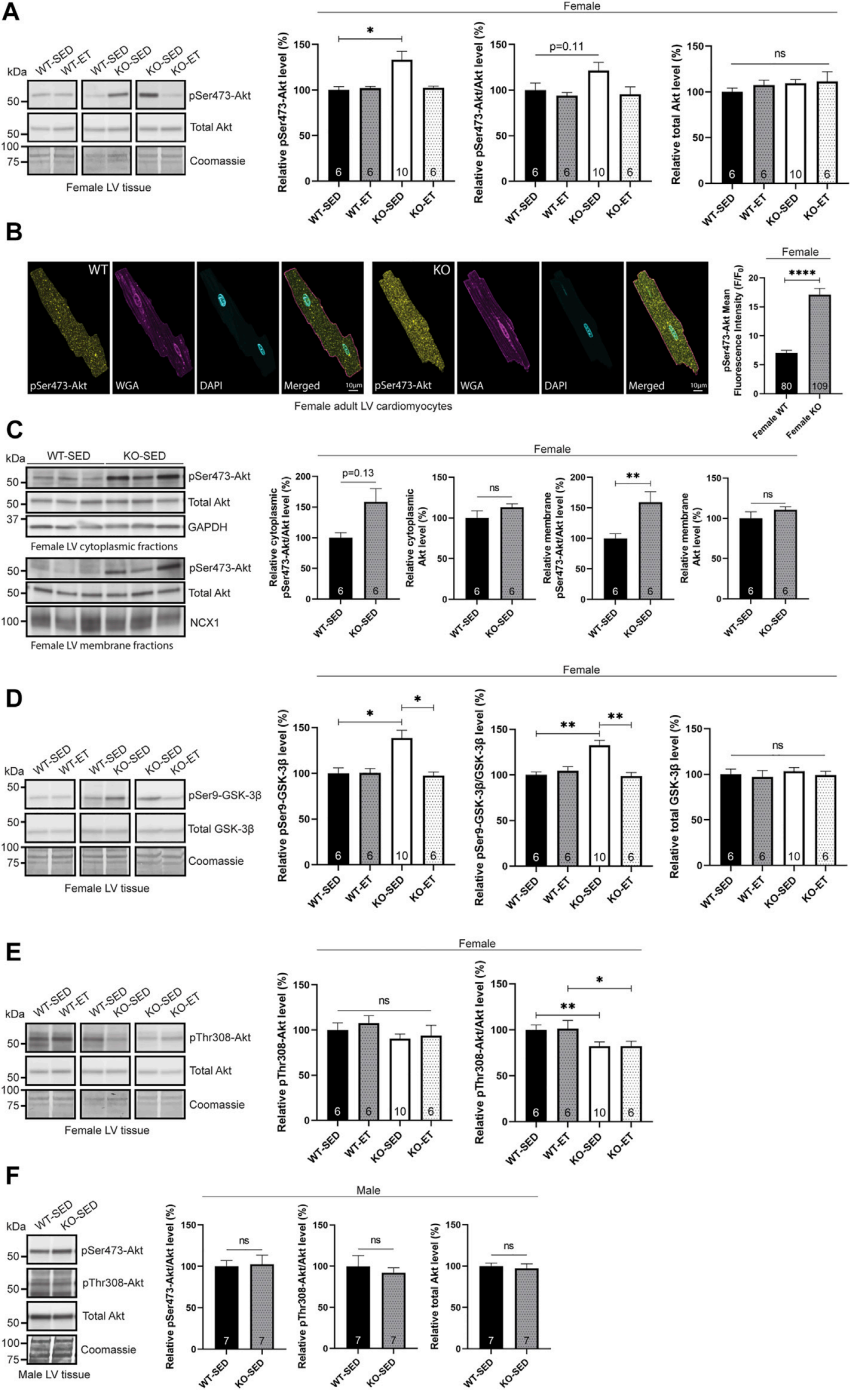
Female syndecan-4^−/−^ hearts exhibit altered pSer473-Akt and pThr308-Akt signaling. **(A)** Immunoblot analysis of pSer473-Akt and Akt in LV of WT-SED, WT-ET, KO-SED and KO-ET female mice. **(B)** Immunofluorescence of pSer473-Akt (yellow) in adult cardiomyocytes isolated from female syndecan-4^−/−^ and WT mice. The cell nuclei were stained with DAPI (cyan) and surface cell membranes with WGA (magenta) (109 cells from *n* = 3 syndecan-4^−/−^ mouse and 80 cells from *n* = 3 WT mice, indicated on the graph bars). **(C)** Immunoblot of pSer473-Akt and total Akt in cytoplasmic and membrane fractions of left ventricular (LV) tissue from female WT-SED and KO-SED mice (quality of the fractionations are shown in Supplementary Figure S1H). **(D)** Immunoblot analysis of pSer-GSK-3β and GSK-3β and **(E)** pThr308-Akt and Akt (same total Akt as in **(A)**) in LV of WT-SED, WT-ET, KO-SED and KO-ET female mice. **(F)** Immunoblot of pSer473-Akt, pThr308-Akt and total Akt in LV from male KO-SED and WT-SED mice. Quantified immunoblot values are presented as mean percentages ± SEM, normalized to the WT-SED mice (*n* = 6–10, indicated on the graph bars). Comparison between the groups were analyzed using Mann-Whitney *U* tests (*p < 0.05, **p < 0.01, ****p < 0.0001). Coomassie staining **(A**,**D**–**F)**, GAPDH [**(C)**, cytoplasmic fraction] and NCX1 [**(C)**, membrane fraction] were used as loading controls for immunoblots.

First, we analysed protein kinase Akt phosphorylation, which we have previously found to be elevated in the female syndecan-4^−/−^ skeletal muscle (*tibialis anterior,* TA) (Rønning et al., 2020). Consistent with our skeletal muscle data, immunoblotting revealed that the level of phosphorylated Ser473-Akt (pSer473-Akt) was also elevated in the sedentary female syndecan-4^−/−^ LV (Figure 1A, female, KO-SED versus WT-SED). The pSer473-Akt level had a trend of returning to WT-SED levels after exercise (Figure 1A, female, KO-ET versus WT-SED). An elevated pSer473-Akt level was also confirmed with immunofluorescence imaging of isolated adult cardiomyocytes, where the mean fluorescence intensity of pSer473-Akt was increased in the female syndecan-4^−/−^ cardiomyocytes compared to WT cardiomyocyte controls (Figure 1B, female, KO versus WT). Additionally, fractionation of LVs revealed that the elevation of pSer473-Akt was apparent in both the cytoplasm (trend) and membrane fractions of the female syndecan-4^−/−^ heart (Figure 1C, female, KO-SED versus WT-SED). Consistent with the elevated pSer473-Akt level, pSer9-GSK-3β (glycogen synthase kinase-3β), a downstream target of pSer473-Akt involved in glycogen storage (Case et al., 2011), was also increased in the female syndecan-4^−/−^ mice (Figure 1D, female, KO-SED versus WT-SED), and similar to pSer473-Akt, it returned to WT-SED levels after exercise (Figure 1D, female, KO-ET versus WT-SED).

Next, we investigated pThr308-Akt, the second phosphorylation site required for full activation of Akt. We have previously observed no significant changes in Thr308-Akt phosphorylation in the skeletal muscle of female syndecan-4^−/−^ mice (Rønning et al., 2020). In the female heart however, pThr308-Akt/Akt was lowered (Figure 1E, female, KO-SED versus WT-SED) and not reversed with exercise (Figure 1E, female, KO-ET versus WT-SED).

We also investigated kinases upstream of Akt activation. Both PDK1 and mTOR complex 2 (mTORc2), which phosphorylate Thr308-Akt and Ser473-Akt, respectively, are recruited to the plasma membrane through syndecan-4-dependent activation of PKCα (Partovian et al., 2008; Ju and Simons, 2013). However, we did not observe any changes at the total protein level of mTOR, PDK1 or PKCα (Supplementary Figures S1A–C, female, KO-SED versus WT-SED), supporting the hypothesis that the total protein levels of these kinases are not changed, but rather that they are delocalized from the plasma membrane in the absence of syndecan-4 (Partovian et al., 2008; Ju and Simons, 2013). Delocalization of PDK1 from the plasma membrane is consistent with the reduced pThr308-Akt/Akt level we observed in the female syndecan-4^−/−^ LVs (Figure 1E, female, KO-SED versus WT-SED), whereas the increased pSer473-Akt level (Figure 1A, female, KO-SED versus WT-SED) had to be promoted by another mechanism other than mTORc2.

Furthermore, we investigated the cardiac activity of the mTOR complex 1 (mTORc1). mTORc1 is a rapamycin-sensitive protein complex consisting of mTOR, mLST8 and raptor, while mTORc2, upstream of Akt activation, includes mTOR, mLST8, rictor, mSin1 and protor (Jhanwar-Uniyal et al., 2019). To measure mTORc1 activity, we analyzed the phosphorylation levels of its two targets RPS6 and 4E-BP1, which associate with cell size, protein translation and survival (Ruvinsky et al., 2005; Yang and Guan, 2007; Zhang et al., 2010). Immunoblotting showed that pSer2448-mTOR/mTOR, pSer235/236-RPS6/RPS6, and pThr70-4E-BP1/4E-BP1 levels were unaltered in female mice sedentary mice (Supplementary Figures S1A,D,E, female, KO-SED versus WT-SED), suggesting that the mTORc1 pathway was not activated in the sedentary female syndecan-4^−/−^ LVs. In contrast, mTORc1 activity has previously been found to be increased in female syndecan-4^−/−^ skeletal muscle (Rønning et al., 2020) and cardiac endothelial cells (Partovian et al., 2008), suggesting differences between cardiac and skeletal muscle, and cell types. Despite unaltered levels at baseline, exercised female syndecan-4^−/−^ LVs had reduced levels of pSer2448-mTOR/mTOR and pSer235/236-RPS6/RPS6 compared to exercised WT mice (Supplementary Figures S1A,D, female, KO-ET versus WT-ET), indicating a potential shift from mTORc1 to mTORc2 after exercise in these hearts. Interestingly, mTORc2 activity has been found to increase after exercise in the skeletal muscle (Kleinert et al., 2017).

Lastly, to investigate if the altered phosphorylation levels of Akt in the female syndecan-4^−/−^ mice were sex-specific, we also examined Akt activation in male syndecan-4^−/−^ and age-matched WT mice. Compared to male WT, there were no significant changes in the pThr308-Akt or pSer473-Akt levels in LV (Figure 1F, male, KO-SED versus WT-SED) or skeletal muscle (TA) (Supplementary Figure S1F, male, KO-SED versus WT-SED) from the male syndecan-4^−/−^ mice.

Taken together, our data suggest altered pSer473-Akt and pThr308 signaling in female, but not in male syndecan-4^−/−^ mice.

### Female syndecan-4^−/−^ hearts exhibit alterations in SCOP phosphatase, fasting C-peptide and GLUT4 levels

**FIGURE 2:**
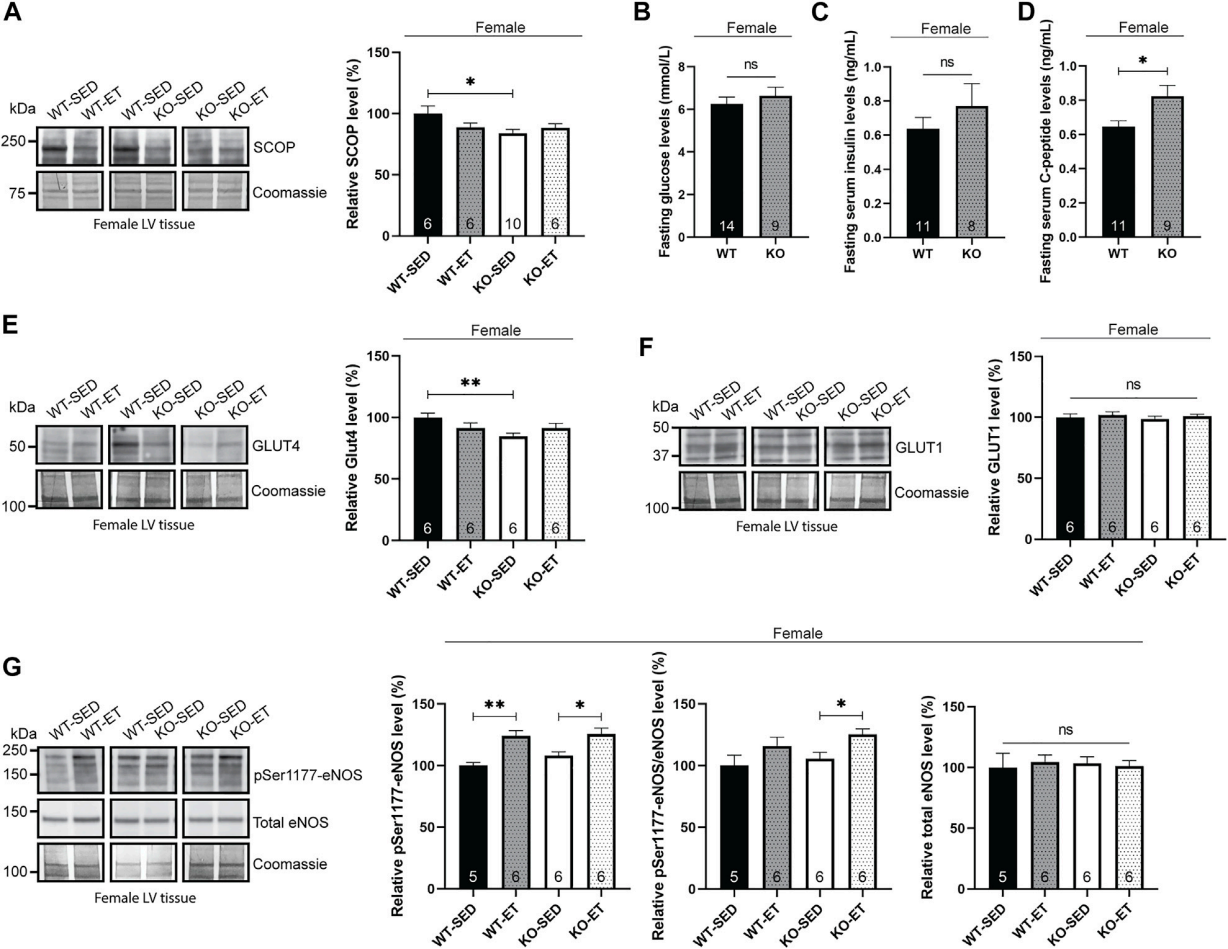
Female syndecan-4^−/−^ hearts exhibit alterations in SCOP phosphatase, fasting C-peptide and GLUT4 levels. **(A)** Immunoblot analysis of SCOP in LV of WT-SED, WT-ET, KO-SED and KO-ET female mice. **(B)** Fasting serum glucose levels, **(C)** insulin and **(D)** C-peptide levels in female syndecan-4^−/−^ and WT mice (*n* = 8–14, indicated on the graph bars). Immunoblot analyses of **(E)** GLUT4, **(F)** GLUT1 and **(G)** pSer1177-eNOS and total eNOS in LV of WT-SED, WT-ET, KO-SED and KO-ET female mice. Quantified immunoblot values are presented as mean percentages ± SEM, normalized to the WT-SED (*n* = 5–10, indicated on the graph bars). Coomassie staining **(A**,**E**–**G)** was used as loading controls for immunoblots. Glucose levels are presented in mmol/L **(B)** and insulin and C-peptide values are presented in ng/mL **(C**,**D)**. Comparison between the groups were analyzed using Mann-Whitney *U* tests (*p < 0.05 and **p < 0.01).

Next, we aimed to investigate the mechanisms underlying the increased pSer473-Akt level observed in the female syndecan-4^−/−^ heart. In absence of syndecan-4, the likely delocalization of the Ser473-Akt phosphorylating kinase, mTORc2, from the plasma membrane means that it can no longer contribute to Akt activation. Consequently, we hypothesized that the reduction of a phosphatase rather promotes the upregulation of pSer473-Akt. Interestingly, the suprachiasmatic nucleus circadian oscillatory protein (SCOP), also called PH domain and leucine repeat protein phosphatase 1 (PHLPP1), has been identified as one such phosphatase, responsible for dephosphorylating pSer473-Akt (Miyamoto et al., 2010). Compared to WT hearts, immunoblotting showed that SCOP was indeed lowered in female syndecan-4^−/−^ hearts (Figure 2A, female, KO-SED versus WT-SED), thus likely contributing to the increase in pSer473-Akt levels. We did not detect changes in the levels of another SCOP/PHLPP1 target, pSer338-c-Raf/c-Raf, in the female syndecan-4^−/−^ heart (Supplementary Figure S1G, female, KO-SED versus WT-SED) (Li et al., 2014).

Interestingly, SCOP degradation has been inversely correlated with serum insulin levels (Xing et al., 2016). We therefore anticipated that the female syndecan-4^−/−^ mice may exhibit elevated levels of serum insulin. To prevent glucose-mediated variations on serum insulin, mice were fasted for 3 h prior to blood sampling. Fasting blood glucose levels were not significantly different between female WT and syndecan-4^−/−^ mice (Figure 2B, female, KO versus WT). Consistent with our hypothesis, we observed a tendency for higher insulin levels in female syndecan-4^−/−^ mice compared to WT (Figure 2C, female, KO versus WT). Since insulin does not have long-term stability in the serum and is to a high extent metabolized by the liver we also measured C-peptide, which has a higher half-life and is a more reliable measurement of insulin (Jones and Hattersley, 2013). C-peptide is secreted as a part of proinsulin from the pancreas and is released at equivalent concentration as insulin after proinsulin cleavage (Jones and Hattersley, 2013). Female syndecan-4^−/−^ mice had higher fasting C-peptide levels compared to WT controls (Figure 2D, female, KO versus WT).

One of the functional molecular outcomes of insulin-Akt signaling is the translocation of glucose transporters from intracellular vesicles to the cell membrane to facilitate glucose influx into the cell (Matsui et al., 2006). We therefore investigated the levels of GLUT4, the main insulin-responsive glucose transporter in the heart (Shao and Tian, 2015). Immunoblotting of GLUT4 showed two distinct bands of the appropriate molecular weight at approximately 50 kDa. Previous reports of multiple bands of GLUT4 immunoblotting have attributed them to the glycosylation state of the glucose transporter (Fischer et al., 1997; Zaarour et al., 2012). Compared to WT, the female syndecan-4^−/−^ hearts did not display the lower of the two 50 kDa bands and consequently had lower levels of total GLUT4 (Figure 2E, female, KO-SED versus WT-SED). Previous work has found an increase in GLUT1 in adipocytes with reduced GLUT4 in response to chronic insulin-Akt signaling (Sargeant and Pâquet, 1993). As others have demonstrated, we also observed three distinct bands in GLUT1 immunoblotting between 37 and 50 kDa (Fischer et al., 1997). However, the levels of GLUT1 were not altered in the female syndecan-4^−/−^ hearts compared to WT controls (Figure 2F, female, KO-SED versus WT-SED).

Finally, we investigated the levels of eNOS, which is phosphorylated at Ser1177 (pSer1177-eNOS) by Akt in response to exercise (Fulton et al., 1999; Dao et al., 2011) and is associated with blood-pressure control (Huang et al., 1995). Consistent with a previous report (Dao et al., 2011), we found that pSer1177-eNOS increased in exercise trained female syndecan-4^−/−^ and WT mice compared to sedentary mice (Figure 2G, female, KO-ET versus KO-SED and WT-ET versus WT-SED). However, no differences in pSer1177-eNOS or total eNOS were observed between the two genotypes at baseline (Figure 2G, female, KO-SED versus WT-SED).

Taken together, our data indicate that the altered pSer473-Akt and pThr308-Akt signaling in the female syndecan-4^−/−^ hearts is accompanied by changes in the insulin/SCOP/pSer9-GSK-3β pathway, as well as reduced GLUT4 levels.

### Female syndecan-4^−/−^ cardiomyocytes are smaller than WT

**FIGURE 3:**
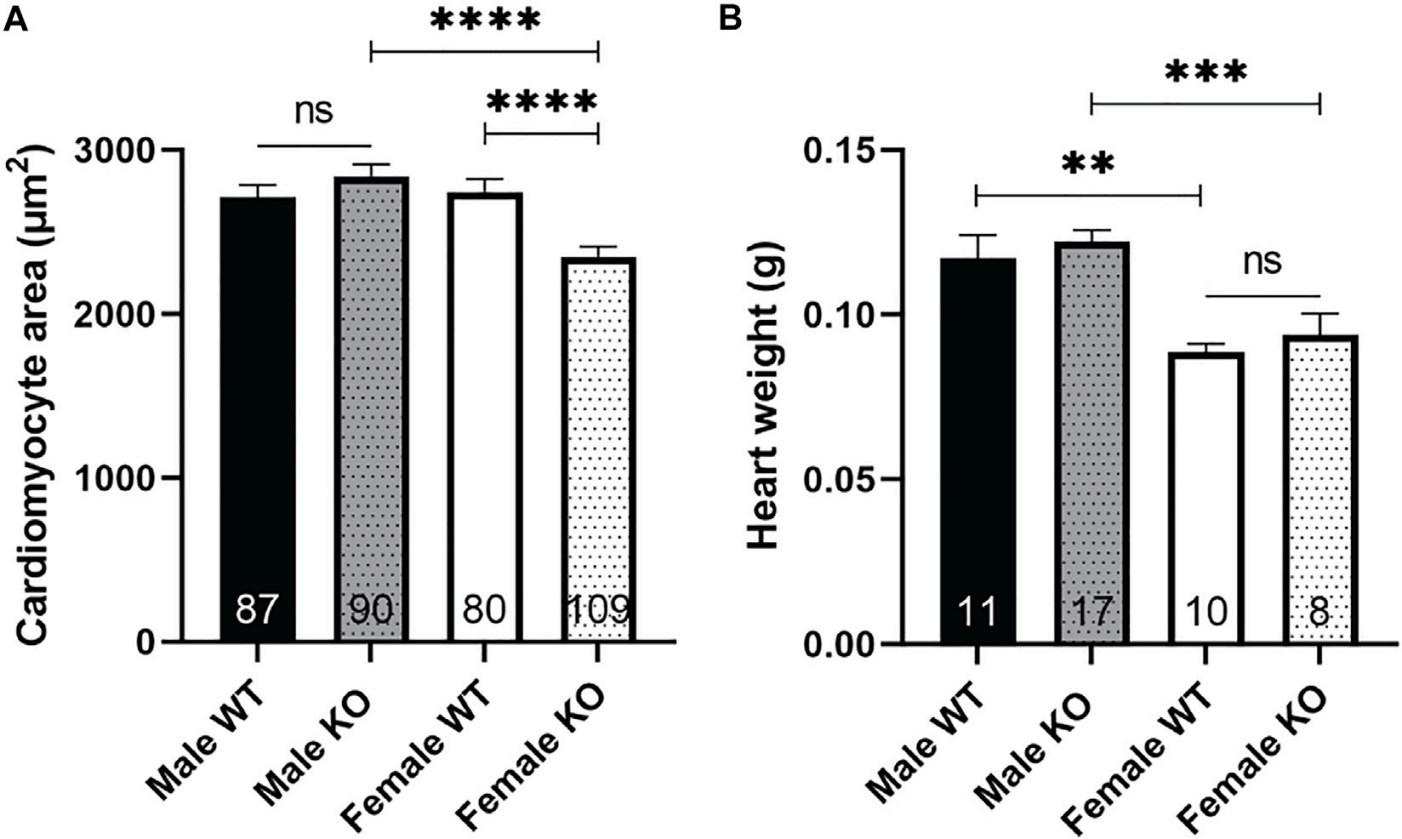
Female syndecan-4^−/−^ cardiomyocytes are smaller than WT. **(A)** Cardiomyocyte area of female and male syndecan-4^−/−^ and WT mice (87 cells from *n* = 3 male WT, 90 cells from *n* = 3 male syndecan-4^−/−^, 80 cells from *n* = 3 female WT, and 109 cells from *n* = 3 female syndecan-4^−/−^ mice, indicated on the graph bars). **(B)** Heart weight of female and male syndecan-4^−/−^ and WT mice (*n* = 8–17, indicated on the graph bars). Comparison between the groups were analyzed using Mann-Whitney *U* tests (**p < 0.01, ***p < 0.001, ****p < 0.0001).

One of the functional effects of increased Akt signaling is an increase in cell size (Condorelli et al., 2002). However, we have previously found decreased body weight, skeletal muscle weight and fiber size in female syndecan-4^−/−^ (Rønning et al., 2020). Consistent with these results, we also found reduced cardiomyocyte size in female syndecan-4^−/−^ compared to female WT mice (Figure 3A, female, KO versus WT). Surprisingly, the heart weight was not altered in female syndecan-4^−/−^ compared to female WT hearts (Figure 3B, female, KO versus WT). In contrast, male syndecan-4^−/−^ cardiomyocyte area and heart weight were not altered compared to WT, consistent with previous findings (Romaine et al., 2022) (Figures 3A,B, male, KO versus WT).

### Female syndecan-4^−/−^ hearts have no alterations in ECM components

**FIGURE 4:**
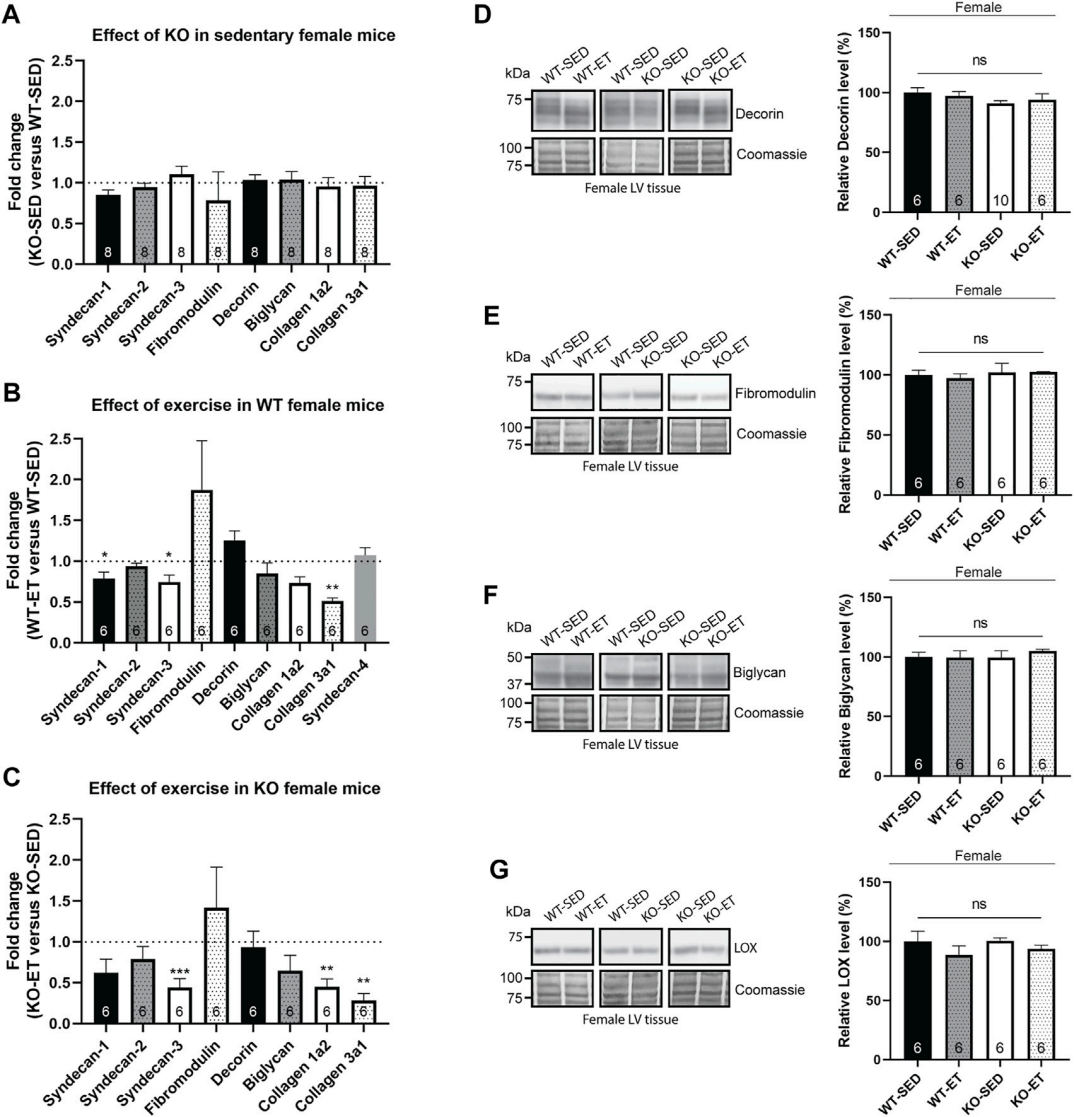
Female syndecan-4^−/−^ hearts have no alterations in ECM components. Bars show relative mRNA levels in the female LV from **(A)** KO-SED versus WT-SED, **(B)** WT-ET versus WT-SED, and **(C)** KO-ET versus KO-SED mice. WT-SED is set to 1 in **(A**,**B)**, and KO-SED is set to 1 in **(C)**, represented by a dotted line (*n* = 6–8, indicated on the graph bars). Comparison between KO-SED/WT-SED, WT-ET/WT-SED and KO-ET/KO-SED were analyzed using Mann-Whitney U tests (*p < 0.05, **<0.01 and ***<0.001). Immunoblot analysis of **(D)** decorin, **(E)** fibromodulin, **(F)** biglycan and **(G)** LOX in the LV from WT-SED, WT-ET, KO-SED and KO-ET female mice. Quantified immunoblot values are presented as mean percentages ± SEM, normalized to the WT-SED mice (*n* = 6–10, indicated on the graph bars). Comparison between the groups were analyzed using Mann-Whitney *U* tests. Coomassie staining was used as a loading control for immunoblots **(D**–**G)**.

We have previously found alterations in several ECM components in the female syndecan-4^−/−^ skeletal muscle (*tibialis anterior*, (Rønning et al., 2020)). However, in the female syndecan-4^−/−^ heart, the mRNA levels of syndecan-1, syndecan-2, syndecan-3, fibromodulin, decorin, biglycan, collagen 1a2 and collagen 3a1 remained unaltered (Figure 4A, female, KO-SED versus WT-SED). Moreover, alterations observed upon exercise were similar for both genotypes. Specifically, syndecan-1 (only a trend in KO-ET), syndecan-3, collagen 1a2 (only a trend in WT-ET) and collagen 3a1 appear to be reduced in both genotypes after exercise, whereas no changes were seen in syndecan-2, fibromodulin, decorin, or biglycan (Figure 4B, female, WT-ET versus WT-SED, and Figure 4C, female, KO-ET versus KO-SED). Syndecan-4 mRNA levels were unaltered after exercise, and was as expected only detected in the WT mice (Figure 4B, female, WT-ET versus WT-SED). Similar observations were made for protein expression, as immunoblotting confirmed unaltered levels of decorin, fibromodulin, biglycan and LOX in the female syndecan-4^−/−^ hearts compared to WT controls, both in sedentary and exercise trained mice (Figures 4D–G, female, KO-SED and KO-ET versus WT-SED and WT-ET).

Taken together, in contrast to skeletal muscle, no alterations were detected in ECM components in the female syndecan-4^−/−^ and WT hearts. Exercise reduced the mRNA levels of syndecan-1 (only a trend in KO-ET), syndecan-3, collagen 1a2 (only a trend in WT-ET) and collagen 3a1 in both genotypes.

### Female syndecan-4^−/−^ hearts have few alterations in protein levels of syndecan-4 binding partners or syndecan-4 associated signaling pathways

In order to gain a deeper understanding of the female syndecan-4^−/−^ heart, we immunoblotted a wide variety of syndecan-4 binding partners recently identified in the hearts (LVs) of male WT mice (Mathiesen et al., 2019), and of other syndecan-4 associated pathways including Wnt (Astudillo et al., 2014), calcineurin-nuclear factor of activated T-cells (NFAT) (Finsen et al., 2011; Herum et al., 2013), osteopontin (Herum et al., 2020) and protein kinase C/transient receptor potential canonical 7 (TRPC7) (Gopal et al., 2015).

Compared to WT controls, immunoblotting showed that the low-density lipoprotein receptor-related protein (LRP6) was upregulated in hearts from female exercise trained syndecan-4^−/−^ mice (Supplementary Figure S2A, female, KO-ET versus KO-SED). LRP6 is known for its involvement in Wnt signaling where it acts as a co-receptor to the frizzleds for Wnt ligands (Liu et al., 2020). Exercise also appeared to increase Wnt4 level in the syndecan-4^−/−^ mice compared to exercised WT (Supplementary Figure S2B, female, KO-ET versus WT-ET). No differences were detected in other syndecan-4 associated signaling pathways (Supplementary Figures S2C–H) or syndecan-4 binding partners (Supplementary Figures S3A–N). These include β-catenin, Frizzled-7, TPRC7, HES-1, Cleaved Notch1, extracellular signal-regulated kinase 1/2 (Erk1/2), calcium (Ca^2+^)/calmodulin (CaM)-dependent protein kinase type II delta (CaMKIIδ), syntenin-1, integrin-β1, transforming protein RhoA (RhoA), caveolae-associated protein 1 (cavin-1), proto-oncogene tyrosine-protein kinase Src (c-SRC), calcineurin, dynamin-2, 72 kDa type IV collagenase (MMP2), tripartite motif-containing protein 72 (TRIM72), muscle LIM protein (MLP), regulator of calcineurin 1 isoform 4 (RCAN1.4), tight junction protein Zo-1 (ZO-1) and osteopontin.

Overall, our data indicate that apart from an increase in LRP6 in exercise trained syndecan-4^−/−^ mice, few other alterations in syndecan-4 associated partners were observed in the female syndecan-4^−/−^ heart.

## Discussion

**FIGURE 5:**
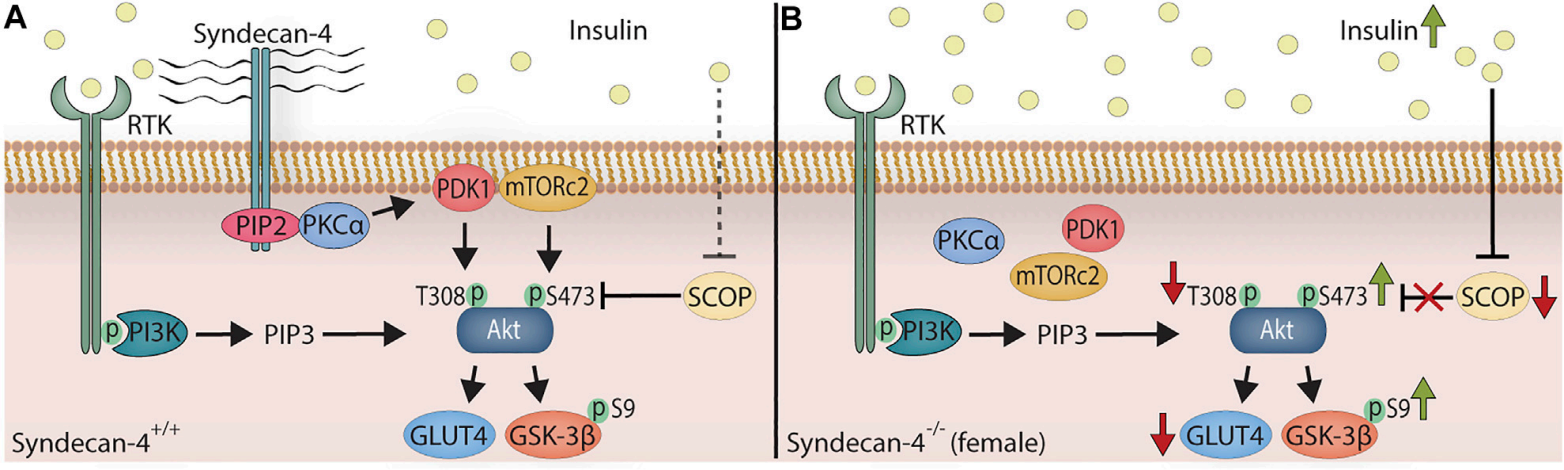
The female syndecan-4^−/−^ heart exhibits smaller cardiomyocytes, an elevated insulin/pSer473-Akt/pSer9-GSK-3β signaling, and lowered SCOP pThr308-Akt/Akt and GLUT4 levels. **(A)** In WT mice, insulin binds to RTK (e.g. insulin receptor), which leads to the activation of PI3K. From here, PIP3 is generated through the phosphorylation of PIP2, binding to the PH domain of Akt, leading to the membrane localization of Akt (not shown for simplicity). PIP2 and syndecan-4 form a complex, recruiting and activating PKCα. PDK1 and mTORc2 are consequently localized to the plasma membrane, phosphorylating Akt at Thr308-Akt (T308) and Ser473-Akt (S473), respectively. Downstream targets of Akt are Ser9-GSK-3β (S9) phosphorylation and GLUT4. The SCOP phosphatase, controlled by insulin, dephosphorylates pSer473-Akt. **(B)** In female syndecan-4^−/−^ mice, the serum insulin levels are chronically elevated. In absence of syndecan-4, PKCα, PDK1 and mTORc2 are delocalized from the membrane resulting in a lack of syndecan-4-dependent Akt phosphorylation. The elevated insulin level depress SCOP phosphatase expression, leading to an increase in syndecan-4-independent phosphorylation of Ser473-Akt (S473) and Ser9-GSK-3β (S9). In absence of syndecan-4, the levels of Thr308-Akt (T308) phosphorylation (PDK1 target) and GLUT4 expression are also lowered, and the cardiomyocytes are smaller (not shown for simplicity).

In this study we have investigated the female syndecan-4^−/−^ heart and analyzed molecules involved in the Akt signaling pathway and a wide variety of different ECM components, well-known syndecan-4 binding partners and associated signaling pathways, both at baseline and in response to exercise. Compared to WT hearts, we found that the sedentary female syndecan-4^−/−^ heart had smaller cardiomyocytes, an elevated insulin/pSer473-Akt/pSer9-GSK-3β signaling pathway, lowered SCOP, pThr308Akt/Akt and GLUT4 levels (Figure 5), and an increased LRP6 level after exercise. Otherwise, few other alterations were found. The pSer473-Akt and pThr308-Akt levels were unaltered in the cardiac and skeletal muscles from sedentary male syndecan-4^−/−^ mice. Additionally, cardiomyocyte size remained unchanged in male syndecan-4^−/−^ mice, suggesting important sex differences.

The underlying mechanisms controlling the sustained alterations in Akt signaling in the female syndecan-4^−/−^ mice may be attributed to activating kinases or inhibitory phosphatases. In Akt signaling, PKCα activation by syndecan-4, recruits PDK1 and mTORc2 to lipid rafts in the plasma membrane, which further phosphorylate Thr308-Akt and Ser473-Akt, respectively (Partovian et al., 2008; Ju and Simons, 2013). While our data indicate that mTORc2 and PDK1 levels remained unaltered, the two kinases were likely not able to mediate Akt phosphorylation due to their delocalization from lipid rafts in the plasma membrane in the absence of syndecan-4 (Partovian et al., 2008). However, the pSer473-Akt phosphatase SCOP, which is reduced by insulin (Xing et al., 2016), was lowered in the female syndecan-4^−/−^ heart. SCOP ablation has been indicated to be cardioprotective in ischemia-reperfusion (Miyamoto et al., 2010), diabetic (Zhang et al., 2020) and aged hearts (Xing et al., 2016). Another substrate downstream of SCOP, pSer338-c-Raf, was not altered in the female syndecan-4^−/−^ heart compared to WT. Supporting this finding, others have found unaltered levels of c-Raf phosphorylation in the heart of SCOP-deficient mice, despite elevated pSer473-Akt levels (Moc et al., 2015). Furthermore, it is worth mentioning that pSer473-Akt and pThr308-Akt can also be dephosphorylated by protein phosphatase 2A (PP2A) (Srinivasan and Begum, 1994; Rodgers et al., 2011). Insulin has been shown to indirectly induce Tyr307-PP2A phosphorylation and thereby inhibit its activity; however after chronic insulin stimulation, phosphorylation is less effective and PP2A activity is preserved (Beg et al., 2016). Although we cannot exclude that PP2A is also involved in the regulation of the pSer473-Akt and pThr308-Akt levels, it is less likely, since the two phosphorylation sites were inversely regulated in the syndecan-4^−/−^ mice. It is more plausible that the reduced pThr308-Akt/Akt levels in the female syndecan-4^−/^ hearts were due to the delocalization of PDK1 from lipid rafts. While our data are in agreement with previous literature of syndecan-4 mediated localization of PDK1 and mTORc2 in cardiac endothelial cells, we have not tested this in isolated cardiomyocytes. Others have, however, found decreased pSer473-Akt and pThr308-Akt in the male syndecan-4^−/−^ heart after exercise, suggesting syndecan-4 is involved in the localization of mTORc2 and PDK1 also in cardiomyocytes, at least after exercise (Xie et al., 2016). Despite supportive literature, we cannot discard the possibility that other kinases also contribute to the phosphorylation of Ser473-Akt. Integrin-linked kinase (ILK) has also been shown to phosphorylate Ser473-Akt and the downstream target GSK-3β, and similarly, cardiac specific deletion of ILK reduces Ser473-Akt phosphorylation (Delcommenne et al., 1998; White et al., 2006). Additionally, ILK activity has been found to be stimulated by insulin in both neuronal and endothelial cell lines (Delcommenne et al., 1998; Tan et al., 2019).

The elevated insulin levels observed in female syndecan-4^−/−^ mice likely result from a global defect in glucose metabolism, in tissues like muscles, adipose and liver, where glucose is the main energy source. The insulin receptor (INSR) has been shown to form hybrid receptors with insulin-like growth factor 1 (IGF-1) receptor (IGF-1R), and be activated by IGF-1, IGF-2 and insulin (Brahmkhatri et al., 2015). Importantly, the metabolic actions of insulin and IGF-1 are mediated through the PI3K-Akt pathway, where at least IGF-1 signaling has been shown to depend on syndecan-4 (Ju and Simons, 2013). Our data suggest that insulin signaling mediated through the PI3K-Akt pathway likely also involves syndecan-4. Interestingly, growth factors like insulin have been reported to cross talk with estrogen receptor α (ERα) signaling (Ueda et al., 2020; Vella et al., 2020). Moreover, both ERα and syndecan-4 are able to bind to IGF-1Rβ (Song et al., 2004; Afratis et al., 2017). Thus, a potential ERα-IGF-1Rβ-syndecan-4 interplay in the heart is a compelling possibility. The role of syndecan-4 in estrogen-mediated ERα-PI3K-Akt signaling warrants further study.

Downstream functional outcomes of insulin-Akt signaling include the translocation of glucose transporters to the cell membrane, allowing for an influx of glucose into the intracellular space (Szablewski, 2017). In line with others who have found a decrease in GLUT4 expression with chronic Akt activation (Matsui et al., 2006), we also observed a lowered GLUT4 level in sedentary female syndecan-4^−/−^ mice. Others have also found that mice with cardiac-specific deletion of GLUT4 exhibit elevated GLUT1 (Abel et al., 1999). This was apparently not the case in the female syndecan-4^−/−^ heart, as immunoblotting revealed similar GLUT1 levels as in WT hearts. This may be explained by the observed lowering of GLUT4 levels rather than its absence, or the favorable metabolic substrate of the heart being fatty acids under basal conditions (Szablewski, 2017). In the female syndecan-4^−/−^ heart, we also observed an elevation in pSer9-GSK-3β, which is known to increase glycogen storage in the heart (Matsui et al., 2006). Thus, it is possible that female syndecan-4^−/−^ mice have normal glycogen storage in the heart, despite the reduced GLUT4 level. It is worth mentioning that GLUT4^−/−^ mice surprisingly display normal glucose transportation and glycogen synthesis (Stenbit et al., 2000).

Another downstream effector of Akt signaling is eNOS production and phosphorylation, which leads to the subsequent generation of nitric oxide (NO). One of the key functions of eNOS is blood-pressure control, as eNOS^−/−^ mice are hypertensive (Huang et al., 1995). In line with previous observations (Calvert et al., 2011), exercise upregulated the pSer1177-eNOS level in the heart. However, we did not observe any differences in the pSer1177-eNOS levels between sedentary female syndecan-4^−/−^ and WT mice. Interestingly, past studies have reported elevated blood pressure in syndecan-4^−/−^ mice, hypothesized to be a physical attribute of the decreased pSer1179-eNOS level (Partovian et al., 2008). With the assumption that past syndecan-4^−/−^ studies have been performed in male mice, female syndecan-4^−/−^ mice likely have normal blood pressure. Additionally, these data indicate that alterations in eNOS activity in syndecan-4^−/−^ might be sex specific. Indeed, sex differences in Akt-phosphorylation and eNOS activity have been reported in hearts subjected to lipopolysaccharide (LPS) treatment, where WT female mice had a significant increase in pSer473-Akt and pSer1177-eNOS compared to male counterparts (Chen et al., 2014).

Although we clearly detected increased pSer1177-eNOS levels after 2 weeks with treadmill running, the syndecan-4 mRNA level was unaltered in the heart of WT mice. Previous studies in mice have found elevated levels of syndecan-4 after 4 weeks of swimming induced hypertrophy (Xie et al., 2016). An increase in syndecan-4 mRNA transcript levels and blood serum concentrations have also been observed in men following acute exercise compared to sedentary men (Hjorth et al., 2015). Others have found that the upregulation in syndecan-4 serum concentrations after exercise decreased back to baseline 2 h post-exercise (Lee et al., 2019). In our study, the unchanged syndecan-4 level is somewhat perplexing, but may be due to the type and length of the exercise period (2 weeks) employed in our study. We found no changes in syndecan-2 in response to exercise, but the mRNA levels of syndecan-1, syndecan-3 and collagens were reduced. In contrast, during cardiac remodeling and fibrosis development the expression levels of syndecan-1, syndecan-3, syndecan-4 and collagens are reported to be increased (Christensen et al., 2019).

Our finding of elevated pSer473-Akt signaling in the female syndecan-4^−/−^ heart is consistent with our previous findings in syndecan-4^−/−^ skeletal muscles (Rønning et al., 2020). Consistent with the skeletal muscle of female syndecan-4^−/−^ mice, where smaller muscle fibers were identified (Rønning et al., 2020), we also identified a reduction in cardiomyocyte size in the female syndecan-4^−/−^ heart. However, this was not complemented by a decrease in heart weight. The reduced cell size in the female syndecan-4^−/−^, but an unaltered heart weight, may be due to an increase in cardiomyocyte number, or differences in ECM components that we did not detect with immunoblotting. However, as expected, differences between the two tissues were also observed. In the female syndecan-4^−/−^ heart, the elevated levels of pSer473-Akt and its downstream target pSer9-GSK-3β (Case et al., 2011) returned to WT levels after exercise. The female syndecan-4^−/−^ heart also had a lowered pThr308-Akt/Akt level, an apparently unaltered mTORc1 activity and an increased LRP6 level after exercise. In contrast to skeletal muscle, no alterations in the Notch/HES-1 pathway, syndecan-2, or in the ECM components decorin, fibromodulin, biglycan, LOX, collagen 1a2 and 3a1 were observed in the female syndecan-4^−/−^ heart. The differences between skeletal muscle and heart muscle are likely explained by different physiological roles and plasticity. The fundamental role of the skeletal muscle is defined by its ability to adapt to stimuli such as exercise and nutritional availability. The heart, though also able to compensate to physiological stimuli such as exercise and pregnancy *via* hypertrophy, exhibits more limited plasticity before pathological remodeling takes place (Hill and Olson, 2008).

Finally, as we have shown in our study, sex differences exist in the syndecan-4^−/−^ mice. Initially, upon their creation, both sexes of syndecan-4^−/−^ mice were reported to be viable, reproduce normally, and to have normal phenotypes (Ishiguro et al., 2000). Since then, however, wound healing studies in female syndecan-4^−/−^ mice have revealed delayed wound closure and healing compared to WT mice (Echtermeyer et al., 2001). Importantly, much of the research conducted to date investigating the effects of syndecan-4 ablation in cardiovascular disease has been performed in male mice (Finsen et al., 2011; Matsui et al., 2011; Li et al., 2017). At baseline, male syndecan-4^−/−^ mice have normal cardiac dimensions and function compared to WT hearts (Finsen et al., 2011). The cardiac structure and function of female syndecan-4^−/−^ mice at baseline are not known. Sex-determined molecular differences are common, especially in regards to metabolic studies. Recent work investigating the effect of high-fat diets in syndecan-4^−/−^ mice have found that female syndecan-4^−/−^ mice gained more weight, and have increased cholesterol, triglycerides, and glucose levels compared to male syndecan-4^−/−^ mice (De Luca et al., 2019). We have here shown that female syndecan-4^−/−^ mice also have an elevated insulin/pSer473-Akt/pSer9-GSK-3β signaling pathway and lowered SCOP, pThr308Akt/Akt and GLUT4 levels. Sex hormones have been suggested to play an important role in insulin sensitivity, and interestingly, low physiological concentrations of insulin has been shown to increase Akt activation in intra-abdominal/perigonadal adipocytes from female, but not male mice (Vital et al., 2006; Macotela et al., 2009). Whereas acute increase in cardiac Akt signaling potentiates physiological adaptation to, for example, exercise, chronic upregulation in Akt may promote pathological growth and remodeling (Nagoshi et al., 2005; Matsui et al., 2006). Thus, whether the altered pSer473-Akt and pThr308Akt levels adversely affect or preferentially protect female syndecan-4^−/−^ mice in a pathological situation remains unclear and stresses the requirement to include both sexes in future investigations.

In conclusion, we have presently identified smaller cardiomyocytes, an elevated insulin/pSer473-Akt/pSer9-GSK-3β signaling pathway, and lowered SCOP, pThr308-Akt/Akt and GLUT4 levels in the female syndecan-4^−/−^ heart. The female syndecan-4^−/−^ heart also exhibited an increased LRP6 level after exercise, but otherwise few alterations were found. In contrast, cardiomyocyte size, and Akt signaling in the cardiac and skeletal muscles from male syndecan-4^−/−^ mice were unaltered, indicating important sex specific differences. How these chronic alterations affect cardiac dimensions and function at baseline and in the failing female syndecan-4^−/−^ heart needs to be investigated in future work.

## Data Availability

The original contributions presented in the study are included in the article/Supplementary Material, further inquiries can be directed to the corresponding author.

## Abbreviations

CaMKIIδ, calcium (Ca2+)/calmodulin (CaM)-dependent protein kinase type II delta; cavin1, caveolae-associated protein 1; c-Raf, Raf proto-oncogene serine/threonine-protein kinase; c-SRC, proto-oncogene tyrosine-protein kinase Src; 4E-BP1, eukaryotic translation initiation factor 4E-binding protein 1; ECM, extracellular matrix; eNOS, endothelial nitric oxide synthase; ERα, estrogen receptor α; ERK1/2, extracellular signal-regulated kinase 1/2; ET, exercise trained; GAG, glycosaminoglycan; GAPDH, glyceraldehyde 3-phosphate dehydrogenase; GLUT4, glucose transporter 4; GSK-3β, glycogen synthase kinase-3 beta; IGF-1R, insulin like growth factor-1 receptor; ILK, integrin-linked protein kinase; INSR, insulin receptor; LRP6, low density ribosomal protein 6; LV, left ventricle; MI, myocardial infarction; MLP, muscle LIM protein; MMP2, 72 kDa type IV collagenase; NCX1, sodium/calcium exchanger 1; PDK1, 3-phosphoinositide-dependent protein kinase 1; PIP2, phosphatidylinositol 4,5-bisphosphate; PKCα, protein kinase C α; PP2A, protein phosphatase 2A; RCAN1.4, regulator of calcineurin 1 isoform 4; RhoA, transforming protein RhoA; RPL32, 60S ribosomal protein L32; RPS6, 40S ribosomal protein S6; RTK, receptor tyrosine kinase; SCOP, suprachiasmatic nucleus circadian oscillatory protein; SED, sedentary; TA, tibialis anterior; TRIM72, tripartite motif-containing protein 72; WGA, wheat germ agglutinin; WT, wild type; ZO-1, tight junction protein Zo-1.

## References

[B1] Abel E. D. Kaulbach H. C. Tian R. Hopkins J. C. A. Duffy J. Doetschman T. (1999). Cardiac hypertrophy with preserved contractile function after selective deletion of GLUT4 from the heart. J. Clin. Invest. 104, 1703–1714. 10.1172/JCI7605 10606624 PMC409881

[B2] Afratis N. A. Bouris P. Skandalis S. S. Multhaupt H. A. Couchman J. R. Theocharis A. D. (2017). IGF-IR cooperates with ERα to inhibit breast cancer cell aggressiveness by regulating the expression and localisation of ECM molecules. Sci. Rep. 7, 40138. 10.1038/srep40138 28079144 PMC5228153

[B3] Altomare D. A. Testa J. R. (2005). Perturbations of the AKT signaling pathway in human cancer. Oncogene 24, 7455–7464. 10.1038/sj.onc.1209085 16288292

[B4] Astudillo P. Carrasco H. Larraín J. (2014). Syndecan-4 inhibits Wnt/β-catenin signaling through regulation of low-density-lipoprotein receptor-related protein (LRP6) and R-spondin 3. Int. J. Biochem. Cell Biol. 46, 103–112. 10.1016/j.biocel.2013.11.012 24275095

[B5] Beg M. Srivastava A. Shankar K. Varshney S. Rajan S. Gupta A. (2016). PPP2R5B, a regulatory subunit of PP2A, contributes to adipocyte insulin resistance. Mol. Cell. Endocrinol. 437, 97–107. 10.1016/j.mce.2016.08.016 27521959

[B6] Brahmkhatri V. P. Prasanna C. Atreya H. S. (2015). Insulin-like growth factor system in cancer: Novel targeted therapies. Biomed. Res. Int. 2015, 538019. 10.1155/2015/538019 25866791 PMC4383470

[B7] Calvert J. W. Condit M. E. Aragón J. P. Nicholson C. K. Moody B. F. Hood R. L. (2011). Exercise protects against myocardial ischemia-reperfusion injury via stimulation of β_3_-adrenergic receptors and increased nitric oxide signaling: Role of nitrite and nitrosothiols. Circ. Res. 108, 1448–1458. 10.1161/CIRCRESAHA.111.241117 21527738 PMC3140870

[B8] Case N. Thomas J. Sen B. Styner M. Xie Z. Galior K. (2011). Mechanical regulation of glycogen synthase kinase 3β (GSK3β) in mesenchymal stem cells is dependent on Akt protein serine 473 phosphorylation via mTORC2 protein. J. Biol. Chem. 286, 39450–39456. 10.1074/jbc.M111.265330 21956113 PMC3234768

[B9] Chen J. Chiazza F. Collino M. Patel N. S. A. Coldewey S. M. Thiemermann C. (2014). Gender dimorphism of the cardiac dysfunction in murine sepsis: Signalling mechanisms and age-dependency. PLoS One 9, e100631. 10.1371/journal.pone.0100631 24945834 PMC4063956

[B10] Christensen G. Herum K. M. Lunde I. G. (2019). Sweet, yet underappreciated: Proteoglycans and extracellular matrix remodeling in heart disease. Matrix Biol. 75-76, 286–299. 10.1016/j.matbio.2018.01.001 29337052

[B11] Condorelli G. Drusco A. Stassi G. Bellacosa A. Roncarati R. Iaccarino G. (2002). Akt induces enhanced myocardial contractility and cell size in vivo in transgenic mice. Proc. Natl. Acad. Sci. U. S. A. 99, 12333–12338. 10.1073/pnas.172376399 12237475 PMC129445

[B12] Cooper IV G. (1987). Cardiocyte adaptation to chronically altered load. Annu. Rev. Physiol. 49, 501–518. 10.1146/annurev.ph.49.030187.002441 2952050

[B13] Couchman J. R. Gopal S. Lim H. C. Nørgaard S. Multhaupt H. A. B. (2015). Fell-muir lecture: Syndecans: From peripheral coreceptors to mainstream regulators of cell behaviour. Int. J. Exp. Pathol. 96, 1–10. 10.1111/iep.12112 25546317 PMC4352346

[B14] Dao V. T.-V. Floeren M. Kumpf S. Both C. Peter B. Balz V. (2011). Catalase activity prevents exercise-induced up-regulation of vasoprotective proteins in venous tissue. J. Cell. Mol. Med. 15, 2326–2334. 10.1111/j.1582-4934.2010.01227.x 21129156 PMC3822944

[B15] De Luca M. Vecchie D. Athmanathan B. Gopalkrishna S. Valcin J. A. Swain T. M. (2019). Genetic deletion of syndecan-4 alters body composition, metabolic phenotypes, and the function of metabolic tissues in female mice fed a high-fat diet. Nutrients 11, E2810. 10.3390/nu11112810 31752080 PMC6893658

[B16] DeBosch B. Treskov I. Lupu T. S. Weinheimer C. Kovacs A. Courtois M. (2006). Akt1 is required for physiological cardiac growth. Circulation 113, 2097–2104. 10.1161/CIRCULATIONAHA.105.595231 16636172

[B17] Delcommenne M. Tan C. Gray V. Rue L. Woodgett J. Dedhar S. (1998). Phosphoinositide-3-OH kinase-dependent regulation of glycogen synthase kinase 3 and protein kinase B/AKT by the integrin-linked kinase. Proc. Natl. Acad. Sci. U. S. A. 95, 11211–11216. 10.1073/pnas.95.19.11211 9736715 PMC21621

[B18] Echtermeyer F. Streit M. Wilcox-Adelman S. Saoncella S. Denhez F. Detmar M. (2001). Delayed wound repair and impaired angiogenesis in mice lacking syndecan-4. J. Clin. Invest. 107, R9–R14. 10.1172/JCI10559 11160142 PMC199172

[B19] Elfenbein A. Simons M. (2013). Syndecan-4 signaling at a glance. J. Cell Sci. 126, 3799–3804. 10.1242/jcs.124636 23970415 PMC3757327

[B20] Fischer Y. Thomas J. Sevilla L. Muñoz P. Becker C. Holman G. (1997). Insulin-induced recruitment of glucose transporter 4 (GLUT4) and GLUT1 in isolated rat cardiac myocytes: Evidence of the existence of different intracellular GLUT4 vesicle populations. J. Biol. Chem. 272, 7085–7092. 10.1074/jbc.272.11.7085 9054401

[B21] Finsen A. V. Lunde I. G. Sjaastad I. Ostli E. K. Lyngra M. Jarstadmarken H. O. (2011). Syndecan-4 is essential for development of concentric myocardial hypertrophy via stretch-induced activation of the calcineurin-NFAT pathway. PLoS One 6, e28302. 10.1371/journal.pone.0028302 22164265 PMC3229559

[B22] Fulton D. Gratton J.-P. Mccabe T. J. Fontana J. Fujio Y. Walsh K. (1999). Regulation of endothelium-derived nitric oxide production by the protein kinase Akt. Nature 399, 597–601. 10.1038/21218 10376602 PMC3637917

[B23] Gopal S. Søgaard P. Multhaupt H. A. B. Pataki C. Okina E. Xian X. (2015). Transmembrane proteoglycans control stretch-activated channels to set cytosolic calcium levels. J. Cell Biol. 210, 1199–1211. 10.1083/jcb.201501060 26391658 PMC4586746

[B24] Herum K. M. Lunde I. G. Skrbic B. Florholmen G. Behmen D. Sjaastad I. (2013). Syndecan-4 signaling via NFAT regulates extracellular matrix production and cardiac myofibroblast differentiation in response to mechanical stress. J. Mol. Cell. Cardiol. 54, 73–81. 10.1016/j.yjmcc.2012.11.006 23178899

[B25] Herum K. M. Romaine A. Wang A. Melleby A. O. Strand M. E. Pacheco J. (2020). Syndecan-4 protects the heart from the profibrotic effects of thrombin cleaved osteopontin. J. Am. Heart Assoc. 9, e013518. 10.1161/JAHA.119.013518 32000579 PMC7033859

[B26] Hill J. A. Olson E. N. (2008). Cardiac plasticity. N. Engl. J. Med. 358, 1370–1380. 10.1056/NEJMra072139 18367740

[B27] Hjorth M. Norheim F. Meen A. J. Pourteymour S. Lee S. Holen T. (2015). The effect of acute and long-term physical activity on extracellular matrix and serglycin in human skeletal muscle. Physiol. Rep. 3, e12473. 10.14814/phy2.12473 26290530 PMC4562559

[B28] Horowitz A. Murakami M. Gao Y. Simons M. (1999). Phosphatidylinositol-4, 5-bisphosphate mediates the interaction of syndecan-4 with protein kinase C. Biochemistry 38, 15871–15877. 10.1021/bi991363i 10625452

[B29] Huang P. L. Huang Z. Mashimo H. Bloch K. D. Moskowitz M. A. Bevan J. A. (1995). Hypertension in mice lacking the gene for endothelial nitric oxide synthase. Nature 377, 239–242. 10.1038/377239a0 7545787

[B30] Ishiguro K. Kadomatsu K. Kojima T. Muramatsu H. Tsuzuki S. Nakamura E. (2000). Syndecan-4 deficiency impairs focal adhesion formation only under restricted conditions. J. Biol. Chem. 275, 5249–5252. 10.1074/jbc.275.8.5249 10681494

[B31] Jang E. Albadawi H. Watkins M. T. Edelman E. R. Baker A. B. (2012). Syndecan-4 proteoliposomes enhance fibroblast growth factor-2 (FGF-2)-induced proliferation, migration, and neovascularization of ischemic muscle. Proc. Natl. Acad. Sci. U. S. A. 109, 1679–1684. 10.1073/pnas.1117885109 22307630 PMC3277125

[B32] Jhanwar-Uniyal M. Wainwright J. V. Mohan A. L. Tobias M. E. Murali R. Gandhi C. D. (2019). Diverse signaling mechanisms of mTOR complexes: mTORC1 and mTORC2 in forming a formidable relationship. Adv. Biol. Regul. 72, 51–62. 10.1016/j.jbior.2019.03.003 31010692

[B33] Jones A. G. Hattersley A. T. (2013). The clinical utility of C-peptide measurement in the care of patients with diabetes. Diabet. Med. 30, 803–817. 10.1111/dme.12159 23413806 PMC3748788

[B34] Ju R. Simons M. (2013). Syndecan 4 regulation of PDK1-dependent Akt activation. Cell. Signal. 25, 101–105. 10.1016/j.cellsig.2012.09.007 22975683 PMC3508137

[B35] Kleinert M. Parker B. L. Fritzen A. M. Knudsen J. R. Jensen T. E. Kjøbsted R. (2017). Mammalian target of rapamycin complex 2 regulates muscle glucose uptake during exercise in mice. J. Physiol. 595, 4845–4855. 10.1113/JP274203 28464351 PMC5509878

[B36] Lai K.-M. V. Gonzalez M. Poueymirou W. T. Kline W. O. Na E. Zlotchenko E. (2004). Conditional activation of akt in adult skeletal muscle induces rapid hypertrophy. Mol. Cell. Biol. 24, 9295–9304. 10.1128/MCB.24.21.9295-9304.2004 15485899 PMC522257

[B37] Lee D. Oh E.-S. Woods A. Couchman J. R. Lee W. (1998). Solution structure of a syndecan-4 cytoplasmic domain and its interaction with phosphatidylinositol 4, 5-bisphosphate. J. Biol. Chem. 273, 13022–13029. 10.1074/jbc.273.21.13022 9582338

[B38] Lee S. Kolset S. O. Birkeland K. I. Drevon C. A. Reine T. M. (2019). Acute exercise increases syndecan-1 and -4 serum concentrations. Glycoconj. J. 36, 113–125. 10.1007/s10719-019-09869-z 30949875

[B39] Li G. Xie J. Chen J. Li R. Wu H. Zhang X. (2017). Syndecan-4 deficiency accelerates the transition from compensated hypertrophy to heart failure following pressure overload. Cardiovasc. Pathol. 28, 74–79. 10.1016/j.carpath.2017.03.008 28395201

[B40] Li X. Stevens P. D. Liu J. Yang H. Wang W. Wang C. (2014). PHLPP is a negative regulator of RAF1, which reduces colorectal cancer cell motility and prevents tumor progression in mice. Gastroenterology 146, 13011301–13011312. 10.1053/j.gastro.2014.02.003 PMC3992173 24530606

[B41] Lim S.-T. Longley R. L. Couchman J. R. Woods A. (2003). Direct binding of syndecan-4 cytoplasmic domain to the catalytic domain of protein kinase C alpha (PKC alpha) increases focal adhesion localization of PKC alpha. J. Biol. Chem. 278, 13795–13802. 10.1074/jbc.M208300200 12571249

[B42] Liu Y. Neogi A. Mani A. (2020). The role of Wnt signalling in development of coronary artery disease and its risk factors. Open Biol. 10, 200128. 10.1098/rsob.200128 33081636 PMC7653355

[B43] Macotela Y. Boucher J. Tran T. T. Kahn C. R. (2009). Sex and depot differences in adipocyte insulin sensitivity and glucose metabolism. Diabetes 58, 803–812. 10.2337/db08-1054 19136652 PMC2661589

[B44] Manning B. D. Toker A. (2017). AKT/PKB signaling: Navigating the network. Cell 169, 381–405. 10.1016/j.cell.2017.04.001 28431241 PMC5546324

[B45] Mathiesen S. B. Lunde M. Aronsen J. M. Romaine A. Kaupang A. Martinsen M. (2019). The cardiac syndecan-4 interactome reveals a role for syndecan-4 in nuclear translocation of muscle LIM protein (MLP). J. Biol. Chem. 294, 8717–8731. 10.1074/jbc.RA118.006423 30967474 PMC6552415

[B46] Matsui T. Nagoshi T. Hong E.-G. Luptak I. Hartil K. Li L. (2006). Effects of chronic Akt activation on glucose uptake in the heart. Am. J. Physiol. Endocrinol. Metab. 290, E789–E797. 10.1152/ajpendo.00564.2004 16352665

[B47] Matsui Y. Ikesue M. Danzaki K. Morimoto J. Sato M. Tanaka S. (2011). Syndecan-4 prevents cardiac rupture and dysfunction after myocardial infarction. Circ. Res. 108, 1328–1339. 10.1161/CIRCRESAHA.110.235689 21493899

[B48] Miyamoto S. Purcell N. H. Smith J. M. Gao T. Whittaker R. Huang K. (2010). PHLPP-1 negatively regulates Akt activity and survival in the heart. Circ. Res. 107, 476–484. 10.1161/circresaha.109.215020 20576936 PMC2957297

[B49] Moc C. Taylor A. E. Chesini G. P. Zambrano C. M. Barlow M. S. Zhang X. (2015). Physiological activation of Akt by PHLPP1 deletion protects against pathological hypertrophy. Cardiovasc. Res. 105, 160–170. 10.1093/cvr/cvu243 25411382 PMC4303795

[B50] Motallebnezhad M. Aghebati-Maleki L. Jadidi-Niaragh F. Nickho H. Samadi-Kafil H. Shamsasenjan K. (2016). The insulin-like growth factor-I receptor (IGF-IR) in breast cancer: Biology and treatment strategies. Tumour Biol. 37, 11711–11721. 10.1007/s13277-016-5176-x 27444280

[B51] Nagoshi T. Matsui T. Aoyama T. Leri A. Anversa P. Li L. (2005). PI3K rescues the detrimental effects of chronic Akt activation in the heart during ischemia/reperfusion injury. J. Clin. Invest. 115, 2128–2138. 10.1172/JCI23073 16007268 PMC1172227

[B52] Oh E.-S. Woods A. Couchman J. R. (1997). Multimerization of the cytoplasmic domain of syndecan-4 is required for its ability to activate protein kinase C. J. Biol. Chem. 272, 11805–11811. 10.1074/jbc.272.18.11805 9115237

[B53] Partovian C. Ju R. Zhuang Z. W. Martin K. A. Simons M. (2008). Syndecan-4 regulates subcellular localization of mTOR Complex2 and Akt activation in a PKCalpha-dependent manner in endothelial cells. Mol. Cell 32, 140–149. 10.1016/j.molcel.2008.09.010 18851840 PMC2578831

[B54] Rodgers J. T. Vogel R. O. Puigserver P. (2011). Clk2 and B56β mediate insulin-regulated assembly of the PP2A phosphatase holoenzyme complex on Akt. Mol. Cell 41, 471–479. 10.1016/j.molcel.2011.02.007 21329884 PMC3060660

[B55] Romaine A. Melleby A. O. Alam J. Lobert V. H. Lu N. Lockwood F. E. (2022). Integrin α11β1 and syndecan-4 dual receptor ablation attenuate cardiac hypertrophy in the pressure overloaded heart. Am. J. Physiol. Heart Circ. Physiol. 322, H1057–h1071. 10.1152/ajpheart.00635.2021 35522553

[B56] Rønning S. B. Carlson C. R. Aronsen J. M. Pisconti A. Høst V. Lunde M. (2020). Syndecan-4–/– mice have smaller muscle fibers, increased akt/mTOR/S6K1 and notch/HES-1 pathways, and alterations in extracellular matrix components. Front. Cell Dev. Biol. 8, 730. 10.3389/fcell.2020.00730 32850844 PMC7411008

[B57] Ruvinsky I. Sharon N. Lerer T. Cohen H. Stolovich-Rain M. Nir T. (2005). Ribosomal protein S6 phosphorylation is a determinant of cell size and glucose homeostasis. Genes Dev. 19, 2199–2211. 10.1101/gad.351605 16166381 PMC1221890

[B58] Saoncella S. Echtermeyer F. Denhez F. Nowlen J. K. Mosher D. F. Robinson S. D. (1999). Syndecan-4 signals cooperatively with integrins in a Rho-dependent manner in the assembly of focal adhesions and actin stress fibers. Proc. Natl. Acad. Sci. U. S. A. 96, 2805–2810. 10.1073/pnas.96.6.2805 10077592 PMC15850

[B59] Sargeant R. J. Pâquet M. R. (1993). Effect of insulin on the rates of synthesis and degradation of GLUT1 and GLUT4 glucose transporters in 3T3-L1 adipocytes. Biochem. J. 290, 913–919. 10.1042/bj2900913 8457217 PMC1132367

[B60] Selvetella G. Hirsch E. Notte A. Tarone G. Lembo G. (2004). Adaptive and maladaptive hypertrophic pathways: Points of convergence and divergence. Cardiovasc. Res. 63, 373–380. 10.1016/j.cardiores.2004.04.031 15276462

[B61] Shao D. Tian R. (2015). Glucose transporters in cardiac metabolism and hypertrophy. Compr. Physiol. 6, 331–351. 10.1002/cphy.c150016 26756635 PMC4760112

[B62] Shiojima I. Schiekofer S. Schneider J. G. Belisle K. Sato K. Andrassy M. (2012). Short-term akt activation in cardiac muscle cells improves contractile function in failing hearts. Am. J. Pathol. 181, 1969–1976. 10.1016/j.ajpath.2012.08.020 23031259 PMC3509766

[B63] Solbu M. D. Kolset S. O. Jenssen T. G. Wilsgaard T. Løchen M.-L. Mathiesen E. B. (2018). Gender differences in the association of syndecan-4 with myocardial infarction: The population-based Tromsø Study. Atherosclerosis 278, 166–173. 10.1016/j.atherosclerosis.2018.08.005 30278359

[B64] Song R. X. Barnes C. J. Zhang Z. Bao Y. Kumar R. Santen R. J. (2004). The role of Shc and insulin-like growth factor 1 receptor in mediating the translocation of estrogen receptor α to the plasma membrane. Proc. Natl. Acad. Sci. U. S. A. 101, 2076–2081. 10.1073/pnas.0308334100 14764897 PMC357054

[B65] Srinivasan M. Begum N. (1994). Regulation of protein phosphatase 1 and 2A activities by insulin during myogenesis in rat skeletal muscle cells in culture. J. Biol. Chem. 269, 12514–12520. 10.1016/S0021-9258(18)99905-9 8175660

[B66] Staal S. P. Hartley J. W. Rowe W. P. (1977). Isolation of transforming murine leukemia viruses from mice with a high incidence of spontaneous lymphoma. Proc. Natl. Acad. Sci. U. S. A. 74, 3065–3067. 10.1073/pnas.74.7.3065 197531 PMC431413

[B67] Stenbit A. E. Katz E. B. Chatham J. C. Geenen D. L. Factor S. M. Weiss R. G. (2000). Preservation of glucose metabolism in hypertrophic GLUT4-null hearts. Am. J. Physiol. Heart Circ. Physiol. 279, H313–H318. 10.1152/ajpheart.2000.279.1.H313 10899071

[B68] Szablewski L. (2017). Glucose transporters in healthy heart and in cardiac disease. Int. J. Cardiol. 230, 70–75. 10.1016/j.ijcard.2016.12.083 28034463

[B69] Tan J. Digicaylioglu M. Wang S. X. J. Dresselhuis J. Dedhar S. Mills J. (2019). Insulin attenuates apoptosis in neuronal cells by an integrin-linked kinase-dependent mechanism. Heliyon 5, e02294. 10.1016/j.heliyon.2019.e02294 31463398 PMC6706370

[B70] Ueda K. Adachi Y. Liu P. Fukuma N. Takimoto E. (2020). Regulatory actions of estrogen receptor signaling in the cardiovascular system. Front. Endocrinol. 10, 909. 10.3389/fendo.2019.00909 PMC6965027 31998238

[B71] VanWinkle W. B. Snuggs M. B. De Hostos E. L. Buja L. M. Woods A. Couchman J. R. (2002). Localization of the transmembrane proteoglycan syndecan-4 and its regulatory kinases in costameres of rat cardiomyocytes: A deconvolution microscopic study. Anat. Rec. 268, 38–46. 10.1002/ar.10130 12209563

[B72] Vella V. De Francesco E. M. Lappano R. Muoio M. G. Manzella L. Maggiolini M. (2020). Microenvironmental determinants of breast cancer metastasis: Focus on the crucial interplay between estrogen and insulin/insulin-like growth factor signaling. Front. Cell Dev. Biol. 810, 608412. 10.3389/fcell.2020.608412 PMC7753049 33364239

[B73] Vital P. Larrieta E. Hiriart M. (2006). Sexual dimorphism in insulin sensitivity and susceptibility to develop diabetes in rats. J. Endocrinol. 190, 425–432. 10.1677/joe.1.06596 16899575

[B74] Walsh K. (2006). Akt signaling and growth of the heart. Circulation 113, 2032–2034. 10.1161/CIRCULATIONAHA.106.615138 16651482

[B75] White D. E. Coutu P. Shi Y.-F. Tardif J.-C. Nattel S. St Arnaud R. (2006). Targeted ablation of ILK from the murine heart results in dilated cardiomyopathy and spontaneous heart failure. Genes Dev. 20, 2355–2360. 10.1101/gad.1458906 16951252 PMC1560410

[B76] Xie J. He G. Chen Q. Sun J. Dai Q. Lu J. (2016). Syndecan-4 signaling is required for exercise-induced cardiac hypertrophy. Mol. Med. 22, 192–201. 10.2119/molmed.2015.00026 26835698 PMC5004706

[B77] Xing Y. Sun W. Wang Y. Gao F. Ma H. (2016). Mutual inhibition of insulin signaling and PHLPP-1 determines cardioprotective efficiency of Akt in aged heart. Aging 8, 873–888. 10.18632/aging.100933 27019292 PMC4931841

[B78] Yang Q. Guan K.-L. (2007). Expanding mTOR signaling. Cell Res. 17, 666–681. 10.1038/cr.2007.64 17680028

[B79] Zaarour N. Berenguer M. Le Marchand-Brustel Y. Govers R. (2012). Deciphering the role of GLUT4 N-glycosylation in adipocyte and muscle cell models. Biochem. J. 445, 265–273. 10.1042/bj20120232 22545627

[B80] Zhang D. Contu R. Latronico M. V. Zhang J. Rizzi R. Catalucci D. (2010). MTORC1 regulates cardiac function and myocyte survival through 4E-BP1 inhibition in mice. J. Clin. Invest. 120, 2805–2816. 10.1172/jci43008 20644257 PMC2912201

[B81] Zhang M. Wang X. Liu M. Liu D. Pan J. Tian J. (2020). Inhibition of PHLPP1 ameliorates cardiac dysfunction via activation of the PI3K/Akt/mTOR signalling pathway in diabetic cardiomyopathy. J. Cell. Mol. Med. 24, 4612–4623. 10.1111/jcmm.15123 32150791 PMC7176843

