# Supplementary material for: The female syndecan-4−/− heart has smaller cardiomyocytes, augmented insulin/pSer473-Akt/pSer9-GSK-3β signaling, and lowered SCOP, pThr308-Akt/Akt and GLUT4 levels
Source: Front Cell Dev Biol. 2022 Aug 25;10:908126. doi: 10.3389/fcell.2022.908126 (PMC9452846; doi:10.3389/fcell.2022.908126)

Full length blots for figure 1A

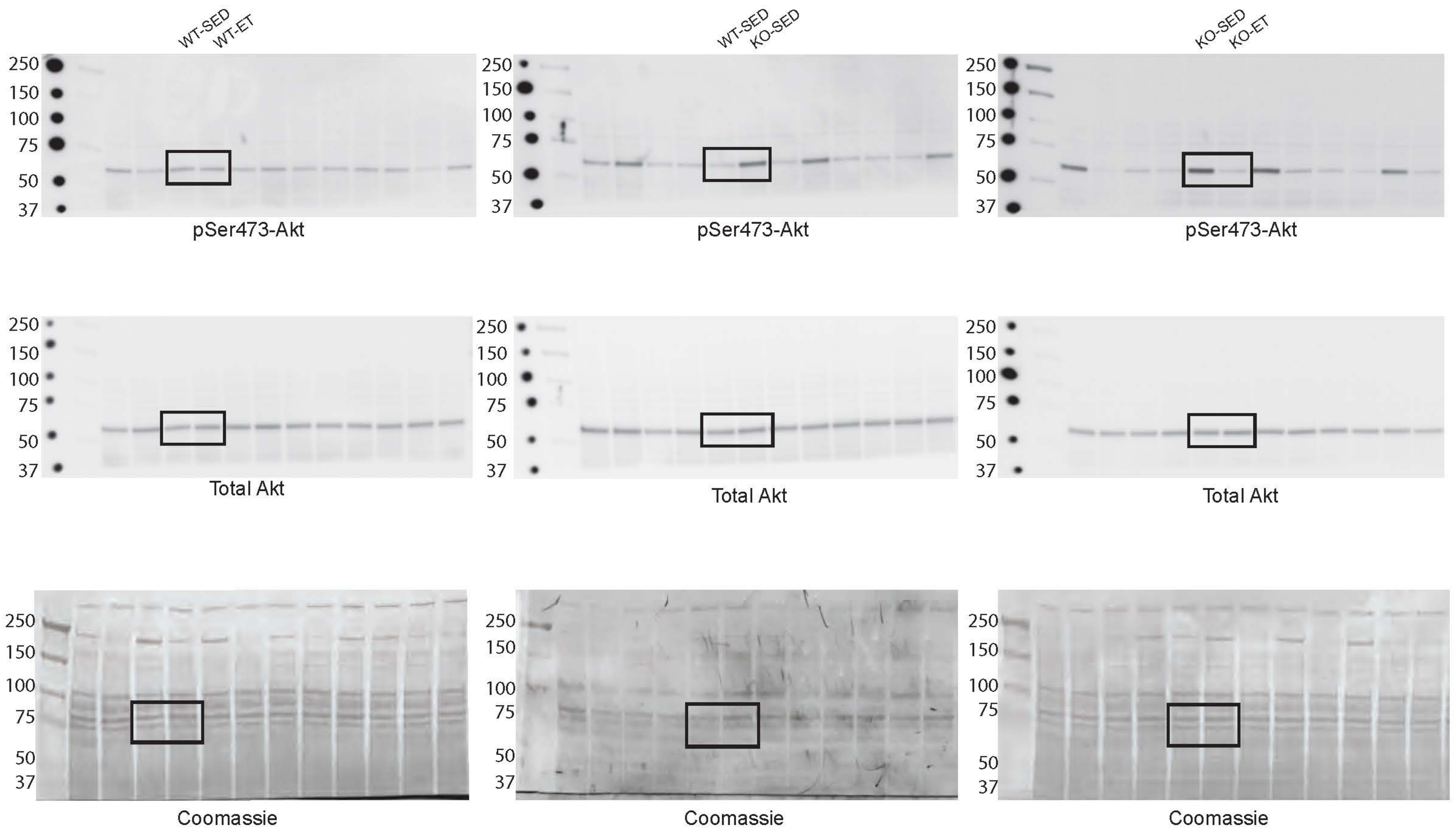

Full length blots for figure 1C

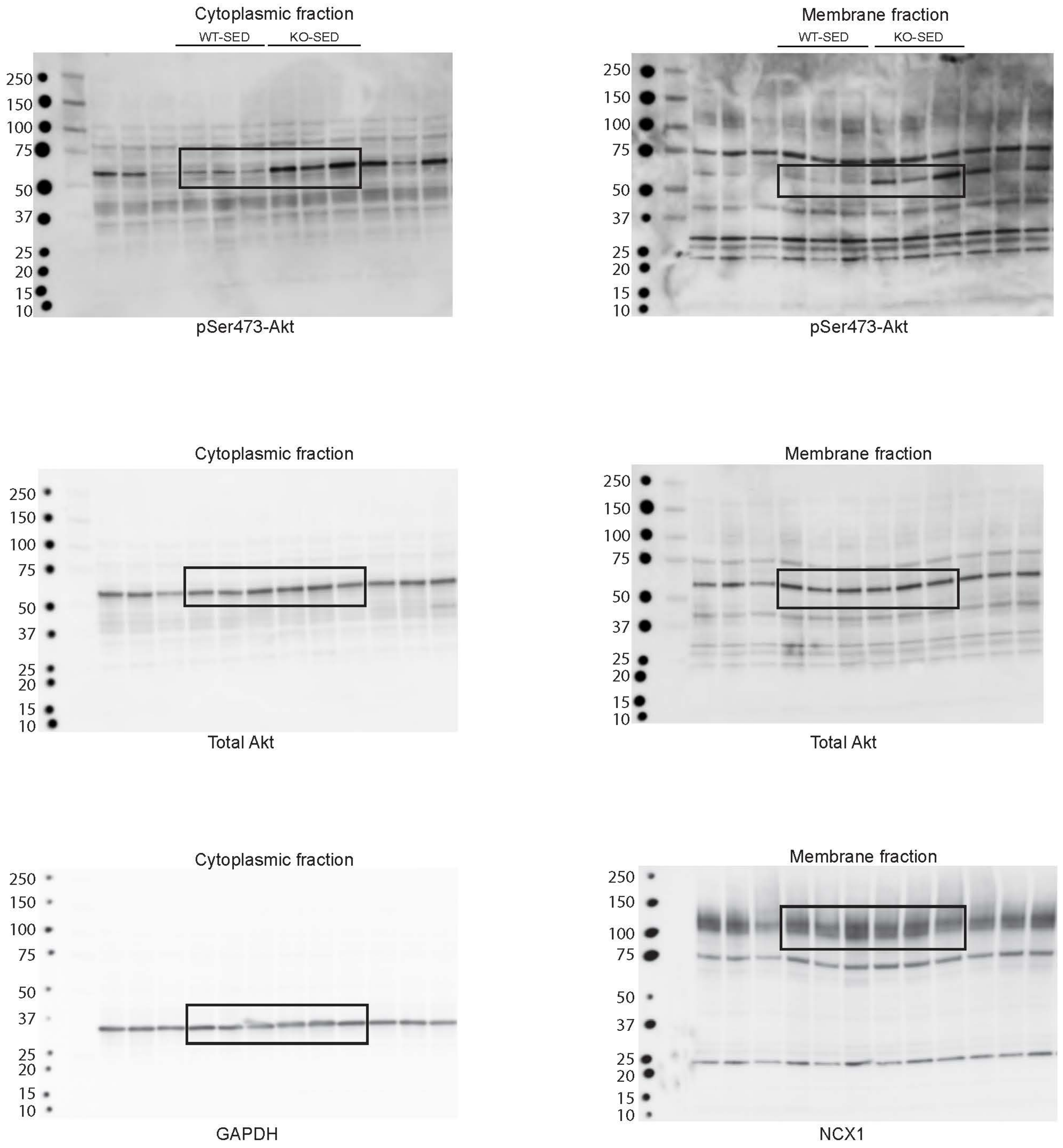

Full length blots for figure 1D

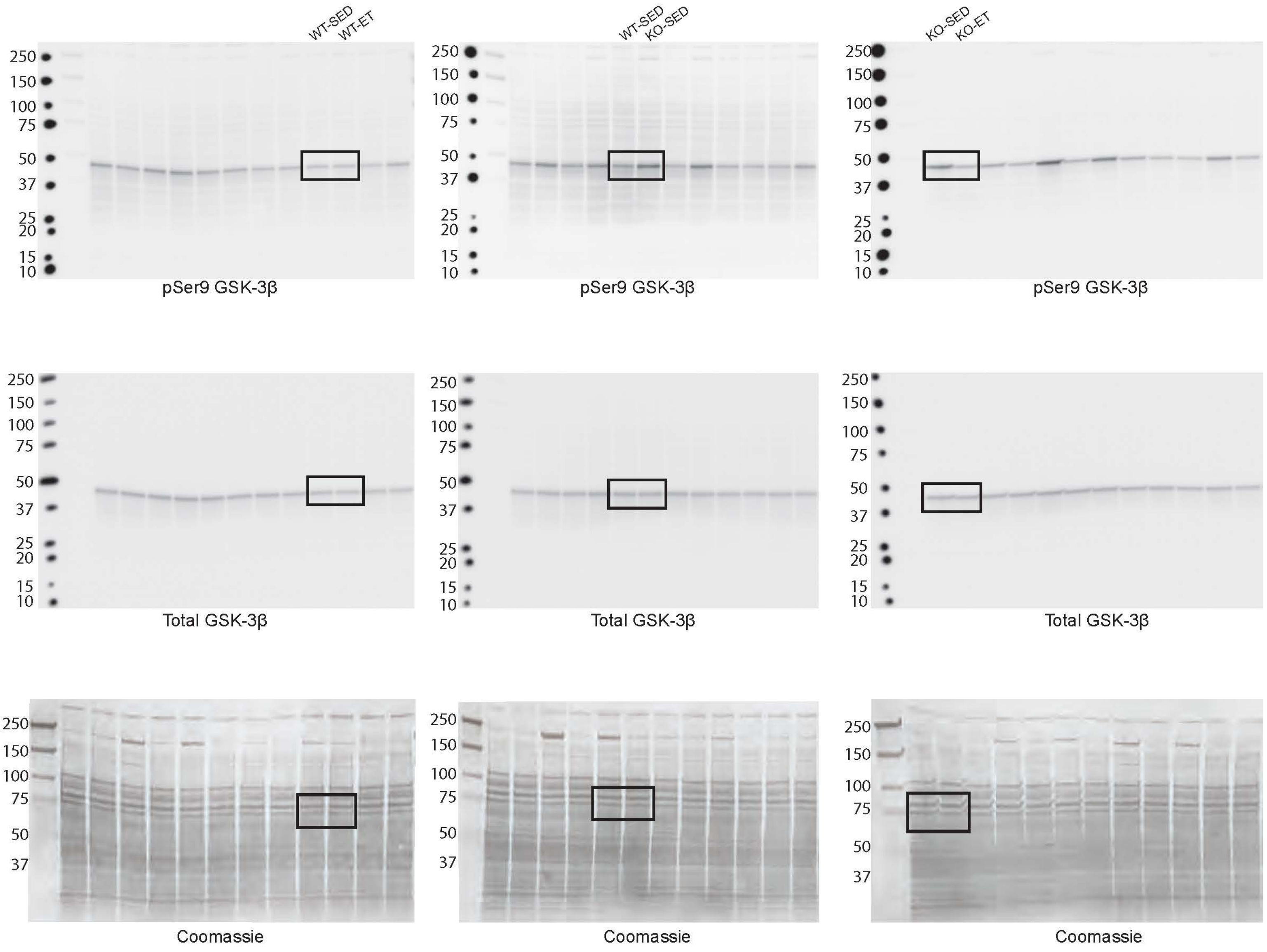

Full length blots for figure 1E

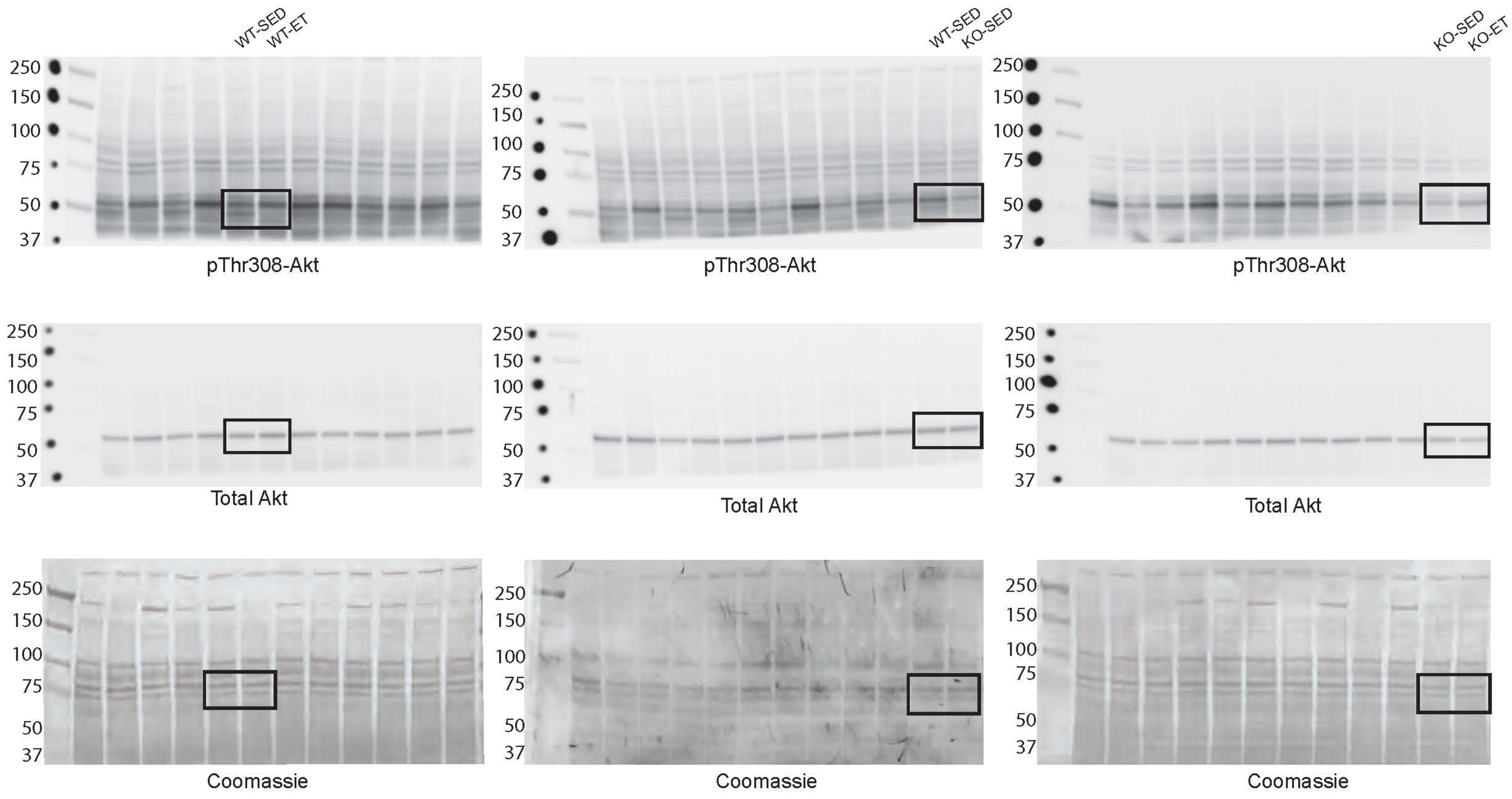

Full length blots for figure 1F

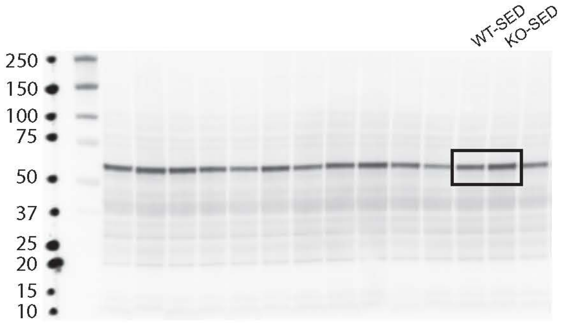

pSer473-Akt

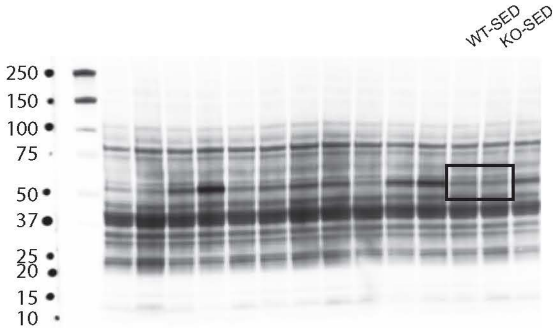

pThr308-Akt

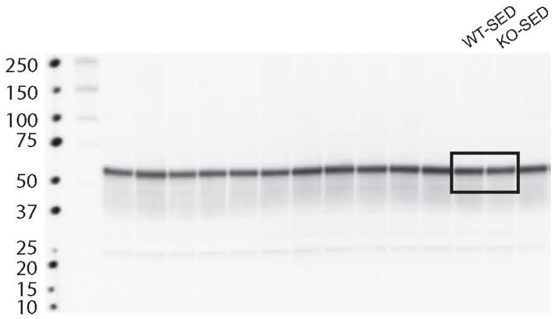

Total Akt

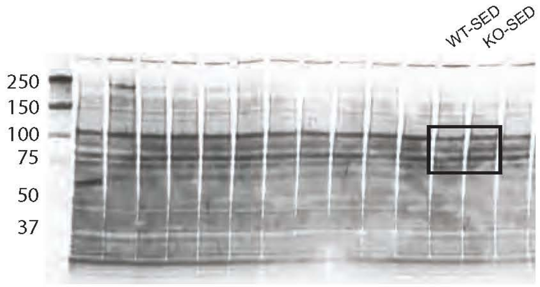

Coomassie

Full length blots for figure 2A

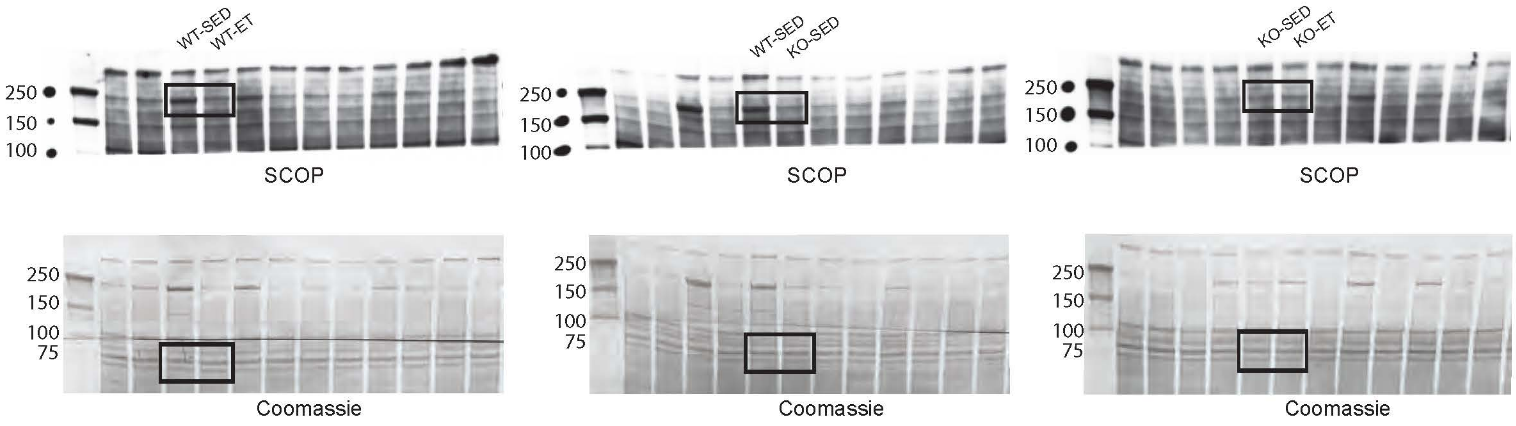

Full length blots for figure 2E

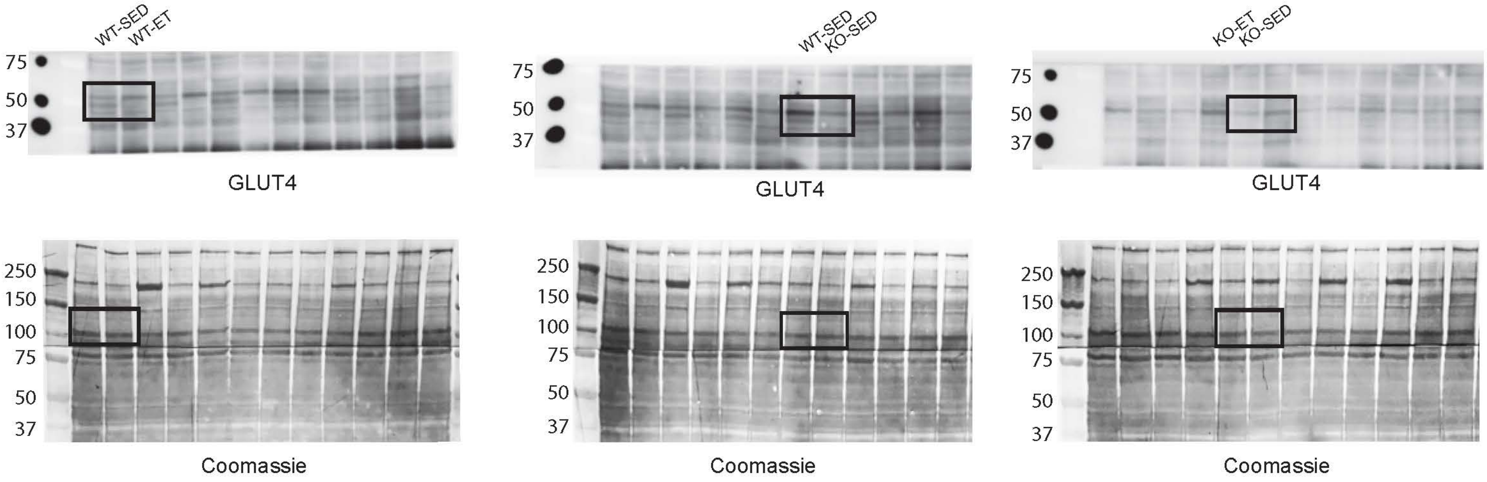

Full length blots for figure 2F

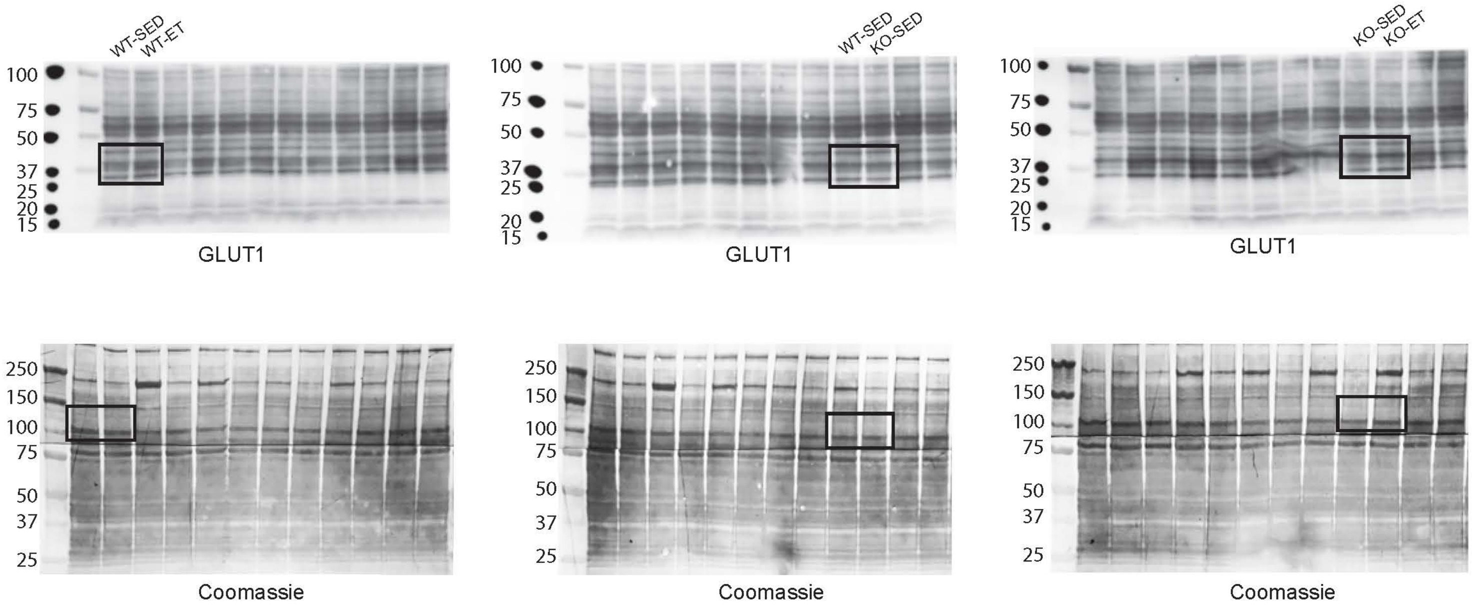

Full length blots for figure 2G

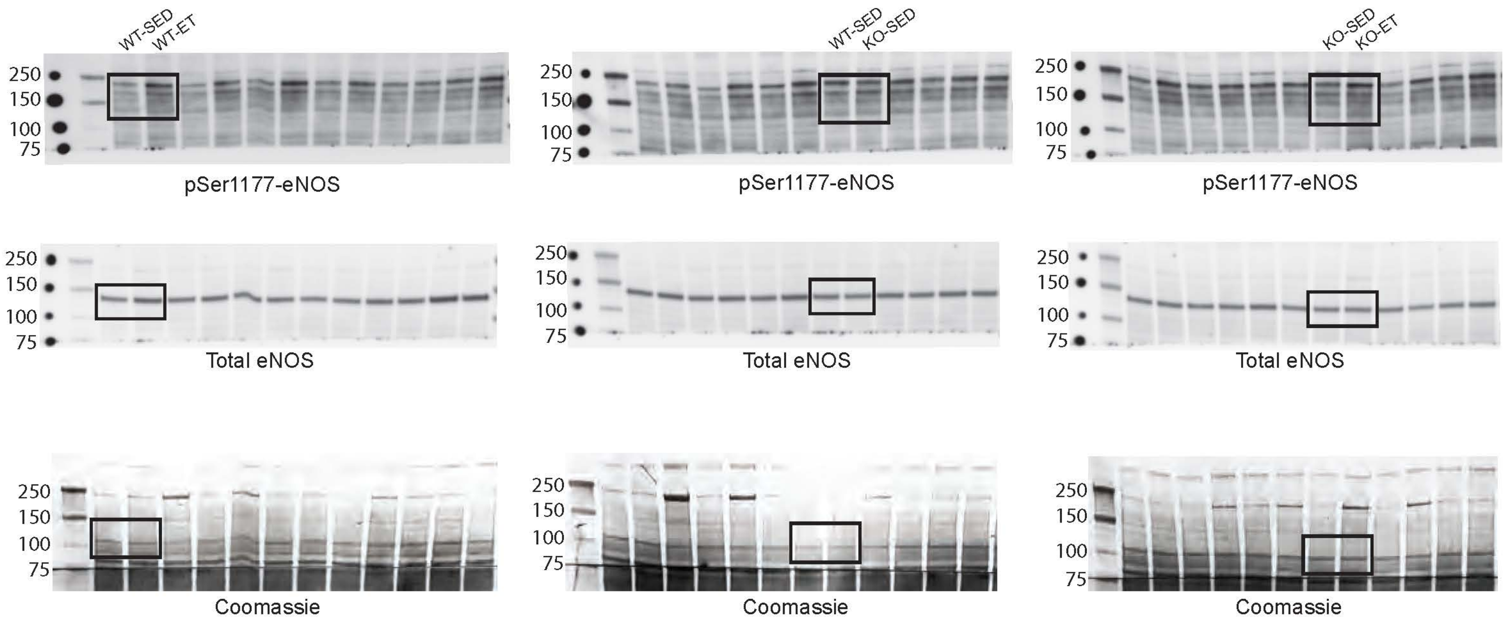

Full length blots for figure 4D

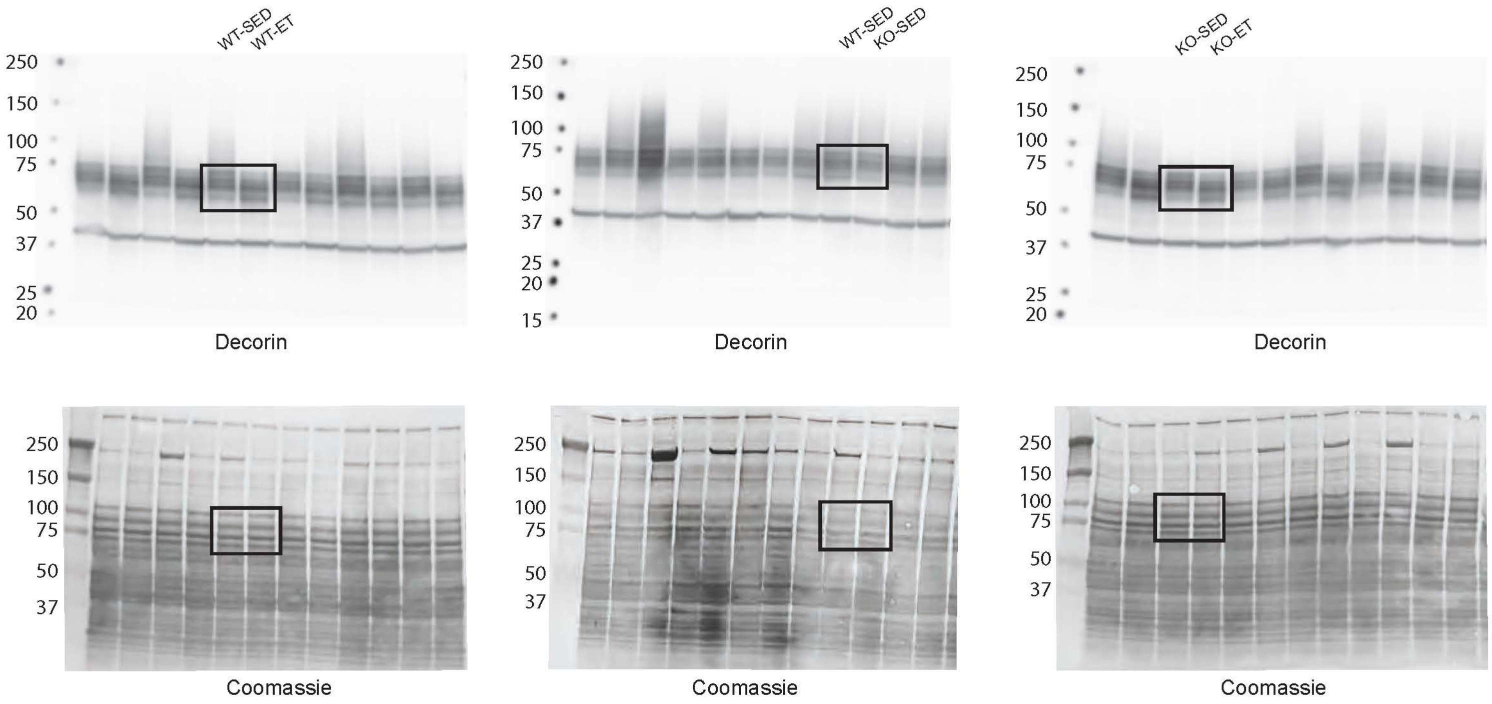

Full length blots for figure 4E

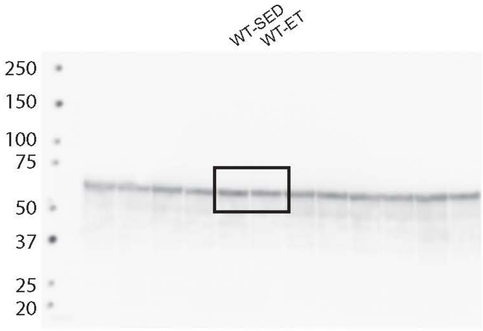

Fibromodulin

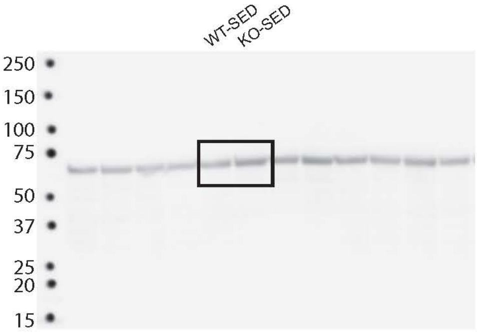

Fibromodulin

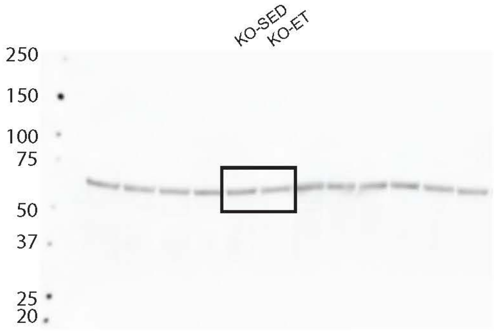

Fibromodulin

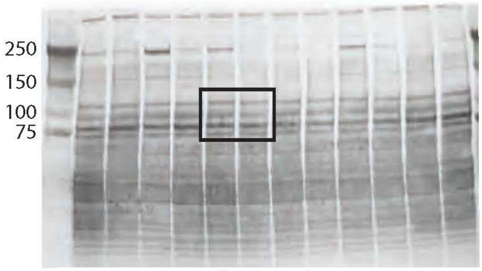

Coomassie

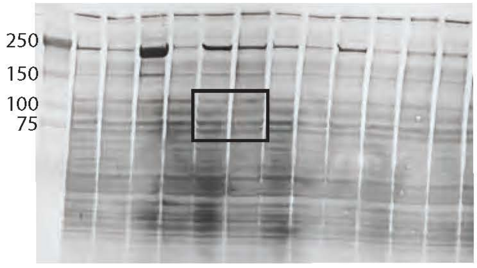

Coomassie

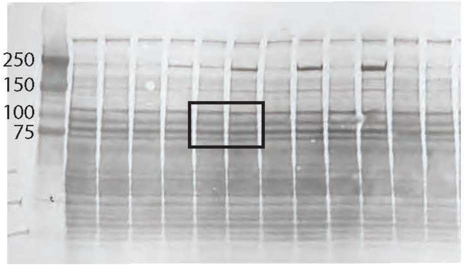

Coomassie

Full length blots for figure 4F

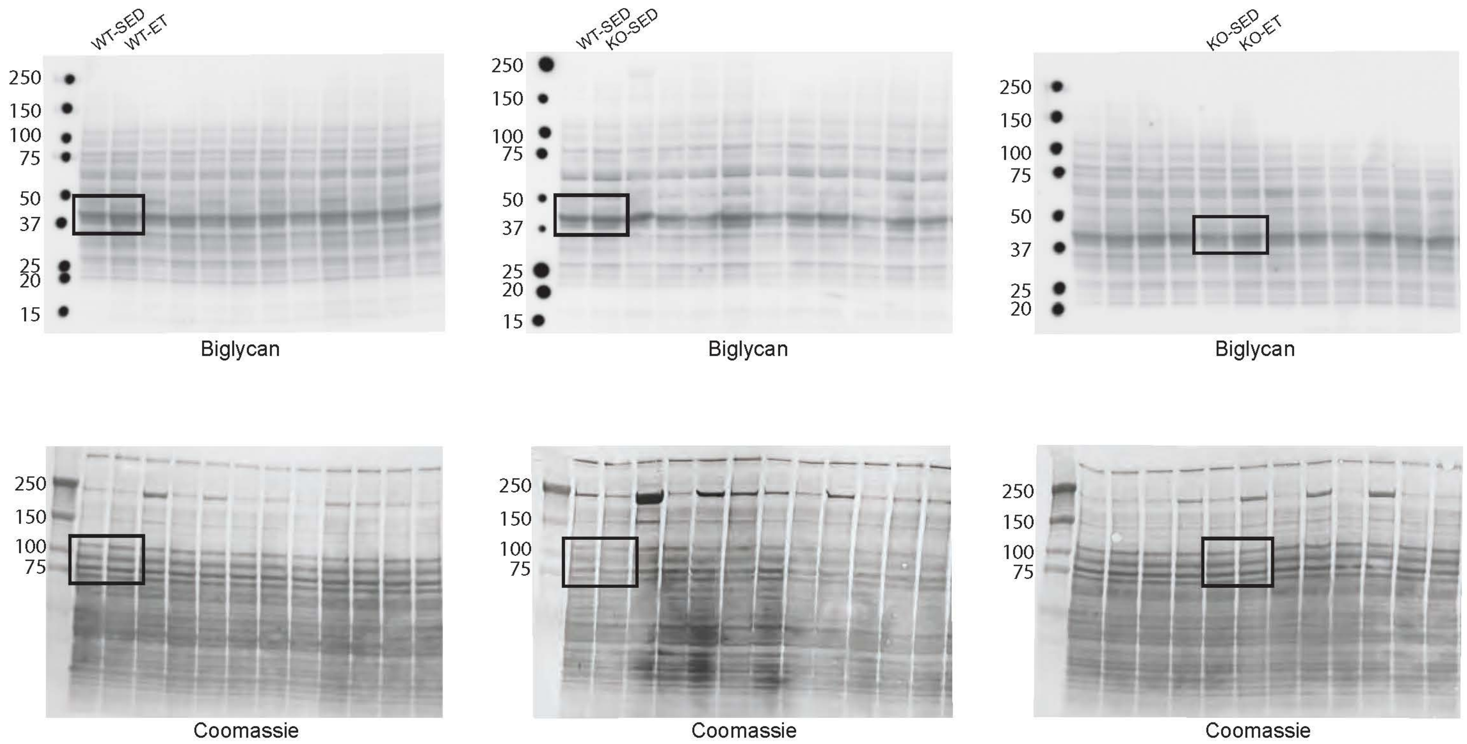

Full length blots for figure 4G

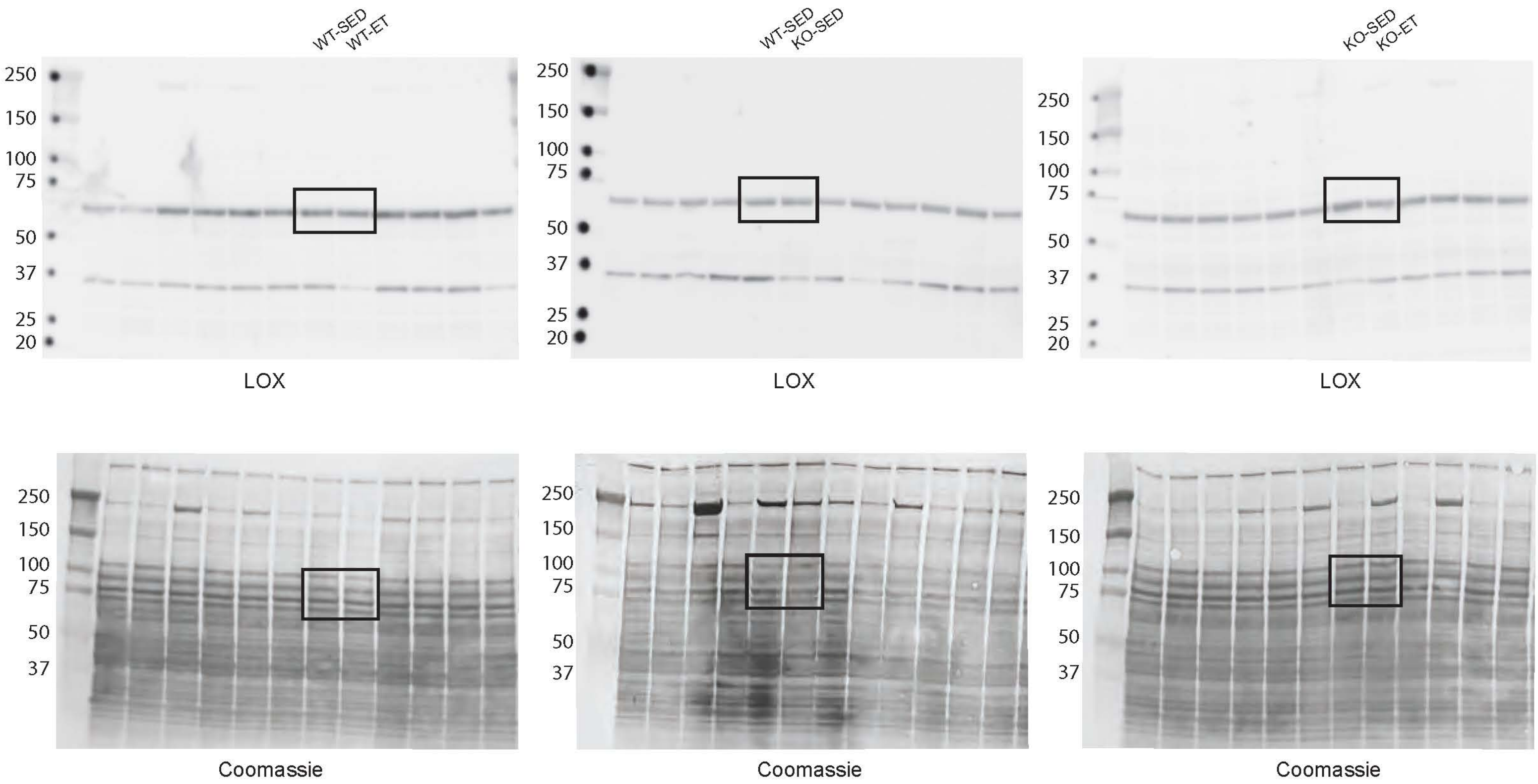

Full length blots for supplementary figure 1A

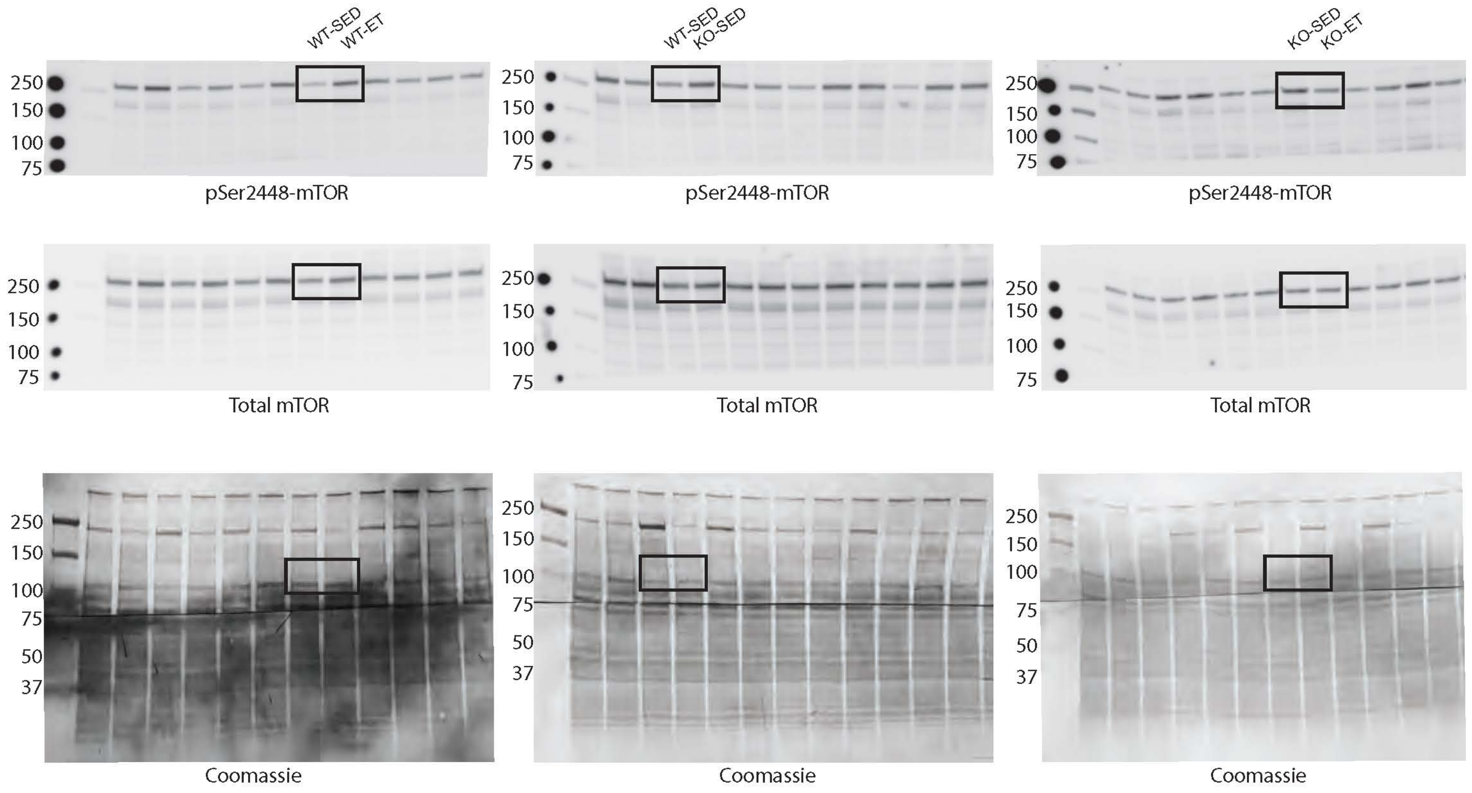

Full length blots for supplementary figure 1B

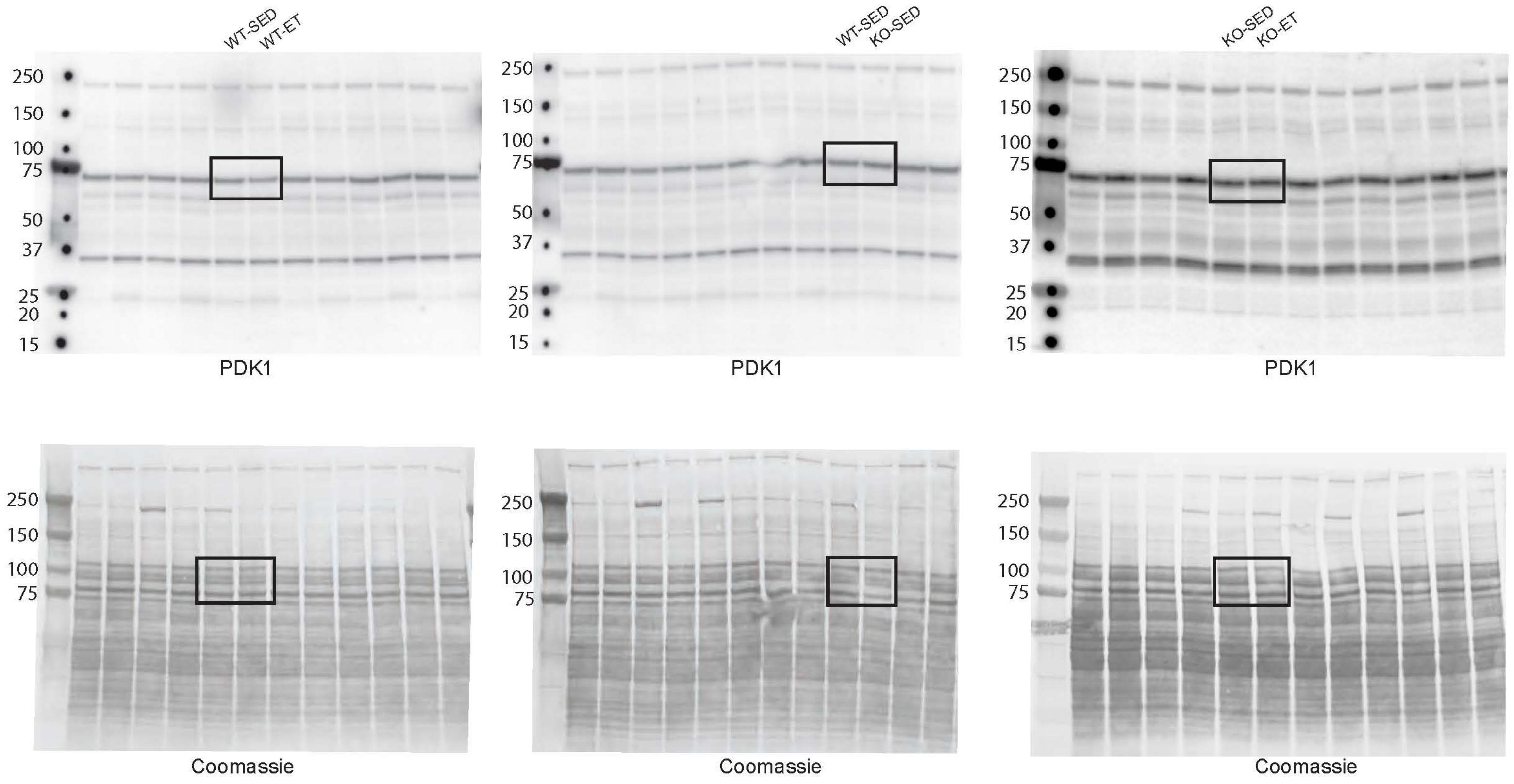

Full length blots for supplementary figure 1C

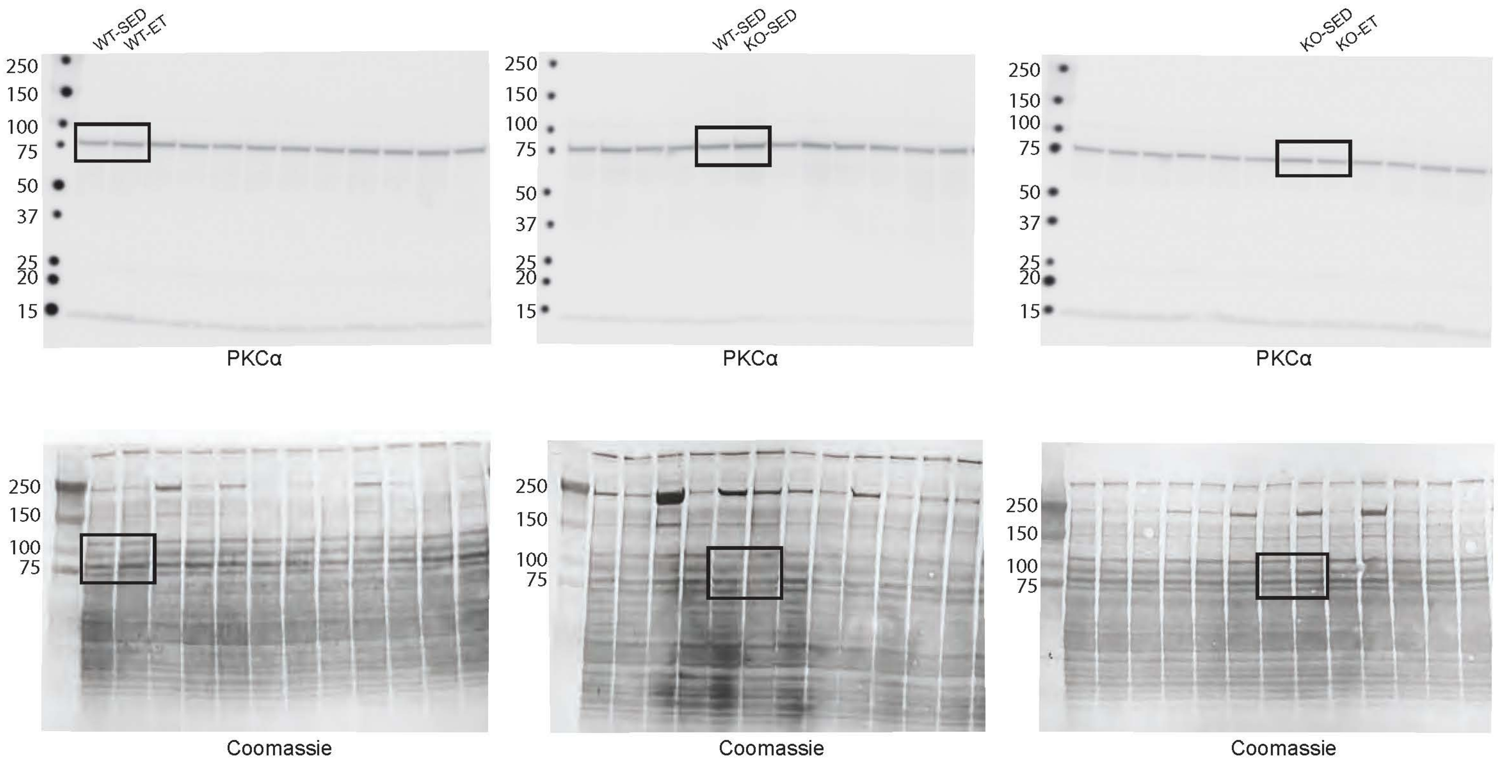

Full length blots for supplementary figure 1D

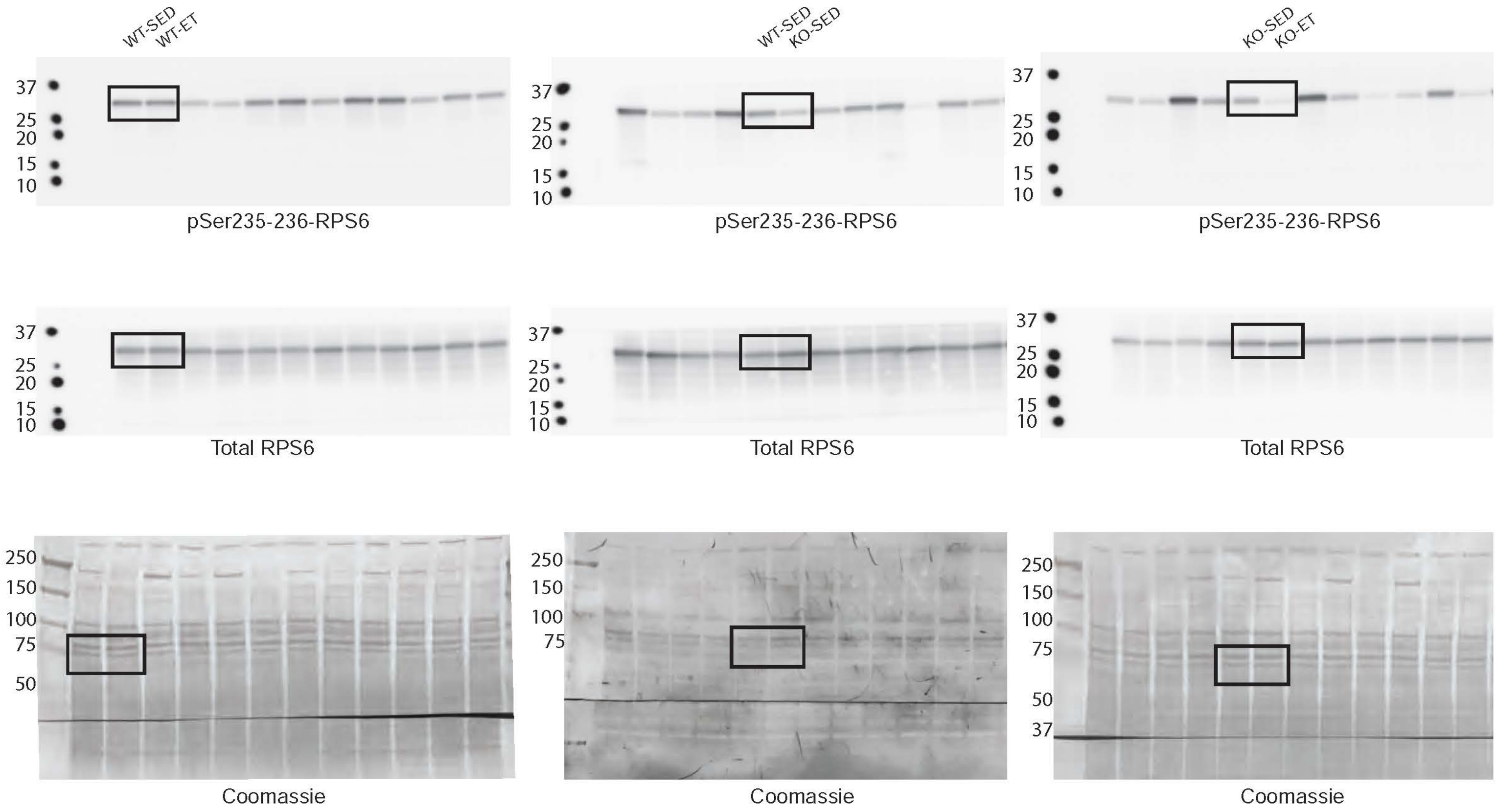

Full length blots for supplementary figure 1E

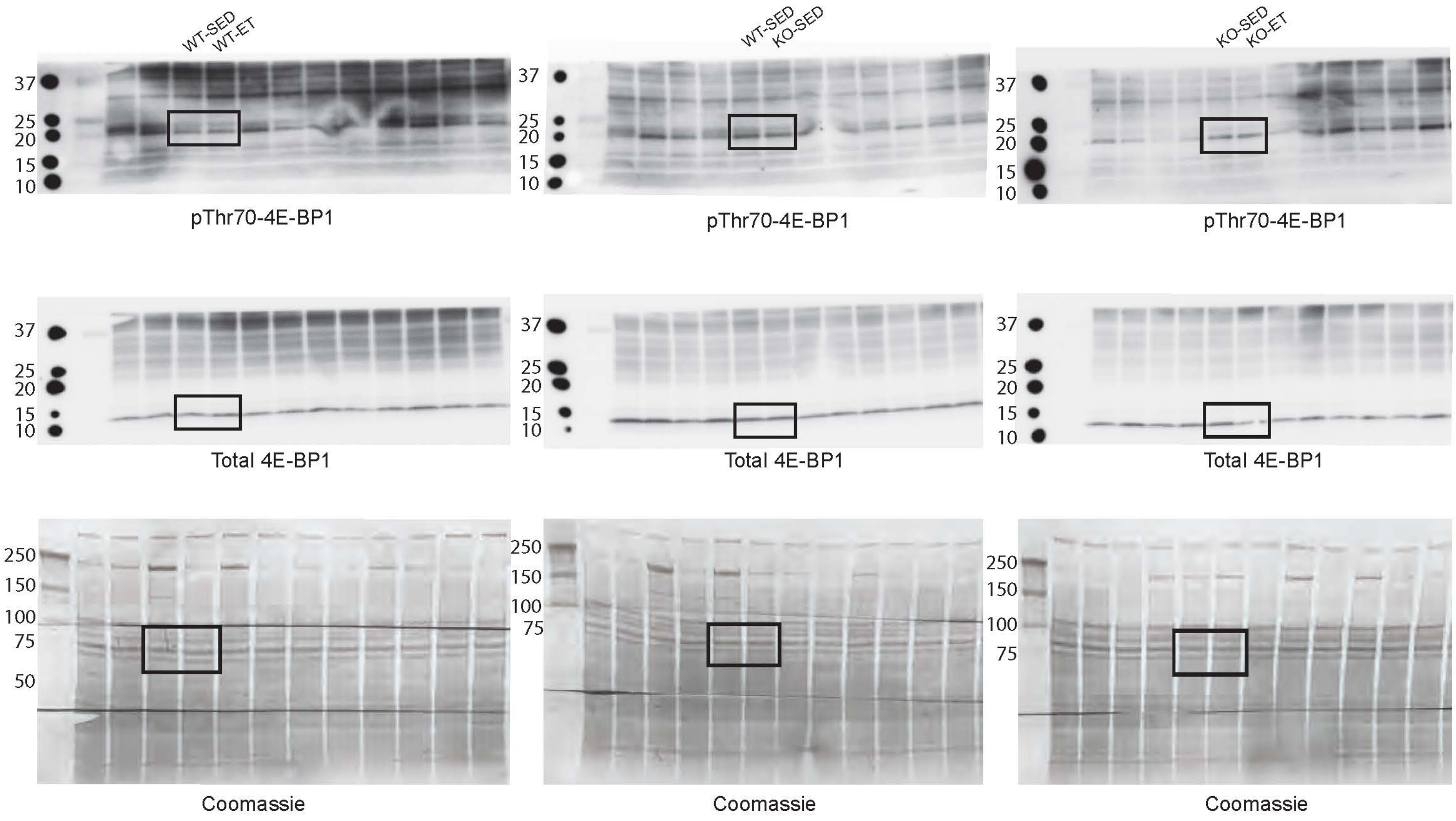

Full length blots for supplementary figure 1F

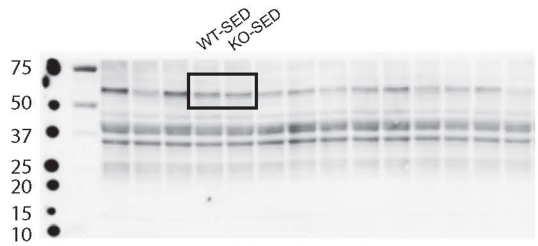

pSer473-Akt

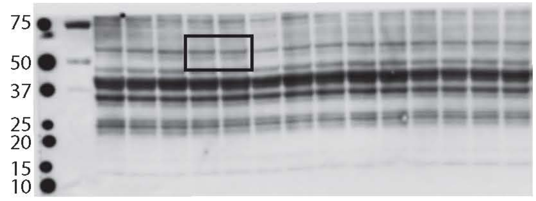

pThr308-Akt

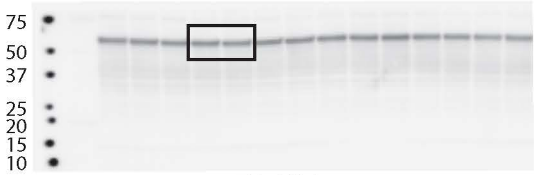

Total Akt

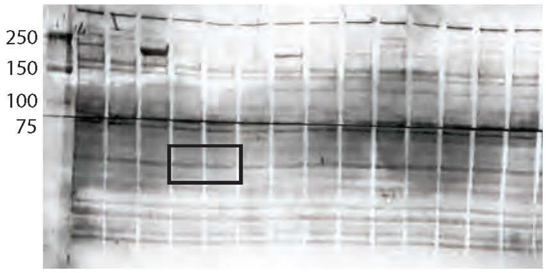

Coomassie

Full length blots for supplementary figure 1G

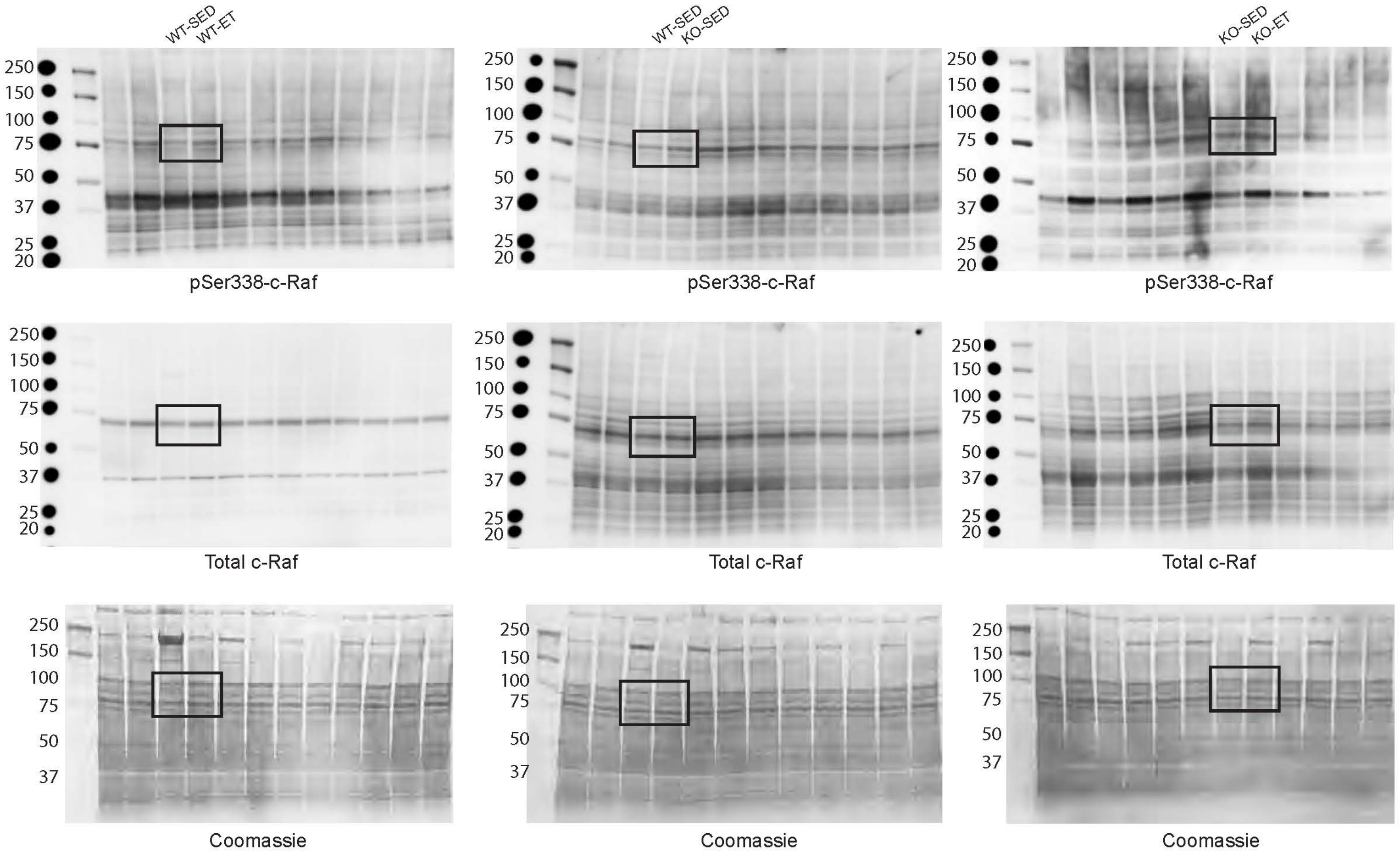

Full length blots for supplementary figure 1H

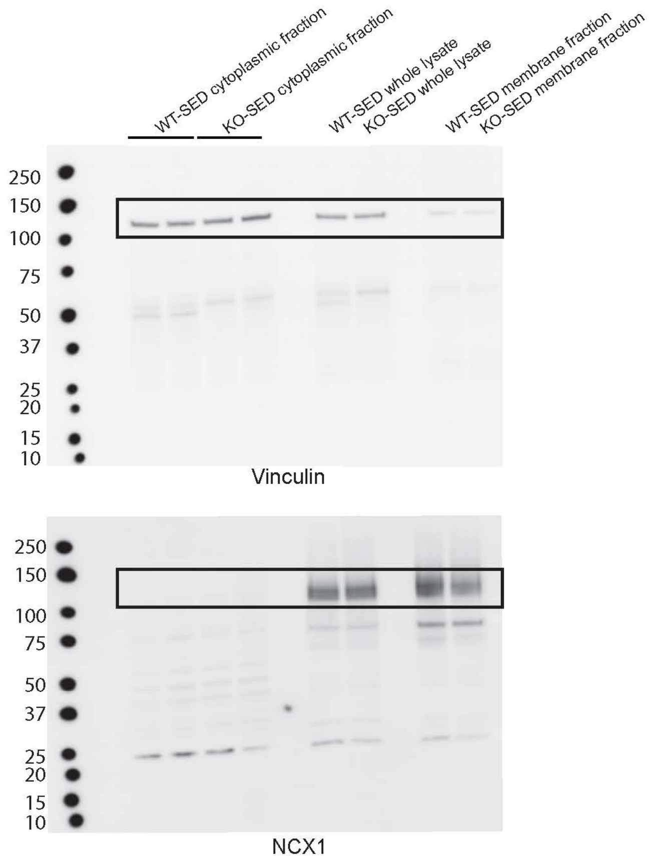

Full length blots for supplementary figure 2A

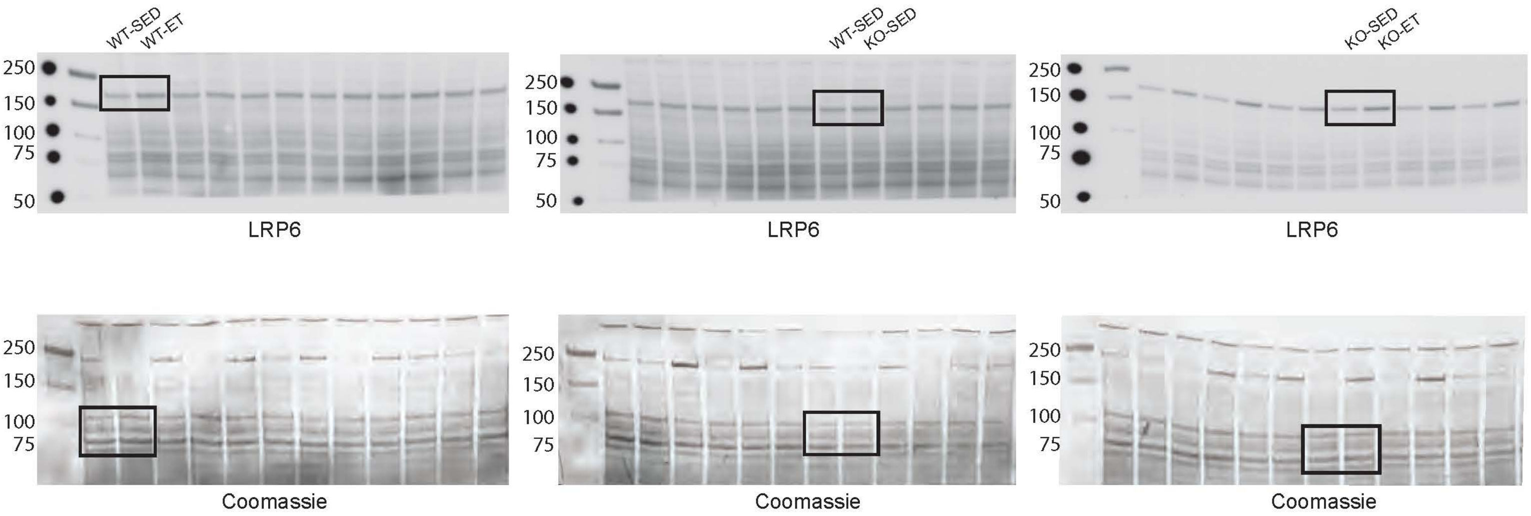

Full length blots for supplementary figure 2B

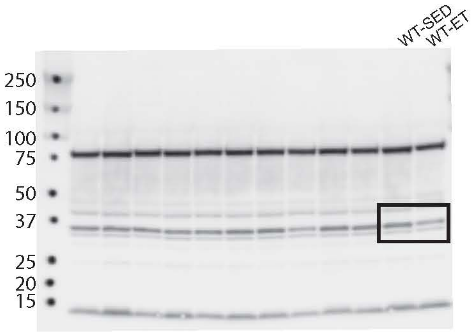

Wnt4

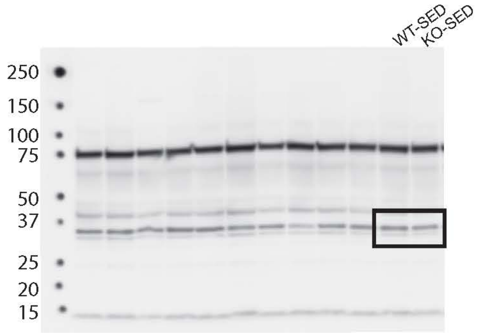

Wnt4

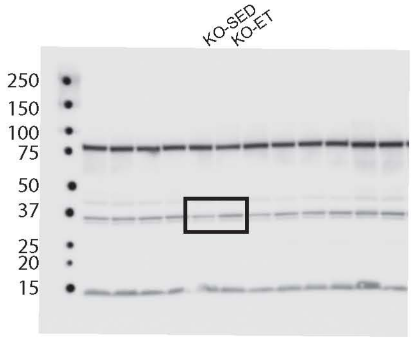

Wnt4

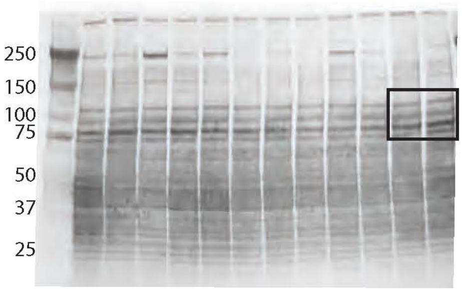

Coomassie

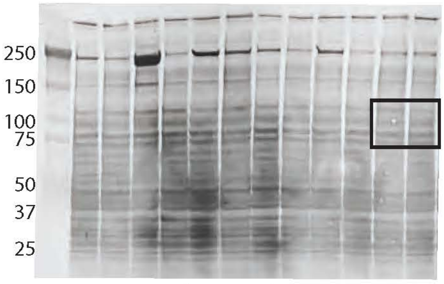

Coomassie

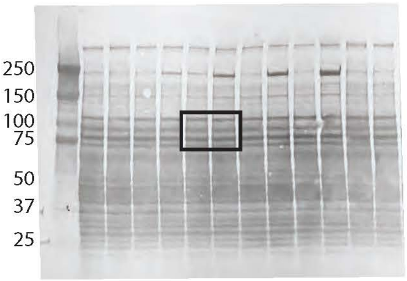

Coomassie

Full length blots for supplementary figure 2C

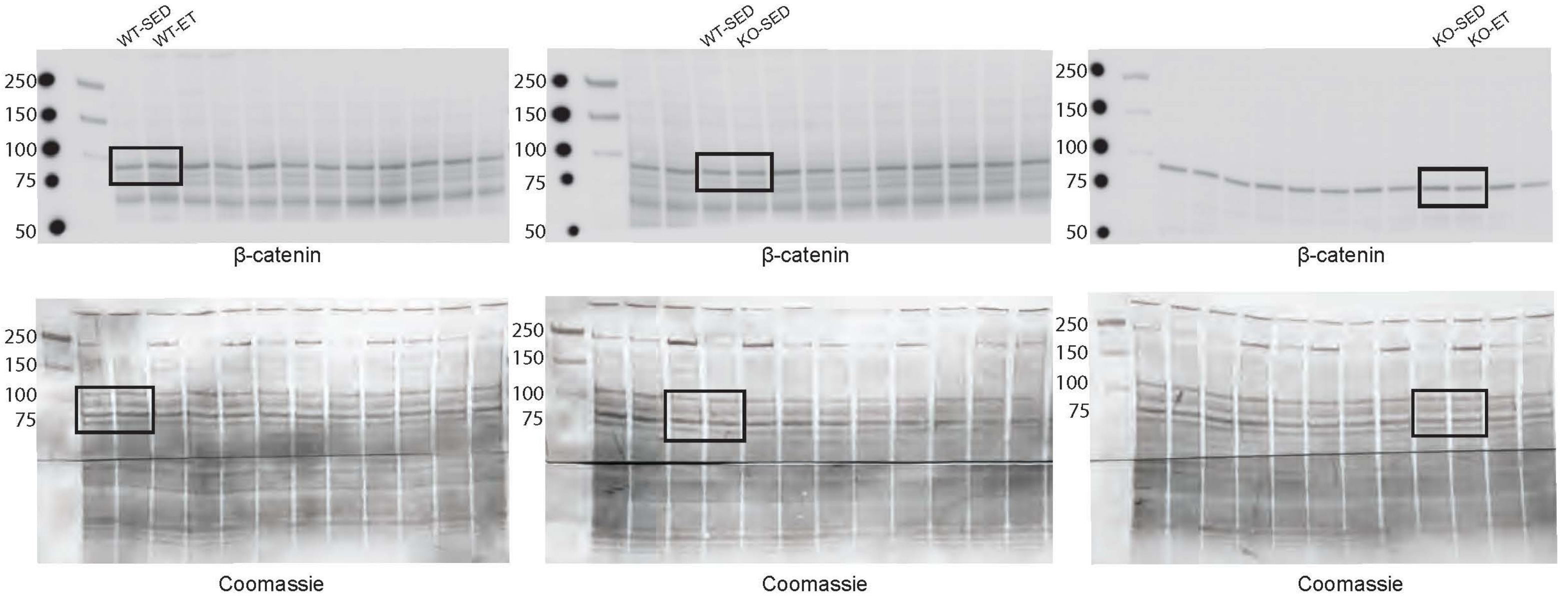

Full length blots for supplementary figure 2D

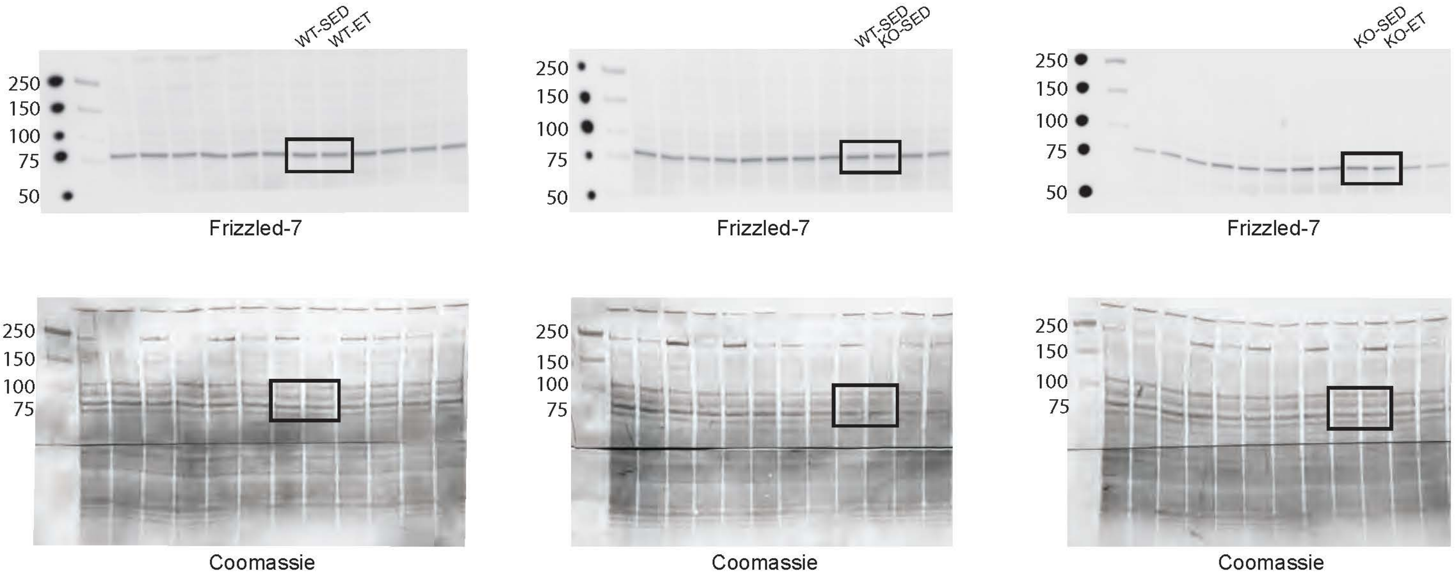

Full length blots for supplementary figure 2E

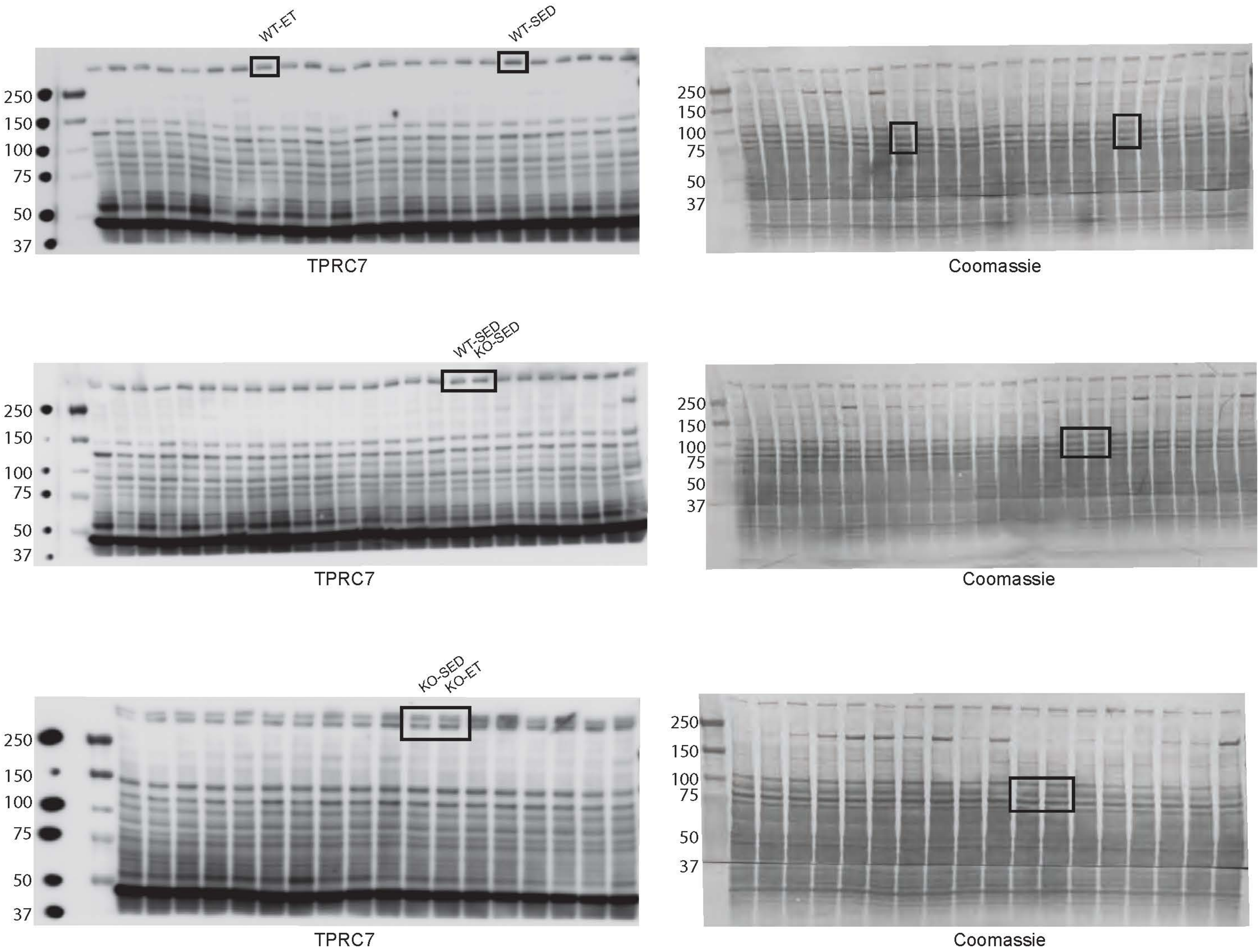

Full length blots for supplementary figure 2F

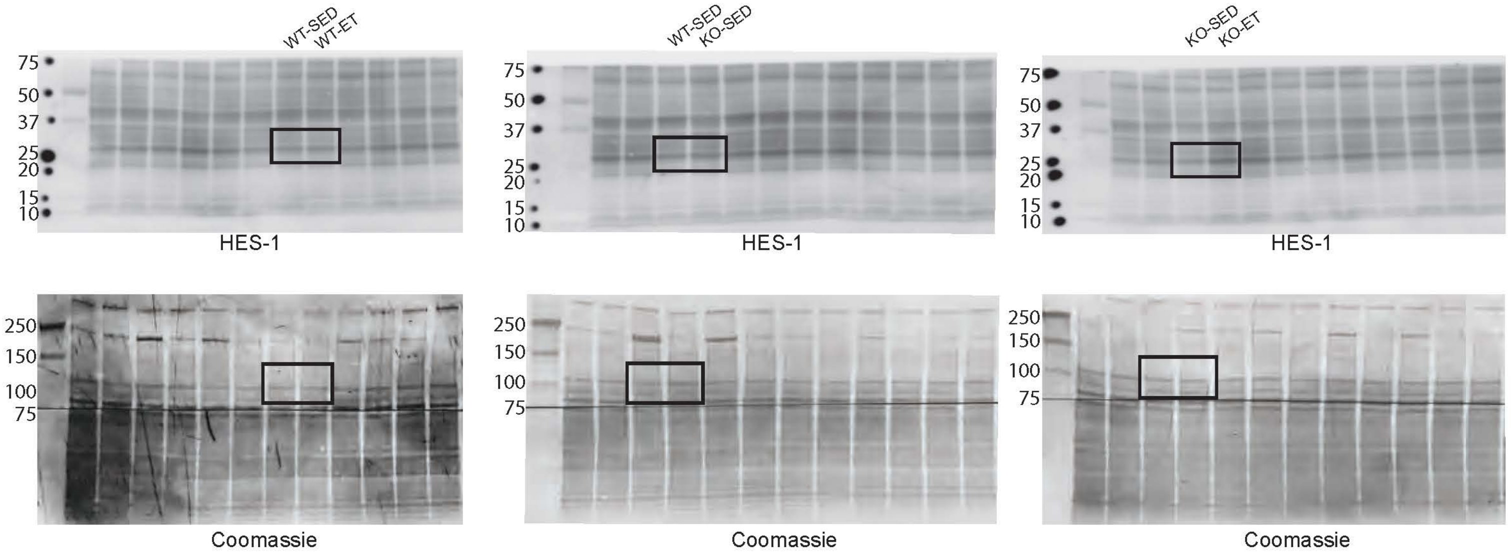

Full length blots for supplementary figure 2G

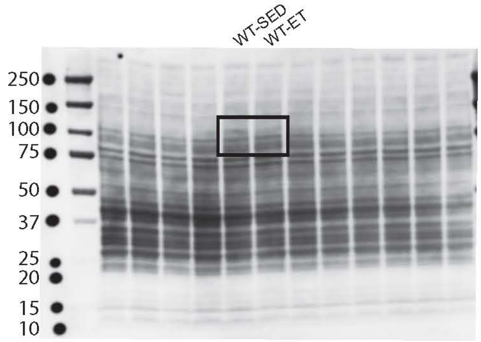

Cleaved Notch1

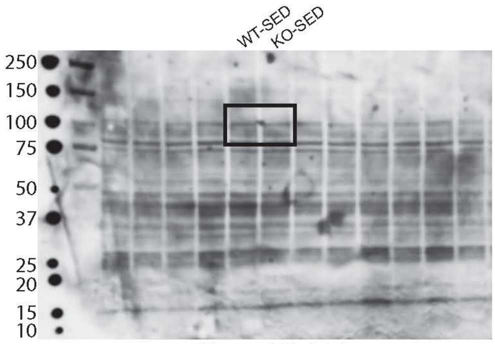

Cleaved Notch1

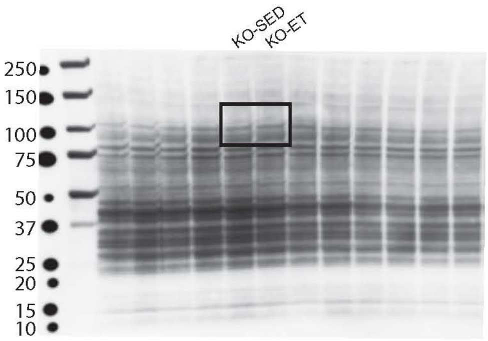

Cleaved Notch1

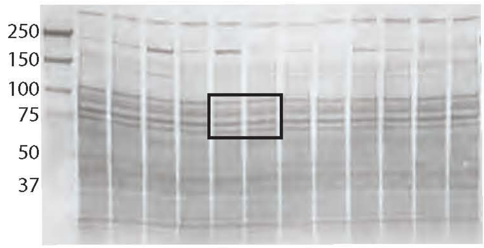

Coomassie

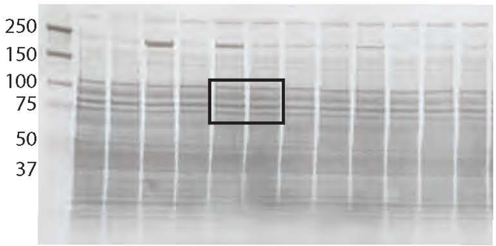

Coomassie

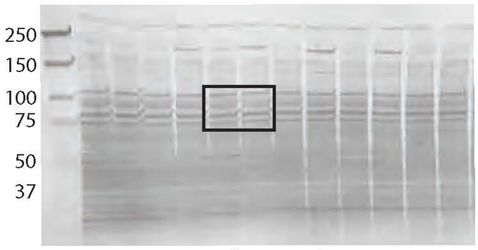

Coomassie

Full length blots for supplementary figure 2H

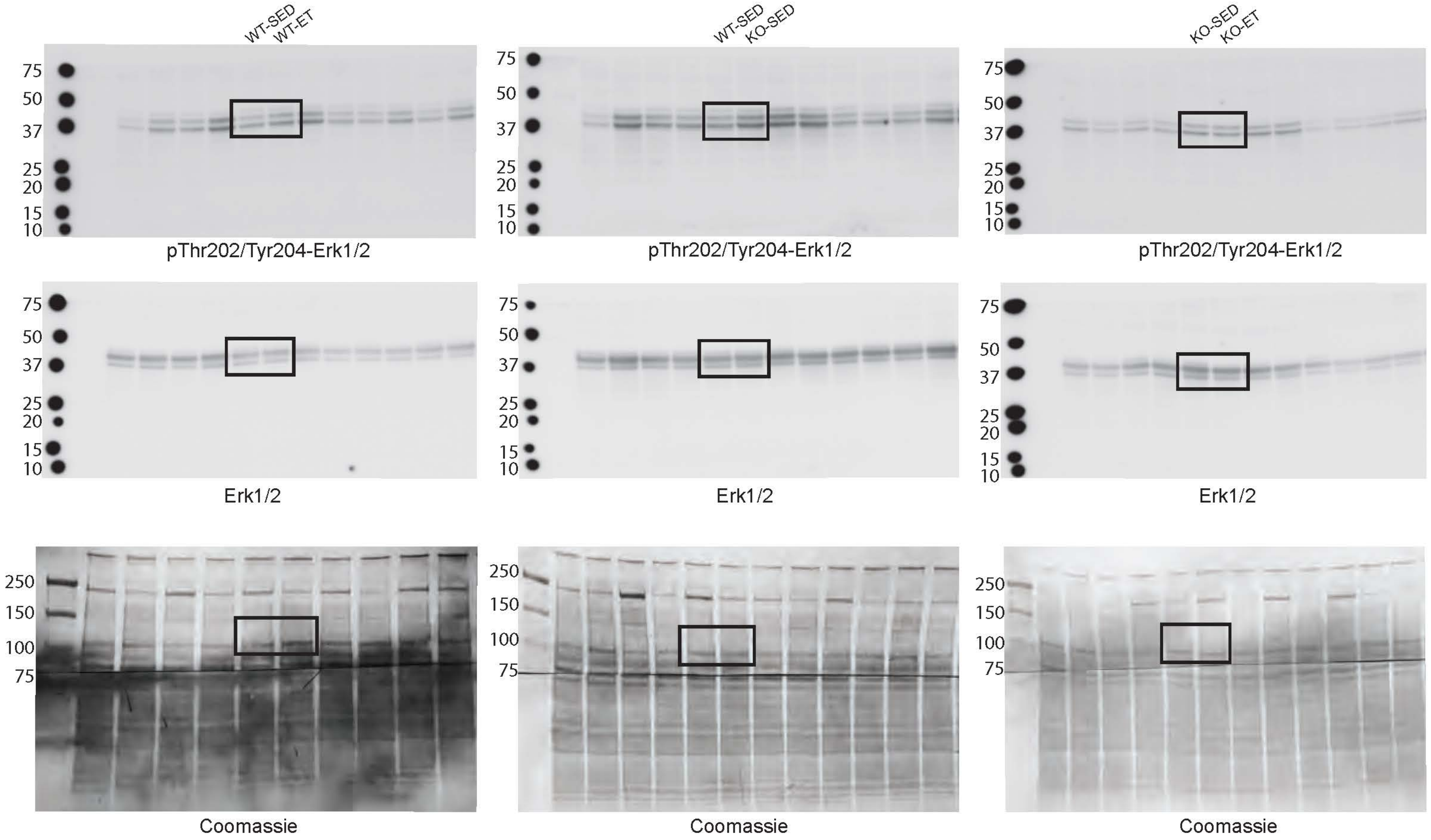

Full length blots for supplementary figure 3A

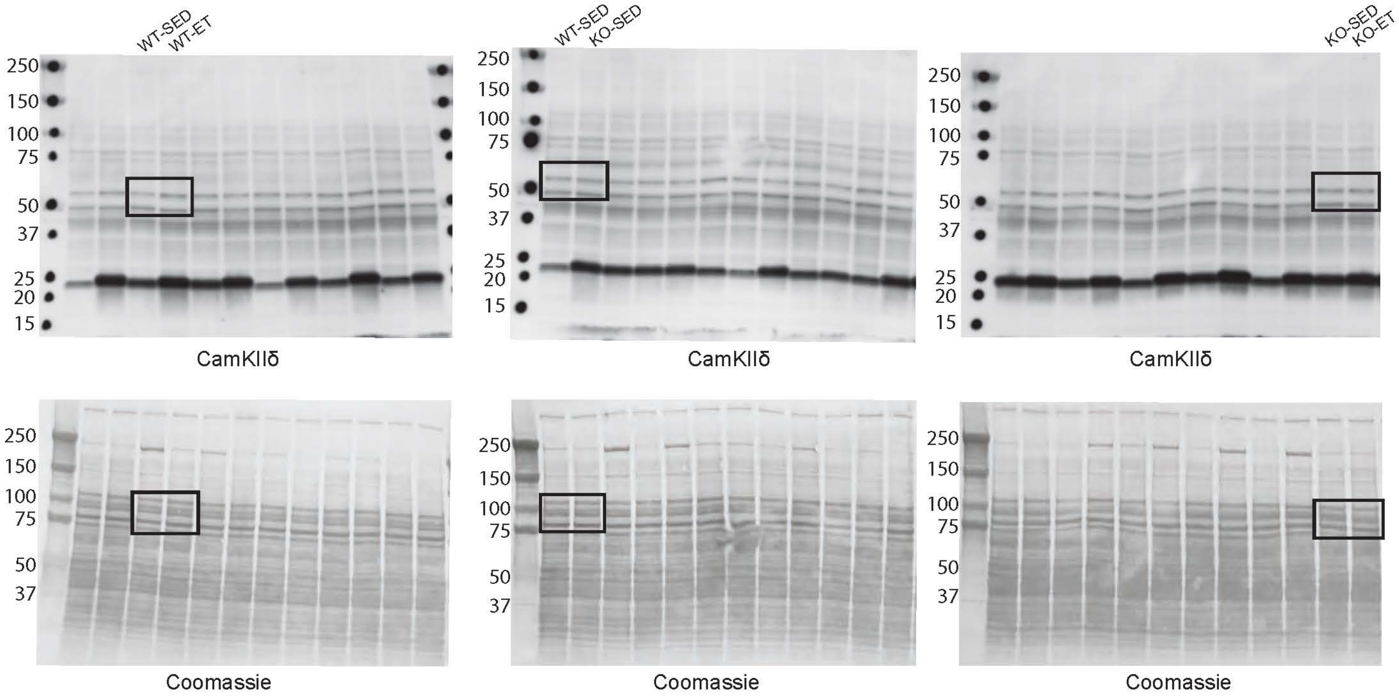

Full length blots for supplementary figure 3B

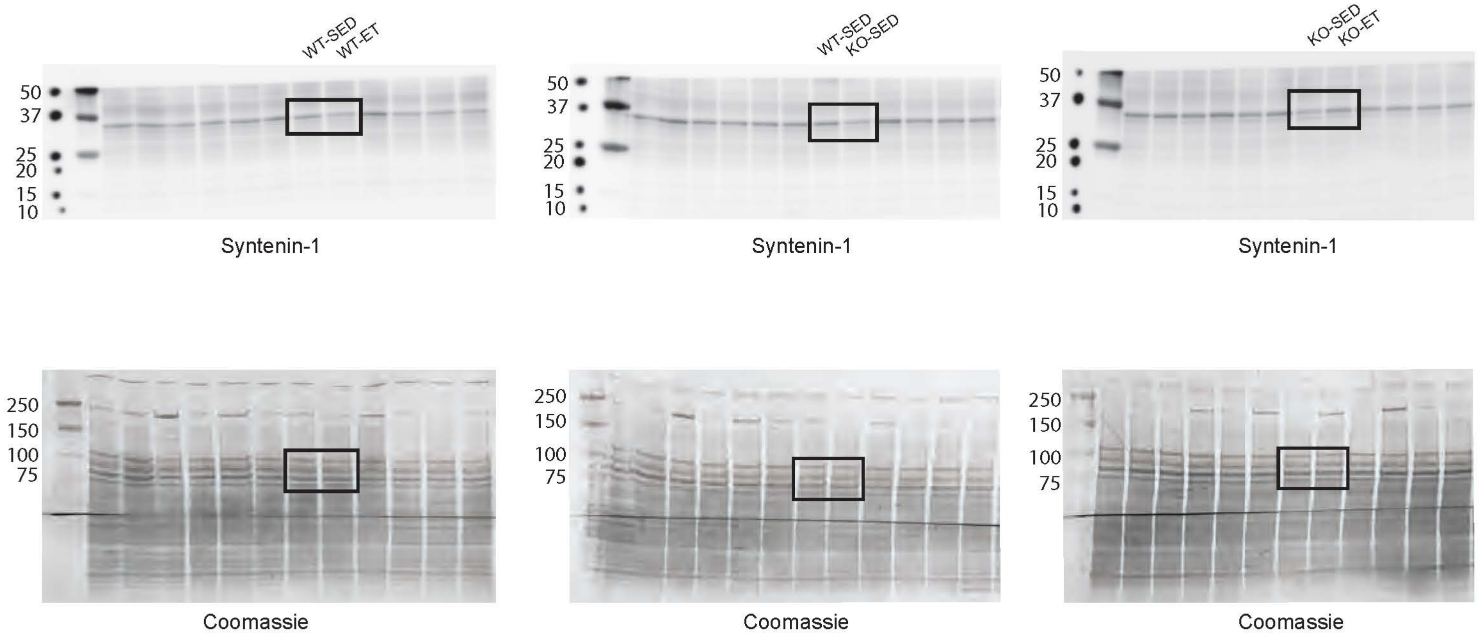

Full length blots for supplementary figure 3C

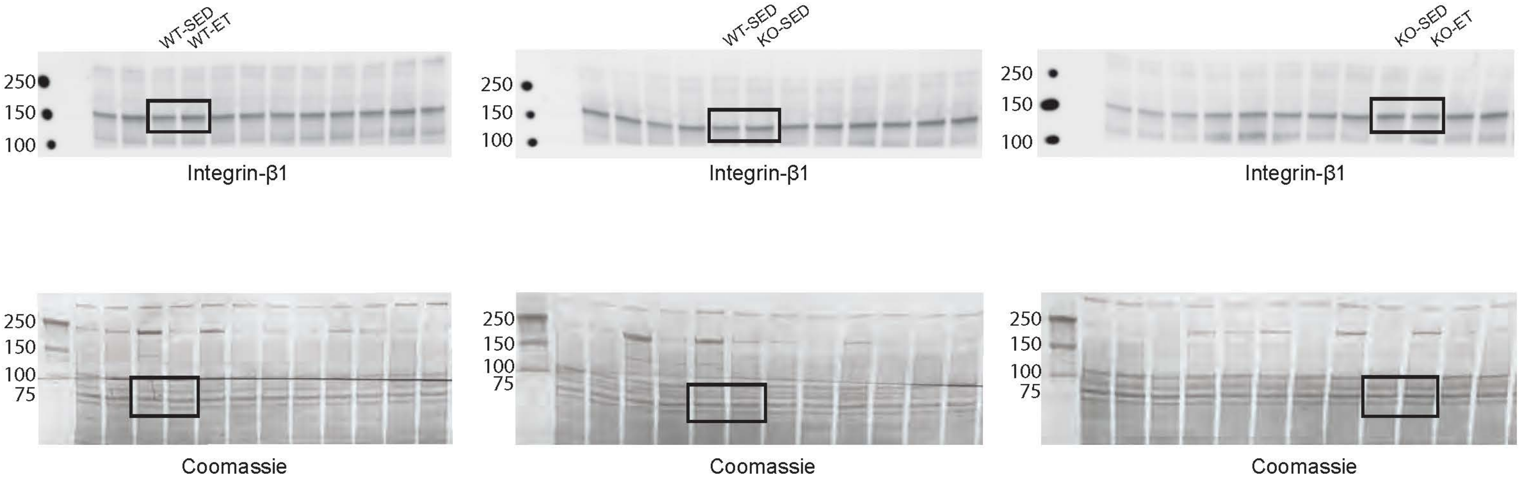

Full length blots for supplementary figure 3D

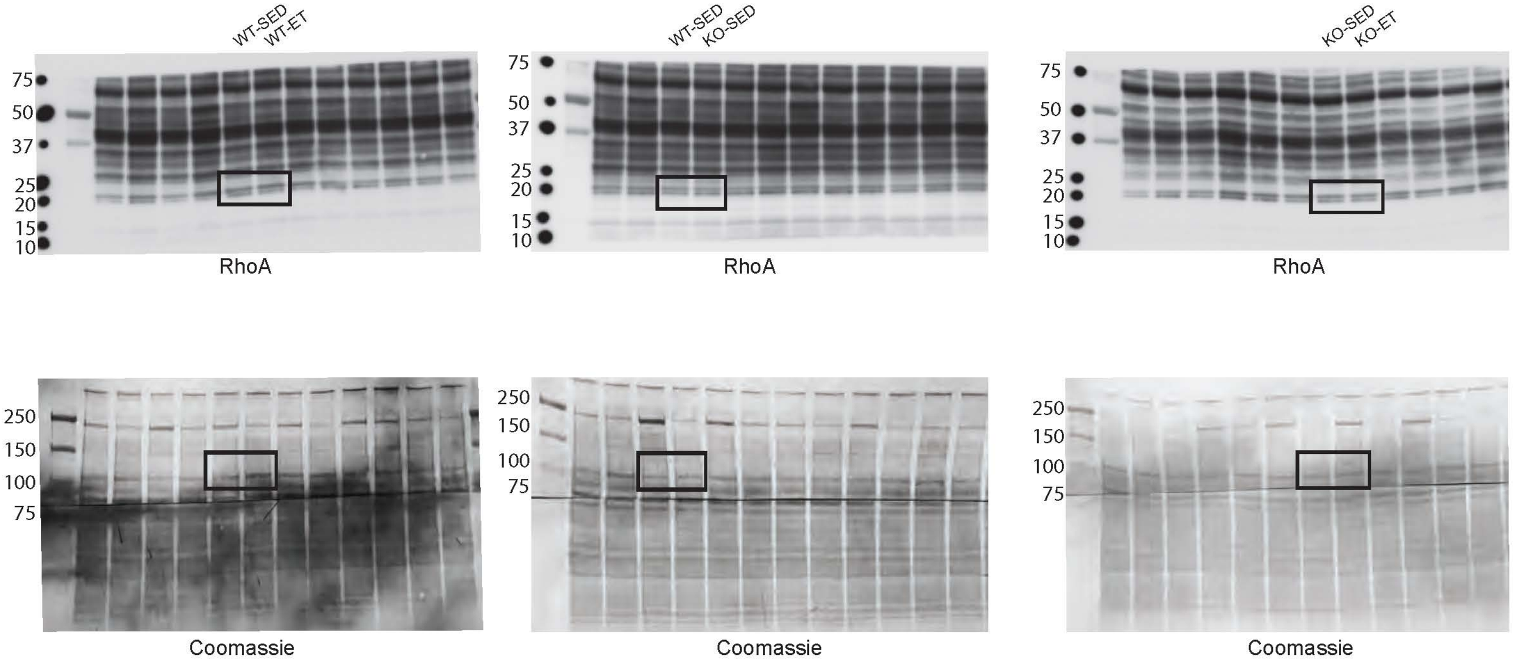

Full length blots for supplementary figure 3E

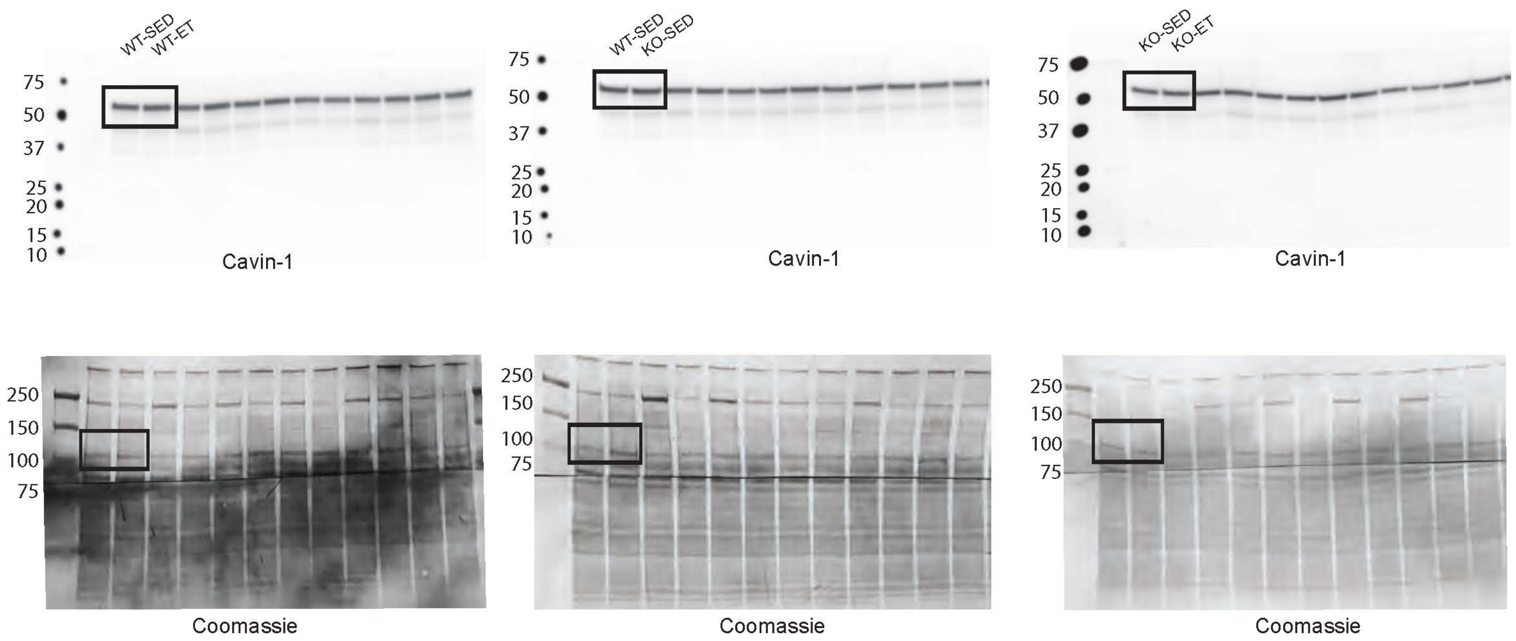

Full length blots for supplementary figure 3F

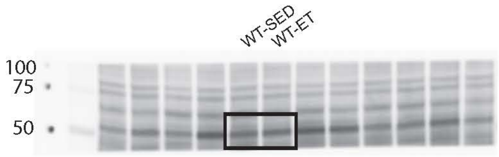

c-SRC

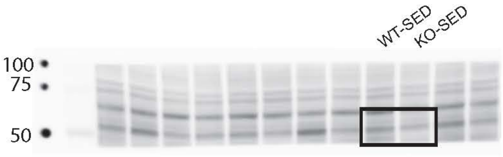

c-SRC

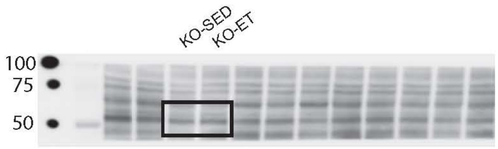

c-SRC

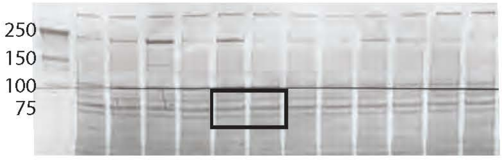

Coomassie

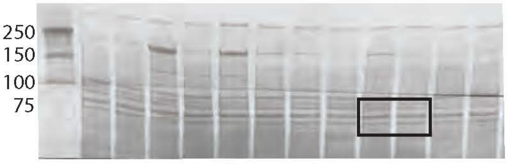

Coomassie

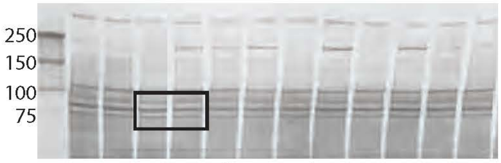

Coomassie

Full length blots for supplementary figure 3G

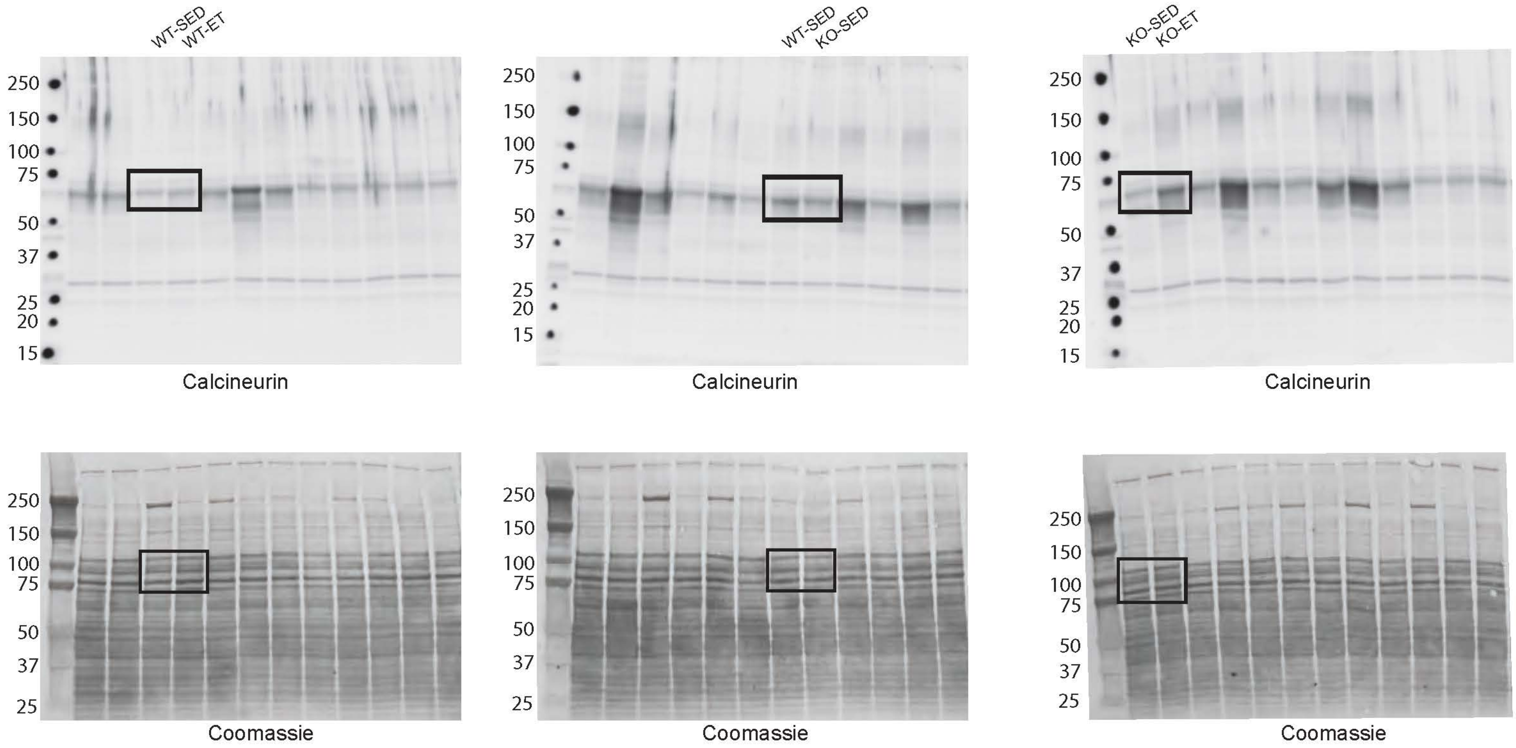

Full length blots for supplementary figure 3H

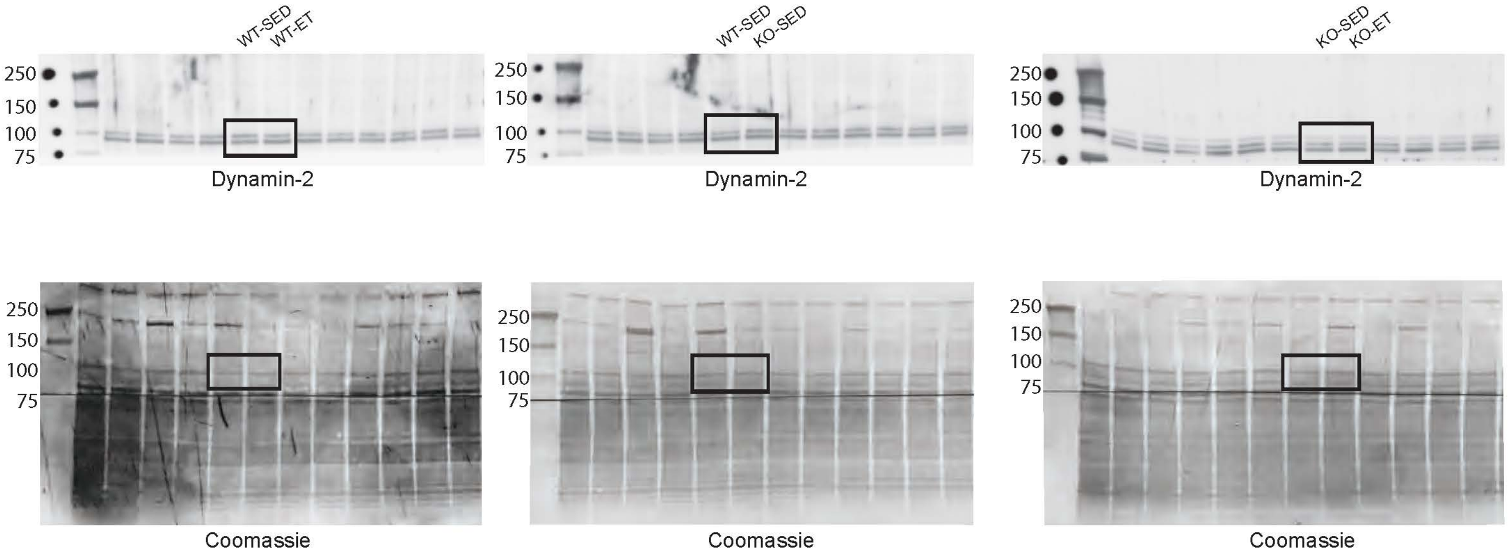

Full length blots for supplementary figure 3I

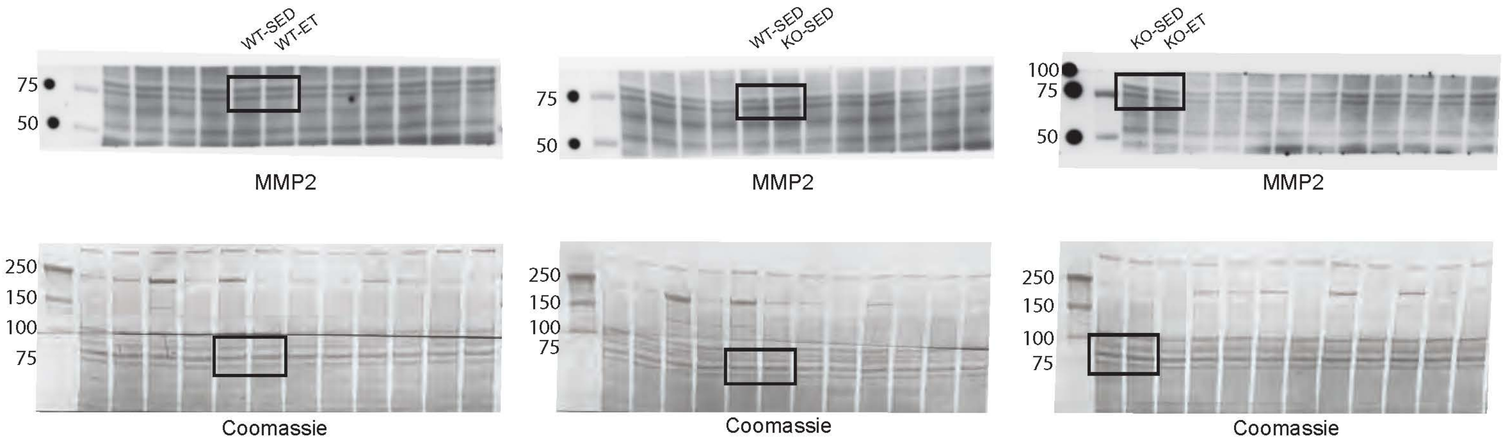

Full length blots for supplementary figure 3J

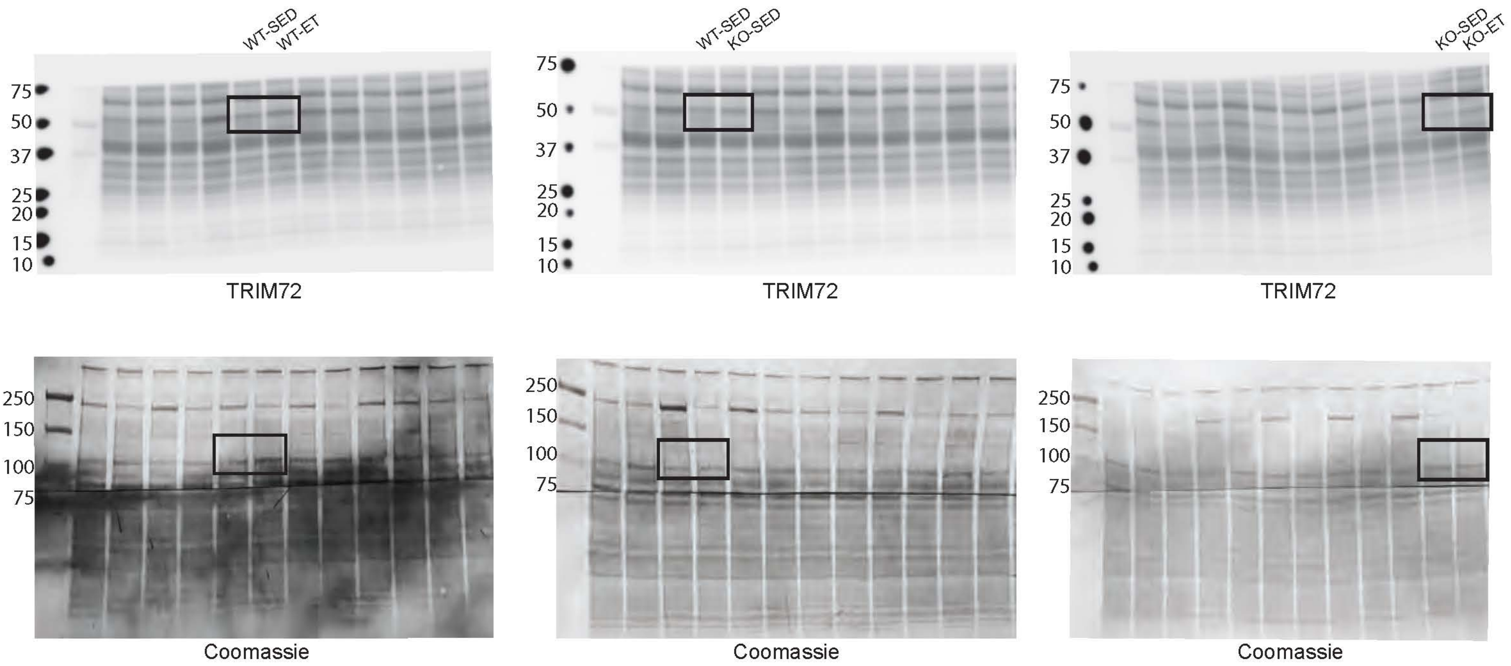

Full length blots for supplementary figure 3K

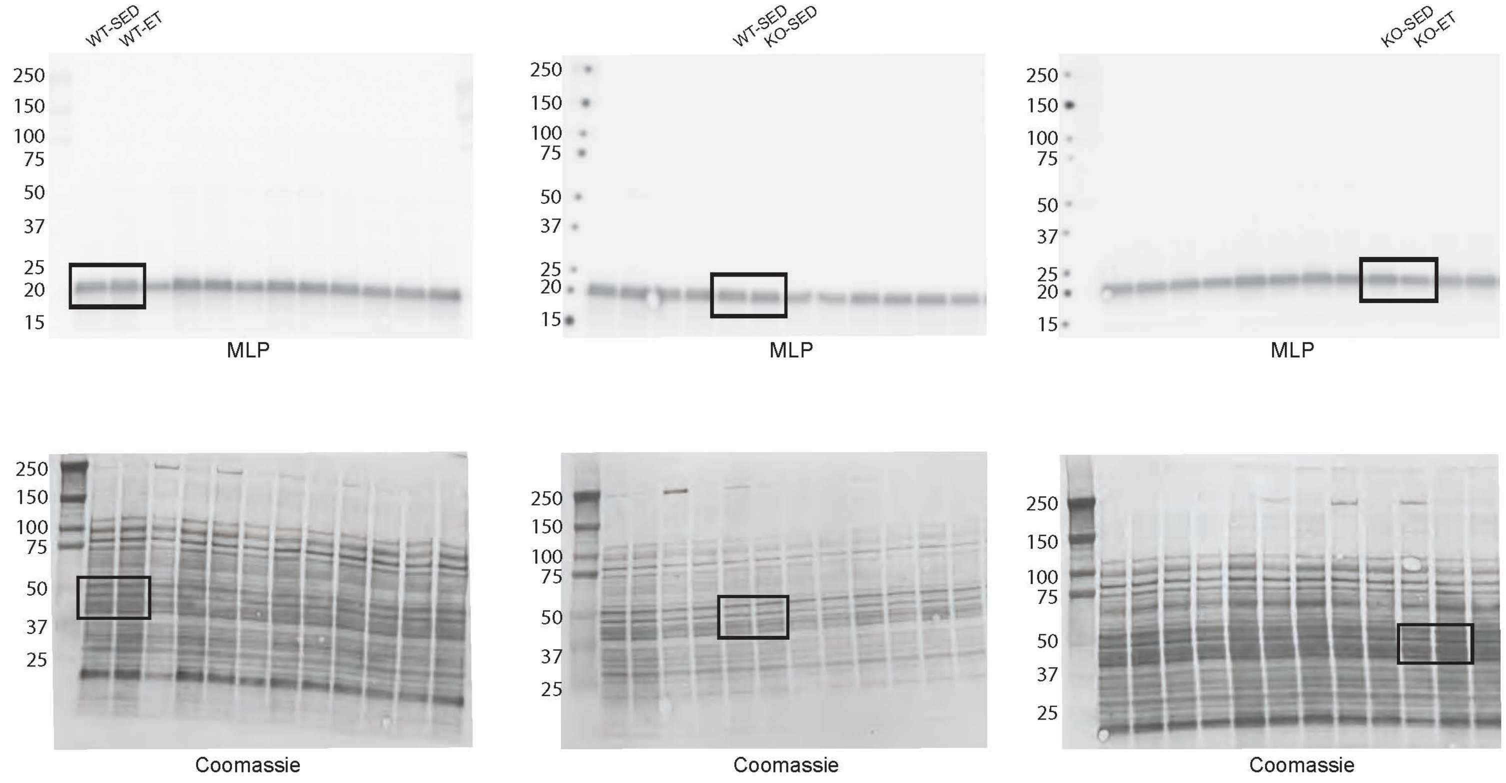

Full length blots for supplementary figure 3L

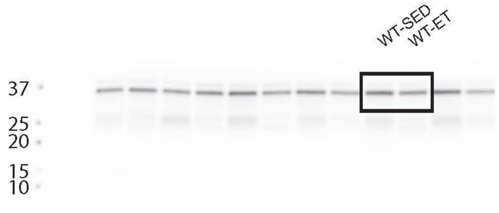

RCAN1.4

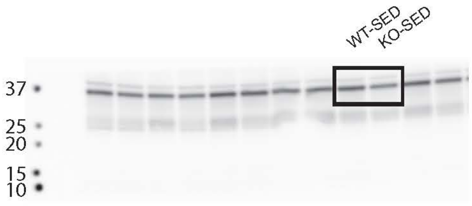

RCAN1.4

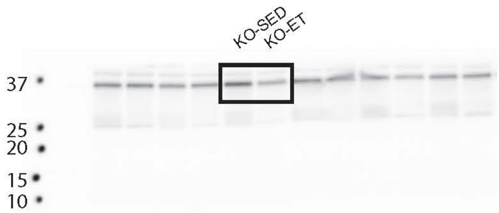

RCAN1.4

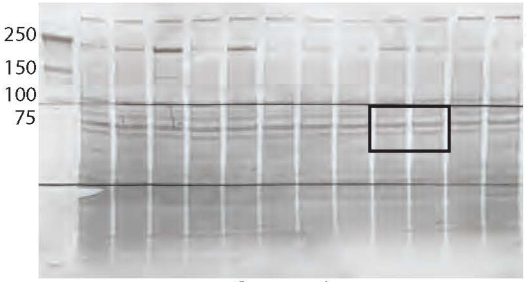

Coomassie

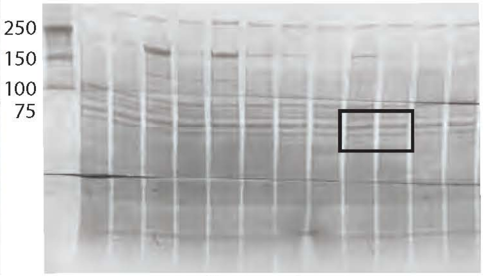

Coomassie

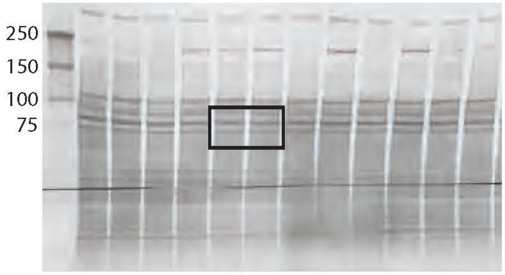

Coomassie

Full length blots for supplementary figure 3M

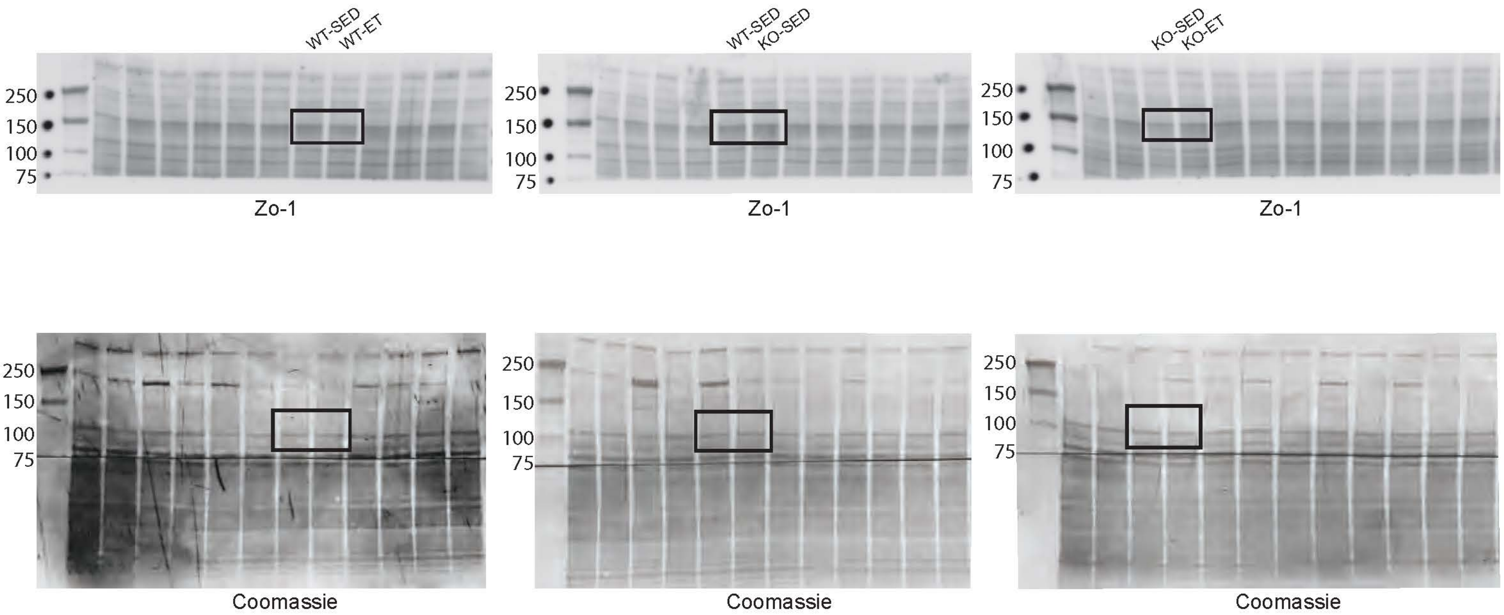

Full length blots for supplementary figure 3N

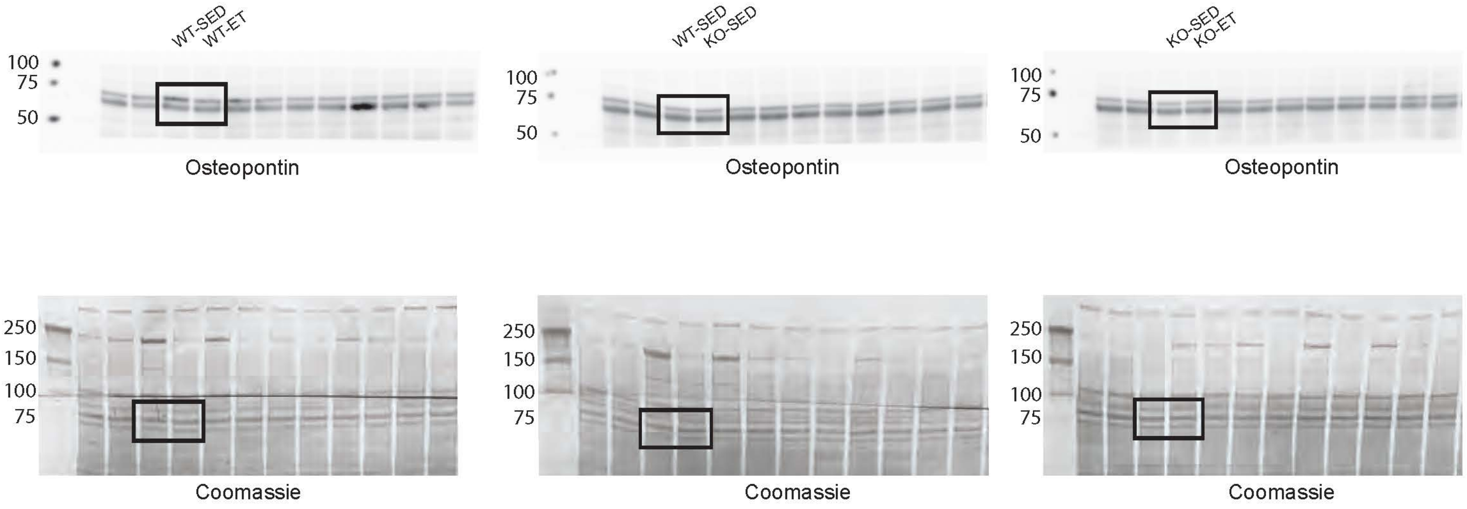

Supplement: Supplementary file 1 [file DataSheet1.PDF]
