# Supplementary material for: The female syndecan-4−/− heart has smaller cardiomyocytes, augmented insulin/pSer473-Akt/pSer9-GSK-3β signaling, and lowered SCOP, pThr308-Akt/Akt and GLUT4 levels
Source: Front Cell Dev Biol. 2022 Aug 25;10:908126. doi: 10.3389/fcell.2022.908126 (PMC9452846; doi:10.3389/fcell.2022.908126)
Supplement: Supplementary file 2 [file Image1.pdf]

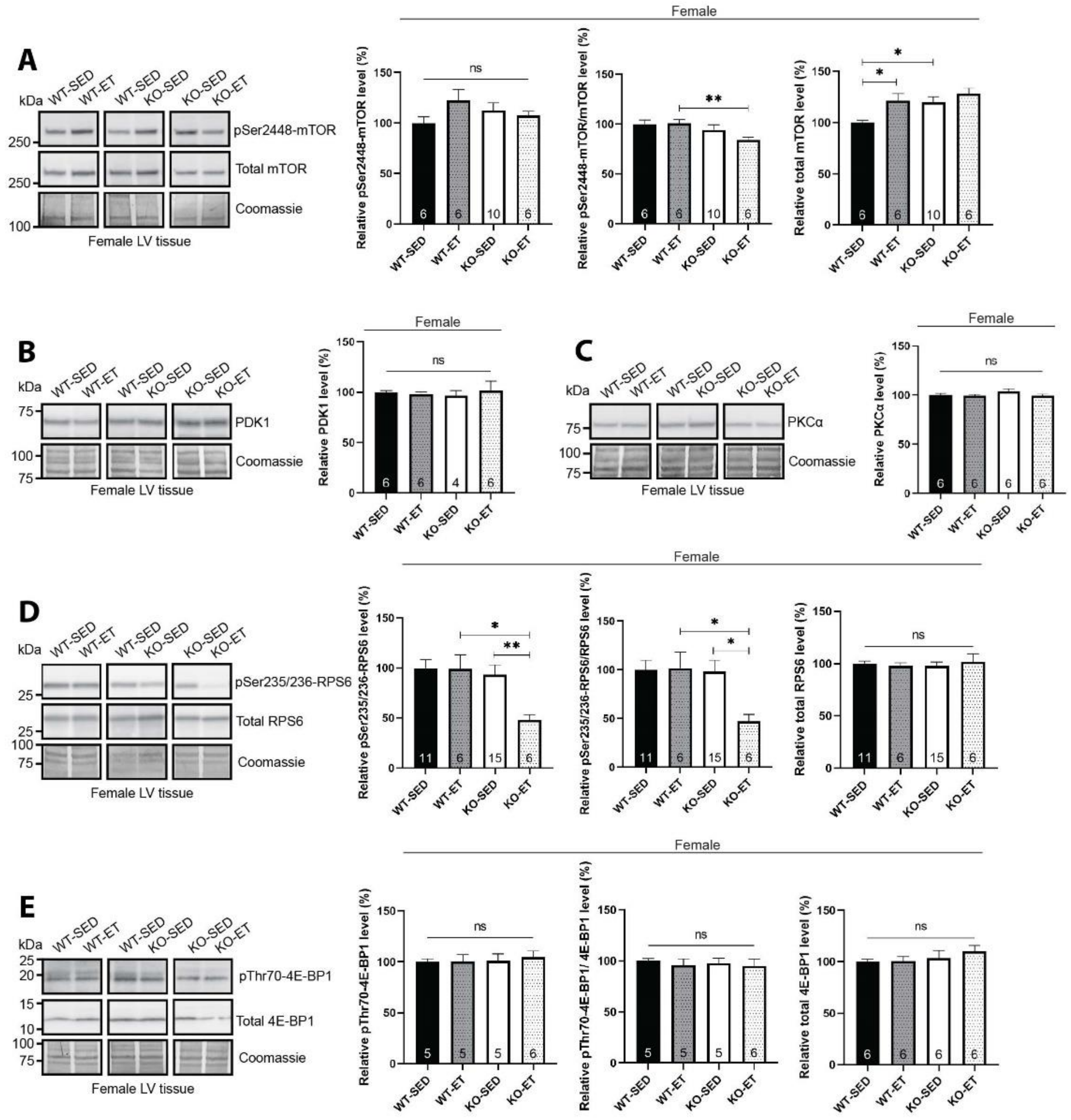

Supplementary Figure 1. Immunoblot analyses of cardiac or skeletal muscle from female and male syndecan-4$^{-/-}$ mice. Immunoblot analysis of **(A)** pSer2448-mTOR and mTOR, **(B)** PDK1, **(C)** PKCα, **(D)** pSer235/236-RPS6 and RPS6 and **(E)** pThr70-4E-BP1 and 4E-BP1 in the LV of WT-SED, WT-ET, KO-SED and KO-ET female mice. **(F)** Immunoblot of pSer473-Akt, pThr308-Akt and total Akt in skeletal muscle (*tibialis anterior*, TA) of WT-SED and KO-SED male mice. **(G)** Immunoblot analysis of pSer338-c-Raf and c-Raf in the LV of WT-SED, WT-ET, KO-SED and KO-ET female mice. **(H)** Immunoblotting of vinculin and NCX1 in total lysate and enriched cytoplasmic and membrane fractions of LV from WT-SED and KO-SED mice. Quantified immunoblot values are presented as mean percentages ± SEM, normalized to the WT-SED mice in **A-F** (n= 4-15, indicated on the graph bars). Comparison between the groups were analyzed using Mann-Whitney U tests (*p<0.05 and **p<0.01). Coomassie staining was used as loading control **(A-G)**.

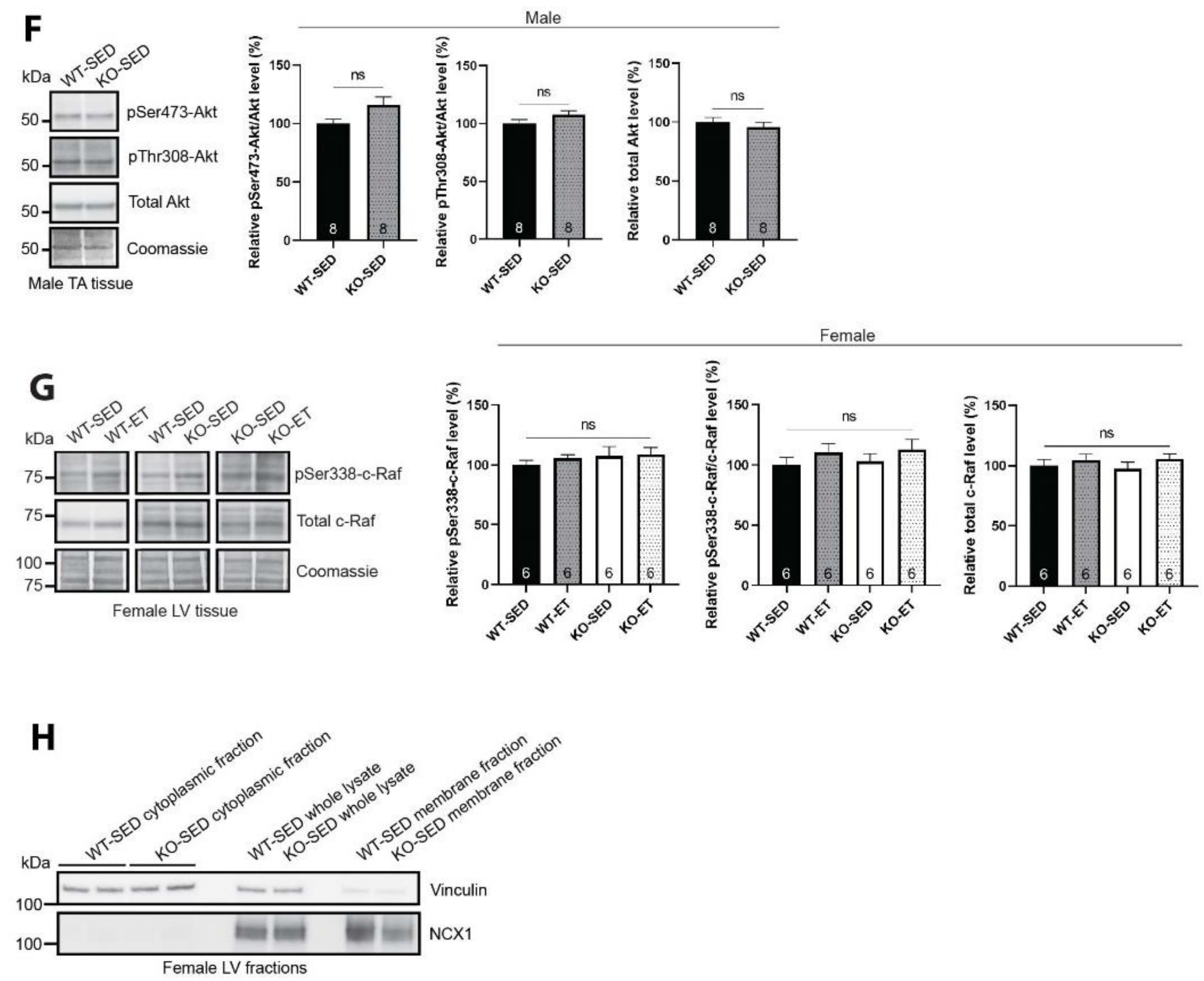

Supplementary Figure 1 continued.

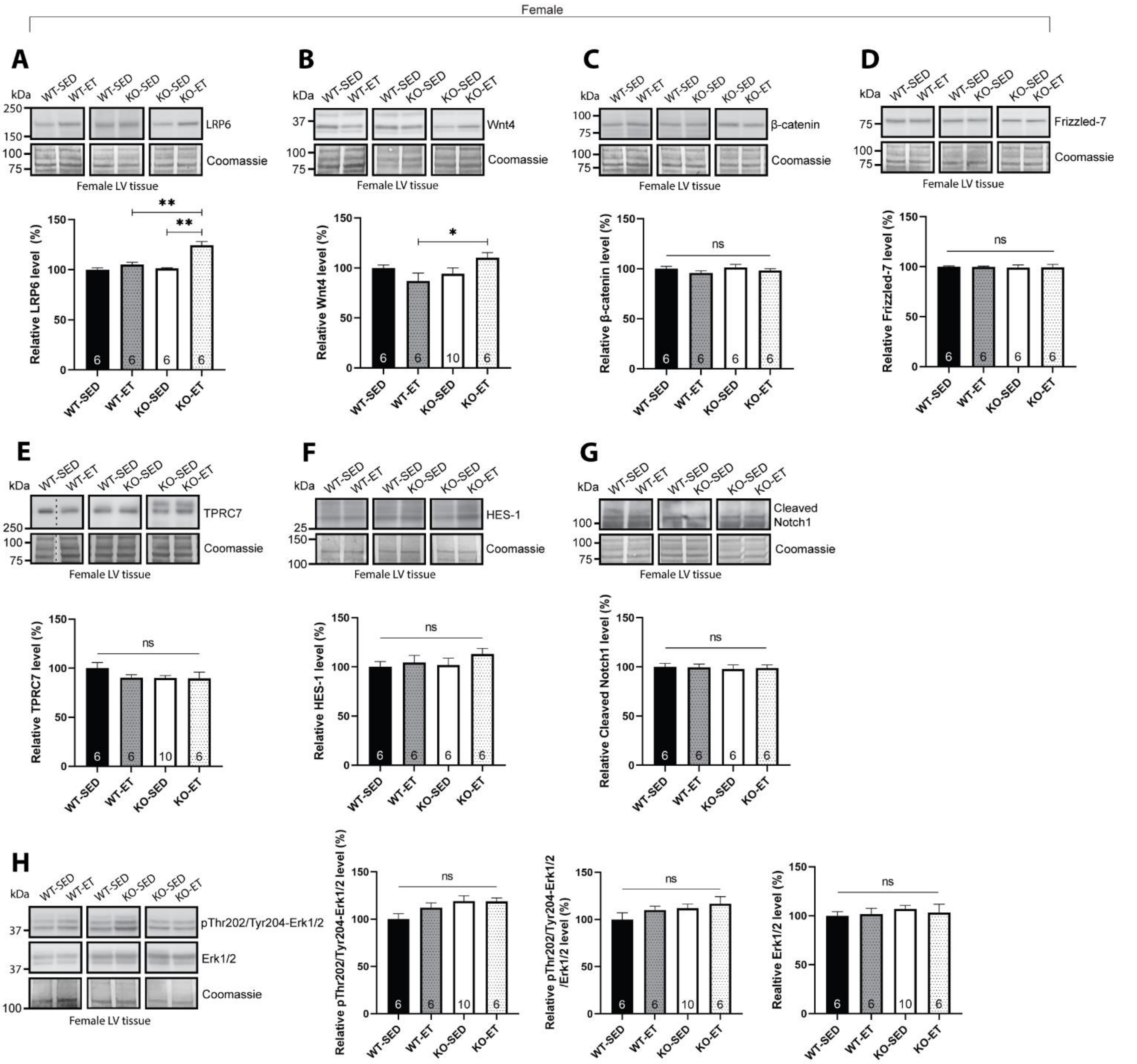

Supplementary Figure 2. **Female syndecan-4$^{-/-}$ mice have few changes in syndecan-4 associated signaling pathways.** Immunoblot analysis of **(A)** LRP6, **(B)** Wnt4, **(C)** β-catenin, **(D)** Frizzled-7, **(E)** TPRC7 (dotted line indicate representative bands are a montage from the same exposed blot), **(F)** HES-1, **(G)** Cleaved Notch1, and **(H)** pThr202/Tyr204-Erk1/2 and Erk1/2 in the LV of WT-SED, WT-ET, KO-SED and KO-ET female mice. Quantified immunoblot values are presented as mean percentages ± SEM, normalized to the WT-SED mice (n= 6-10, indicated on the graph bars). Comparison between the groups were analyzed using Mann-Whitney U tests (*p<0.05 and **p<0.01). Coomassie staining was used as loading control.

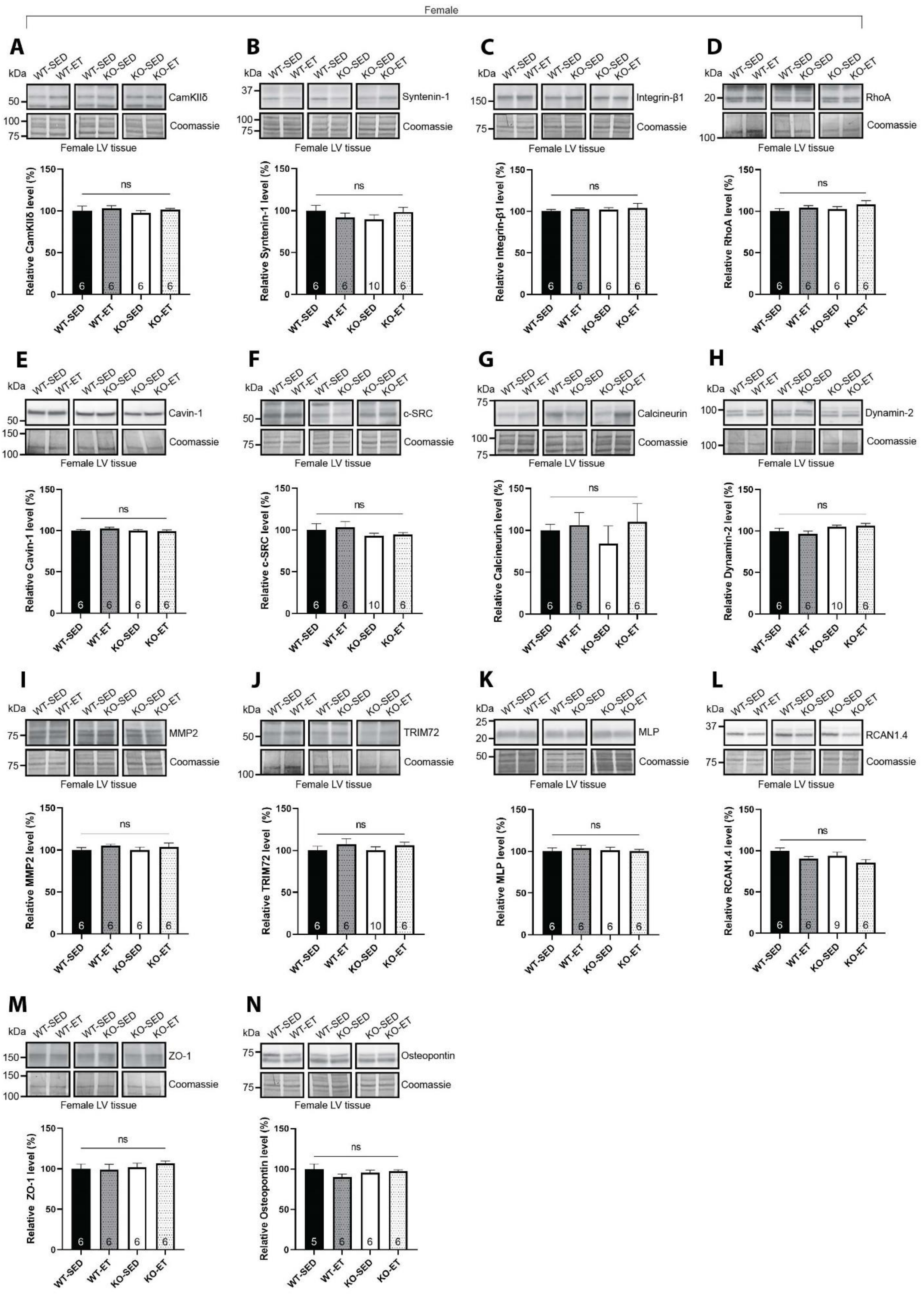

Supplementary Figure 3. Female syndecan-4$^{-/-}$ mice have no alterations in the protein levels of syndecan-4 binding partners. Immunoblot analysis of **(A)** CaMKIIδ, **(B)** Syntenin-1, **(C)** integrin- β1, **(D)** RhoA, **(E)** Cavin-1, **(F)** c-SRC, **(G)** calcineurin, **(H)** dynamin-2, **(I)** MMP2, **(J)** TRIM72, **(K)** MLP, **(L)** RCAN1.4, **(M)** ZO-1, and **(N)** osteopontin in the LV of WT-SED, WT-ET, KO-SED and KO-ET female mice. Quantified immunoblot values are presented as mean percentages ± SEM, normalized to the WT-SED mice (n= 5-10, indicated on the graph bars). Comparison between the groups were analyzed using Mann-Whitney U tests. Coomassie staining was used as loading control.
